# Implementation evaluation of an evidence-informed hospital inpatient nursing framework (HIRAID® Inpatient): a protocol for a stepped-wedge cluster RCT

**DOI:** 10.1186/s13063-025-09313-8

**Published:** 2025-12-04

**Authors:** Kate Curtis, Julie Considine, Mary K. Lam, Taneal Wiseman, Steven McPhail, Tamsin Jones, Bridie Mulholland, Baylie Trostian, Kylie Wright, Sarah Kourouche, Belinda Kennedy, Alana Clements, Geoffrey Melville, Bruce Ashford, Christopher J. Brereton, Andrew Bartlett, Timothy Wand, Christopher Pettigrew, Kathryn Zeitz, Rebecca J. Mitchell, Amith Shetty, Maria Lingam, Emma Saddington, Gerard O’Reilly, Sarah Smith, Michael Watts, Ramon Z. Shaban

**Affiliations:** 1 Faculty of Medicine and Health, Susan Wakil School of Nursing and Midwifery, The University of Sydney, Camperdown, NSW Australia; 2 Emergency Services, Illawarra Shoalhaven Local Health District, Wollongong Hospital, Crown St, Wollongong, NSW Australia; 3 School of Nursing & Midwifery and Centre for Quality and Patient Safety Research in the Institute for Health Transformation, Deakin University, Geelong, VIC Australia; 4 School of Health and Medical Sciences, Royal Melbourne Institute of Technology, Bundoora, VIC Australia; 5 Australian Centre for Health Service Innovation (AusHSI), Centre for Healthcare Transformation, Queensland University of Technology, Brisbane, Australia; 6 School of Nursing and Midwifery, Monash University, Clayton, VIC Australia; 7 Faculty of Health Sciences and Medicine, Bond University, Gold Coast, Australia; 8 Illawarra Shoalhaven Local Health District, Wollongong, NSW Australia; 9 Graduate School of Medicine, University of Wollongong, Wollongong, NSW Australia; 10 Department of Respiratory and Sleep Medicine, Wollongong Hospital, Illawarra Shoalhaven Local Health District, Wollongong, NSW Australia; 11 Faculty of Medicine and Health, Sydney Pharmacy School, University of Sydney, Camperdown, NSW Australia; 12 Nursing and Midwifery Research Unit, Illawarra Shoalhaven Local Health District, Warrawong, NSW Australia; 13 HCF Research Foundation, Sydney, NSW Australia; 14 Australian College of Nursing, Sydney, NSW Australia; 15 University of Adelaide, Adelaide, SA Australia; 16 Australian Institute of Health Innovation, Macquarie University, North Ryde, NSW Australia; 17 System Sustainability and Performance, NSW Ministry of Health, Sydney, NSW Australia; 18 Biomedical Informatics and Digital Health, University of Sydney, Sydney, NSW Australia; 19 Western Sydney Local Health District, Sydney, NSW Australia; 20 School of Nursing and Midwifery, Western Sydney University, Sydney, NSW Australia; 21 Safer Care Victoria, Melbourne, VIC Australia; 22 Monash University, Melbourne, VIC Australia; 23 School of Nursing, University of Wollongong, Wollongong, NSW Australia; 24 Faculty of Medicine and Health, Sydney Infectious Diseases Institute, University of Sydney, Camperdown, NSW Australia; 25 Research and Education Network, Western Sydney Local Health District, Sydney, NSW Australia; 26 New South Wales Specialist Service for High Consequence Infectious Diseases, New South Wales Biocontainment Centre, Western Sydney Local Health District, Sydney, NSW Australia

**Keywords:** Evidence-based nursing, Nursing assessment, Behaviour change, Clinical deterioration, Communication, Adverse events, Quality of health care, Health services research, Implementation science

## Abstract

**Background:**

Preventable adverse events in Australian hospitals are a significant safety issue, causing harm, death and increased healthcare costs. Many stem from inadequate recognition of, or response to, clinical deterioration by nurses that suggest gaps in current nursing practice frameworks. Responding to health sector demand, this study will adapt the evidence-informed HIRAID® nursing framework for use in hospital inpatient settings, implement and evaluate the standardised HIRAID® Inpatient framework to improve the quality and safety of inpatient hospital care.

**Methods:**

HIRAID® will be adapted for the hospital inpatient setting using a real-time Delphi technique and co-design with frontline hospital inpatient nurses, consumers, managers, physicians and allied health clinicians (Phase 1). An explanatory mixed methods study incorporating staff surveys and focus groups, informed by behaviour change theory, will be used to generate the implementation strategy (Phase 2).

The intervention will be trialled with 1259 nurses from 35 inpatient wards (10 hospitals) across three health services in two Australian states, using a Type II hybrid effectiveness-implementation design, including a Stepped-Wedge clustered Randomised Control Trial (Phase 3). There will be four clusters, determined by geography and clinical governance, where 4050 patients are admitted per month. These clusters were selected due to their direct admission of acutely unwell patients that are at a high risk of experiencing adverse events.

Routinely collected data, guidance documents, and pre- and post-intervention surveys of patients, carers, nursing and medical staff will be used to test the seven study hypotheses. Sample size calculations were based on prior research, assuming a 3% intra-class correlation, 5% significance (two-tailed) and 80% power, and using Woertman’s Stepped-Wedge estimate approach. Evaluation of implementation and fidelity will be assessed using the RE-AIM framework. Outcomes of interest include rates of preventable nurse-associated clinical deterioration, hospital-acquired complications, patient and carer experiences with care, and quality of nursing documentation and communication.

**Discussion:**

HIRAID® Inpatient aims to optimise nurses’ contribution to the quality and safety of hospital nursing care by preventing adverse events, improving patient experiences and strengthening clinical communication through a standardised, evidence-informed framework for assessment, decision-making and action.

**Trial registration:**

Australian and New Zealand Clinical Trial Registry, ACTRN12625000639426. Registered on 17 June 2025, https://anzctr.org.au/ACTRN12625000639426.aspx.

**Protocol version:**

Version 5.0 30 April 2025.

**Supplementary Information:**

The online version contains supplementary material available at 10.1186/s13063-025-09313-8.

## Introduction

### Background

Preventable adverse events in Australia’s hospitals, such as clinical deterioration and hospital-acquired complications (HACs), are a major ongoing safety issue [1]. These events can result in patient harm, mortality, increased length of stay, increased financial and resource burden for hospitals, and negative patient and family experiences [2]. They are, however, largely preventable [3]. Despite the widespread implementation of rapid response systems that facilitate early recognition and response to deteriorating patients, rates of clinical deterioration and HACs remain static, often due to delays or failures in recognition and/or response to clinical deterioration [4]. Up to 80% of rapid response team (RRT) calls are preceded by objective signs of clinical deterioration present for many hours before RRT activation [5].

Current patient assessment frameworks used by nurses’ target deviations in vital signs, the assessment of specific body systems, identification of specific risks (e.g. falls, pressure injuries, delirium) and recognition of the deteriorating patient [6]. Although useful, the piecemeal use of these frameworks hinders nurses’ capacity to perform a thorough assessment of the entire patient status [7], which, in turn, does not prevent patient deterioration [8]. With improvements to patient assessment frameworks, inpatient nurses could better address the increasingly complex health and well-being needs of patients.

Nurse-led frameworks that improve patient outcomes in emergency care, improve the quality of clinical assessment of patients [9], and reduce urinary tract infections [10]. However, a review of the international literature shows the absence of a standardised, evidence-based nursing framework for nurses in hospital wards or inpatient settings [8]. Moreover, a large evidence-based nursing core assessment, the ENCORE Queensland trial (2024), underscored the necessity to strengthen nursing assessment for complex patients in hospital wards [11]. To be successful, an inpatient nursing framework must facilitate the assessment of the entire patient [8]. We propose a new ‘whole-of-patient’ nursing framework that can be tailored to any hospital inpatient setting.

### Rationale

Despite considerable attention existing inpatient assessment nursing frameworks are not optimising efforts to prevent clinical deterioration. In Australian hospital wards, nurses’ approaches to, and practices of, patient assessment are inconsistent, resulting in significant avoidable patient deterioration and unwarranted variation in care [8].

**Fig. 1:**
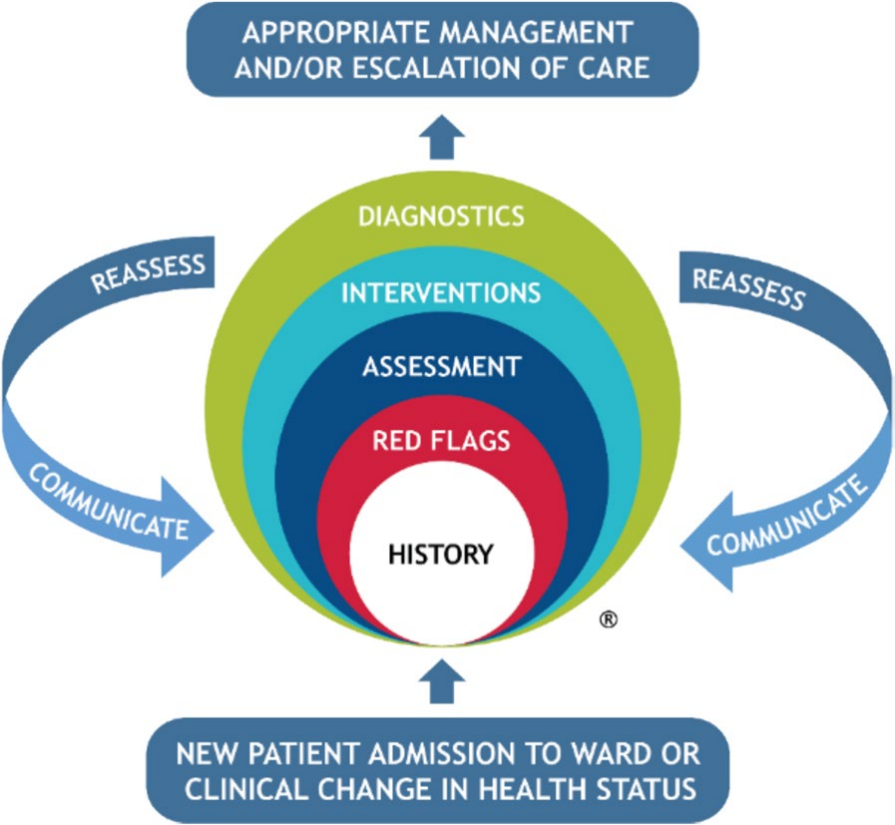
Preliminary conceptual framework for HIRAID® Inpatient

The aim of this paper is to describe the methods to be used to address nurses’ inconsistent approaches to, and practices of, patient assessment by adapting the eight-element HIRAID® framework (History including Infection risk, Red Flags, Assessment, Intervention, Diagnostics, reassessment, communication and plan) for the inpatient context. HIRAID® was first developed for emergency departments (ED) and provides a holistic and structured approach to nursing assessment and management that improves the quality and consistency of nurses’ judgement, practice and documentation (Fig. 1). In EDs, HIRAID® significantly improved nurses’ clinical care, documentation and handover. HIRAID® also significantly decreased patient deterioration related to nursing care, treatment delays, failure to escalate care of deteriorating patients and resulted in healthcare cost avoidance [7, 12–14]. With adaptation of HIRAID® ED for the inpatient setting, we envisage similar benefits for inpatient settings.

#### Study design

**Fig. 2:**
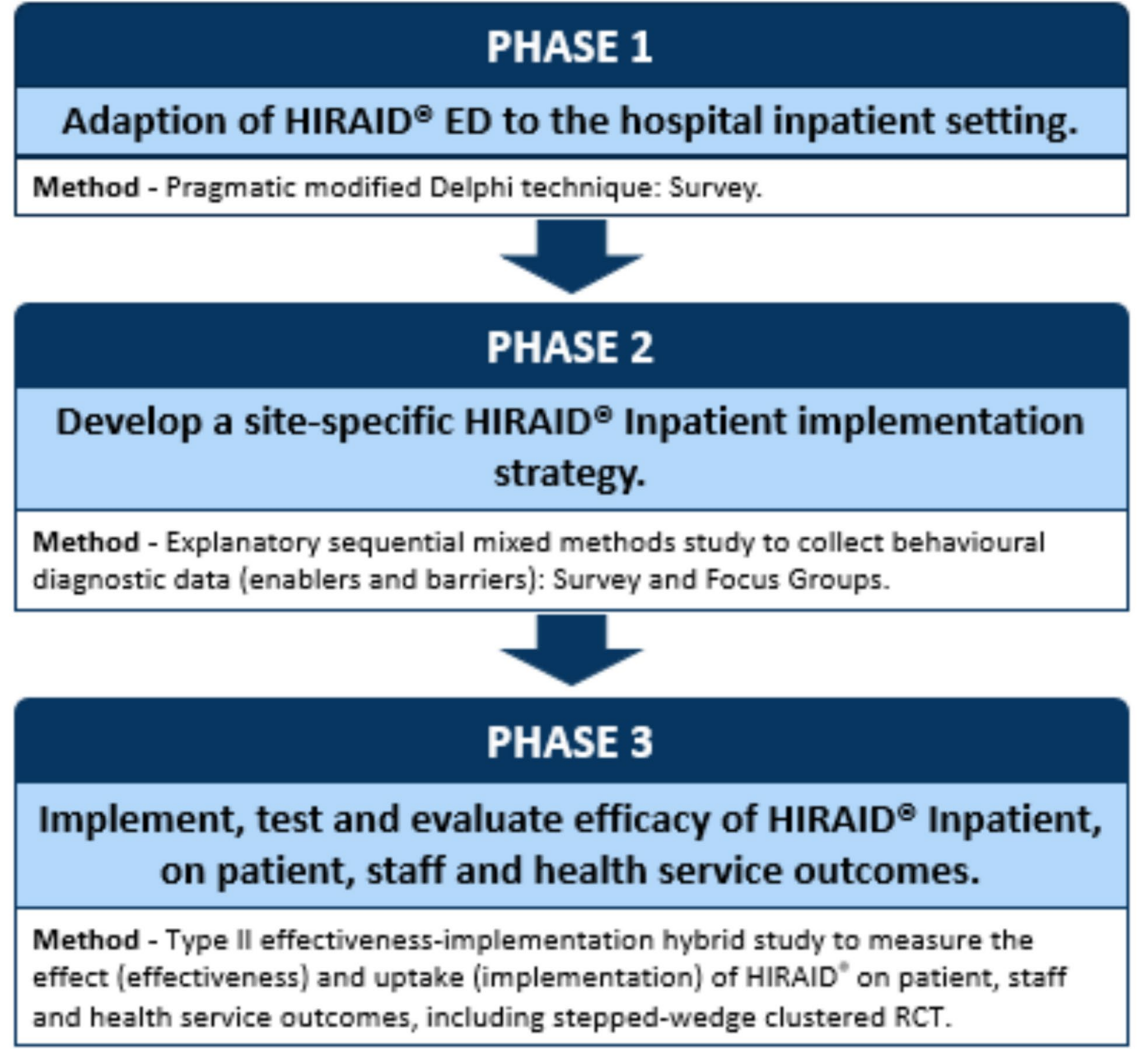
Overarching study design and methods for HIRAID® Inpatient

Implementation and evaluation of HIRAID® Inpatient will be undertaken in three phases (Fig. 2). In Phase 1, the existing HIRAID® emergency nursing framework will be adapted and recontextualised for inpatient care using a pragmatic modified Delphi technique. In Phase 2, an explanatory sequential mixed methods study design will use behaviour change theory-informed surveys of nursing staff to determine “who needs to change what” to implement inpatient HIRAID®. Focus group interviews will further determine barriers and enablers to implementation. A modifiable implementation strategy will be developed for each cluster. In Phase 3, the finalised framework and implementation strategy will be evaluated with a Type II effectiveness-implementation hybrid design, including a stepped-wedge cluster randomised control trial (SW-cRCT), conducted in 35 wards from 10 hospitals in New South Wales (NSW) and Victoria, Australia.

## Methods: participants, interventions and outcomes

### Phase 1: Delphi study—adaptation of HIRAID® Inpatient

A consensus-driven, real-time Delphi method with expert panel input will be used. Anonymous feedback will be provided during data collection, rather than after each Delphi round, improving efficiency and achieving consensus comparable to conventional Delphi studies [15, 16].

Aim: to adapt the HIRAID® emergency nursing framework for use in the inpatient care setting to assist in the assessment, management and delivery of person-centred and culturally safe inpatient care.

#### Participants

The expert panel will be purposefully recruited, and will consist of frontline hospital inpatient nurses, managers, physicians, surgeons, cultural safety academics, allied health clinicians and consumer investigators, representing those involved in delivering and receiving care. Given the relatively narrow scope and aim of this survey, and assuming a 20% attrition rate, this study will aim to recruit approximately 50 participants.

Participants will be selected from the investigation team (Principal or Co-Investigator) for their expertise in nursing, medicine, pedagogy, education and training, research, cultural safety, clinical governance, behaviour change, and expertise in the HIRAID® nursing framework. Additionally, key stakeholders nominated by hospital executives, such as experienced RNs from study wards, as well as patients and carers, will also be included. Prior to completing the Delphi survey, participants will attend a 30-min virtual education session on HIRAID® Inpatient. This session will summarise the framework and provide an opportunity for roundtable discussion and reflection.

#### Data collection

The Delphi survey will be informed by a review of current policies, education and orientation practices, and documentation templates collected from included inpatient hospital wards. The survey will also draw on the HIRAID® framework, and a Delphi survey previously used to adapt HIRAID® for aged care settings [17]. Roundtable discussions during panel preparation will provide additional input for survey development and an opportunity to develop a pilot version of the HIRAID® Inpatient framework.

Input from the expert panel will be collected using online surveys over three months. The survey will comprise nine sections, a short demographic questionnaire and one section for each of the eight elements of the original HIRAID® framework (Fig. 1). During each Delphi survey round, panel members can review anonymised real-time responses, including vote distributions, comments, and answers to open questions.

The Delphi survey will be hosted on *Calibrum*™ (Calibrum International, USA, calibrum.com), a secure web-based application for data management and survey tools. Each round of the Delphi survey will involve distributing three emails to eligible participants: one initial invitation when the survey opens, one reminder email one week after the initial invitation and one final reminder email two days before the survey closes. Participants will have two weeks from the first invitation to consider participation and then complete and return the survey.

- Round 1: A pilot version of the HIRAID® Inpatient framework will be distributed, along with a series of open questions to prompt experts to identify key areas for framework modification and adaptation. Responses from the first round will be summarised using content analysis and a de-identified report will be redistributed to participants prior to Round 2. Round 1 results will inform the framework and items in the subsequent round.
- Round 2: The second round will present a series of proposed framework changes, and the panel will be asked to rank their agreement for each item on a 5-point Likert scale. The responses will again be summarised and reported back to participants.
- Round 3: Based on the outcomes of Round 2, a third and final survey round will be conducted refining the framework further and confirming panel consensus.

#### Data analysis and outcomes

Data will be exported from *Calibrum*™ into SPSS V28™ (IBM Corp., Armonk, NY, USA) to obtain basic summary and descriptive statistics for demographic and other quantitative data [18]. Descriptive statistics will be used to summarise sample characteristics and outcomes, and further statistical tests will be conducted to ensure the sample is representative of the members of the panel based on demographic characteristics. Qualitative data will be exported to NVivo 14 (Lumivero, Denver, CO, USA) for content analysis, where responses will be analysed by at least two members of the research team and organised into categories and themes [19].

The level of consensus for each item will be calculated using a content validity index (CVI = number of respondents that agree/total respondents). Predefined levels of consensus (high, moderate, low) will be determined prior to the study: high consensus will be defined as ≥ 70% agreement, moderate consensus as 50–69% agreement, and low consensus as < 50% agreement. Items achieving high consensus will be integrated into the HIRAID® Inpatient framework.

### Phase 2: Explanatory sequential mixed methods study to design HIRAID® Inpatient implementation strategy

#### Study design

This explanatory sequential mixed methods study will deploy behaviour change theory-informed surveys of nursing staff to determine “who needs to change what” to implement HIRAID® Inpatient. Surveys will be followed by a series of focus groups with self-nominating nurses to further explore the barriers and enablers to HIRAID® Inpatient implementation. Quantitative and qualitative findings will be integrated and analysed using the Theoretical Domains Framework (TDF) and Behaviour Change Wheel (BCW) to develop an evidence-informed, modifiable implementation strategy for each cluster. This approach has been successfully used in trials based in the ED [13].

#### Participants and eligibility criteria

##### Nursing staff

The nursing workforce includes enrolled nurses (ENs), RNs, Nurse Units Managers (NUMs), Clinical Nurse Consultants (CNCs), Nurse Practitioners and Clinical Nurse Educators (CNEs). Permanent inpatient nurses (full/part time) and casual staff on the study wards will be invited to complete the survey. Agency nurses will be excluded, due to varying degrees of corporate knowledge, organisational governance and procedural familiarity. Email invitations to participate will be distributed by NUMs.

#### Data collection

##### Survey

Four emails will be distributed to eligible participants: an initial invitation when the survey opens, and weekly reminders until the survey closes. Participants will have four weeks from the first invitation to consider participation and to complete the survey. After completing the electronic survey pre-intervention, nurses will be invited to participate in a focus group discussion with a member of the research team to gather insights on their perceptions of the intervention and implementation enablers/barriers to HIRAID® Inpatient.

##### Focus groups

Semi-structured focus groups with consenting nurses will be conducted to deepen understanding of barriers and enablers to HIRAID® Inpatient implementation. This method is commonly used in health research to gain greater insight into complex phenomena [20]. Four to six focus groups (or until data sufficiency is reached) will be completed for each cluster, with an anticipated four to eight participants per group. The focus groups will take place in a private room away from the clinical area to minimise distractions while allowing clinicians to quickly return to the clinical area in case of an emergency. A semi-structured interview guide will consist of predetermined closed and open questions but will also enable the facilitator to ask additional questions that arise during the interviews. Interview questions will include nurses’ assessment and escalation processes, as well as perceptions of communication. Sessions will be audio recorded and transcribed verbatim. A facilitator will take field notes during the session if indicated. Two facilitators will attend each focus group.

#### Statistical methods

##### Survey

Data will be exported from REDcap™ into SPSS V28™ (IBM Corp., Armonk, NY, USA) to obtain basic summary and descriptive statistics for demographic and other quantitative data [18]. Descriptive statistics will be used to summarise sample characteristics and outcomes, and further statistical tests will be considered to ensure the sample is representative of the members of the panel based on demographic characteristics.

##### Focus groups

Qualitative data from free text survey comments and focus groups will be exported to NVivo for content analysis, where responses will be analysed by at least two members of the research team. Focus groups will be transcribed verbatim and original recordings permanently destroyed once the transcription is complete. Transcripts will be analysed and organised thematically and stored and managed using NVivo 14 (Lumivero, Denver, CO, USA) [19]. Thematic analysis will be guided by Gibbs’s framework [21] which includes: 1) transcription and familiarisation; 2) code building; 3) dis/confirmatory theme development; and 4) data consolidation and interpretation. The analysis framework provides a systematic approach to interpretation [22, 23]. All data sources will be triangulated to identify confirmatory and dis-confirmatory findings relevant to the patient safety climate. Triangulation of data will assist in the interpretation of findings.

##### Integration

The quantitative and qualitative data will be analysed and synthesised to identify consistent patterns and discrepancies. Categorical questions with a response rate of 70% or higher will be classified based on their wording; negatively worded questions will be labelled barriers and positively worded questions will be labelled enablers [24, 25]. The results will be integrated to derive the final barriers and enablers to HIRAID® Inpatient implementation. Barriers decrease the chance of the desired behaviour being performed while an enabler increases the chance of the desired behaviour being performed [25]. Qualitative responses will be categorised and used to confirm or refute the barriers and enablers identified in the quantitative data.

##### Implementation study design

Once integrated, the new findings will be mapped to the TDF to enable a comprehensive, targeted theory-informed approach to the development of the implementation strategy [26]. The TDF is used to identify determinants of behaviour and provide comprehensive and systematic approaches to intervention implementation [26]. The mapped TDF domains will be integrated into the BCW, through linking to intervention function(s). The BCW is a validated series of tools for developing behaviour change interventions and is shown to improve the sustained success of clinical interventions [27]. Intervention functions form part of the BCW and are designed to influence behaviour by addressing capability, opportunity, and motivation [27]. If items cross multiple TDF domains and/or intervention functions, the research team will resolve this through a discussion, led by a senior member of the research team with extensive experience in the use of the TDF and behaviour change theories (lead author). The final implementation strategy will be refined using behaviour change techniques taxonomy and the APEASE criteria (Acceptability, Practicability, Effectiveness, Affordability, Spill-over effects, Equity) [28].

### Phase 3: Implementation evaluation of HIRAID® Inpatient

#### Study design

In Phase 3, the HIRAID® Inpatient framework will be implemented using a Type II effectiveness-implementation hybrid design [29], including a SW-cRCT [30]. The hybrid approach allows testing of the implementation strategy at the same time as observing the outcomes of the intervention [29, 30]. Four clusters will be progressed through three sequential phases: control, intervention transition and post-intervention, with a staggered rollout and a 1:1 allocation ratio, with most data collected from each defined population as outlined in Table 4. The trial is structured within a superiority framework, aiming to demonstrate improved outcomes compared to usual care.

**Fig. 3:**
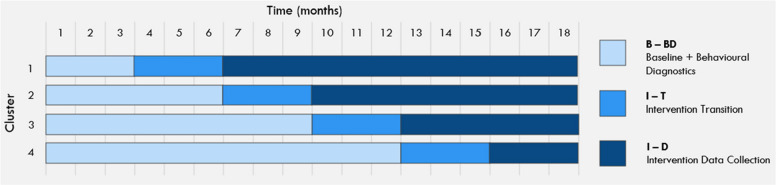
The stepped-wedge cluster randomised controlled trial design for the implementation of HIRAID® Inpatient across four clusters. Each sequence comprises a 3- to 12-month baseline control condition, a 3-month intervention transition period, and a 3- to 12-month intervention exposure condition

Each participating hospital ward (cluster) will begin in the control phase, representing a period of routine or usual care before the scheduled implementation of the HIRAID® Inpatient framework. During this phase, baseline data and behavioural diagnostics will be collected to serve as the comparator for the intervention. Rather than using a separate, concurrent control group, this SW-cRCT uses each cluster as its own control. Data gathered during the pre-intervention phase will be directly compared with data collected post-implementation. This design enables evaluation of the intervention against established, real-world clinical practice. The HIRAID® Inpatient intervention will then be implemented over a three-month period (Intervention Transition), followed by an intervention data collection period, lasting between 3 and 12 months, depending on the cluster (Fig. 3).

##### Hypotheses

The evaluation of the intervention will test the seven hypotheses listed in Table 1.

**Table 1:** List of the seven primary hypotheses to be tested throughout the implementation and evaluation of HIRAID® Inpatient

Hypothesis	Implementation of HIRAID® Inpatient will result in a…
H_1_	…20% reduction in rapid response team activation due to preventable patient deterioration
H_2_	…10% reduction in Hospital Acquired Complications (HAC03 and HAC06)
H_3_	…20% reduction in events causing patient harm or near miss
H_4_	…10% improvement in experience of care reported by patient or carer
H_5_	…10% increase in quality of nurse communication and handover
H_6_	…healthcare resource use and cost estimates that will lead to favourable cost-effectiveness estimates
H_7_	…high fidelity (80%) implementation

#### Study setting

To ensure geographically and clinically diverse settings, the study will take place in three local health districts in two states, comprising a total of 10 hospitals and 35 wards (total 834 beds) from Illawarra Shoalhaven and Western Sydney Local Health Districts in New South Wales, and Eastern Health in Victoria. The ten hospitals will be nested into one of four clusters based on geography and clinical governance to mitigate the risk of contamination. Collectively the study wards manage 4050 patient admissions per month and employ 1259 nurses (Table 2). These hospitals were selected as they admit acutely unwell patients directly from the ED—the patients most at risk for adverse events [4].

**Table 2:** Profiles of the ten participating hospitals, including description, hospital acquired infections (per 1000), the annual number of admissions, and ward type

Cluster	Health Service data for 2023		Study ward data for 2023	
	Description	HAI:1000	Admits per year	Ward type
1. Illawarra Shoalhaven North, NSW (Wollongong hospital—6 wards)	Regional: 684 km^2^, Pop: 214,000 +	3.22	11,400	Aged Care, Gastroenterology, General Medicine, Renal, Respiratory, Surgical
2. Illawarra Shoalhaven South, NSW (Shellharbour Hospital—4 wards; Shoalhaven Hospital—4 wards; Milton/Ulladulla Hospital—1 ward)	Rural Regional: 4708 km^2^, Pop: 185,000 +	2.34	12,861	Aged Care, General Medicine, Surgical
3. Western Sydney, NSW (Westmead Hospital—5 wards; Blacktown/Mount Druitt Hospital—7 wards; Auburn Hospital—1 ward)	Metro: 780 km^2^, Pop: 946,000 +	2.35	13,367	Aged Care, Orthopaedics, Cardiology, General Medicine, Haematology, Neurosurgery, Renal, Respiratory, Stroke
4. Eastern Health, VIC (Box Hill Hospital—4 wards; Angliss Hospital—2 wards; Maroondah Hospital—2 wards)	Metro: 2816 km^2^, Pop: 750,000 +	3.33	10,981	General Medicine, Speciality Medicine, Stroke, Surgical (general, plastic surgery and gastric surgery)
10 hospitals	Pop: 2.1 million +		48,609	

#### Participants and eligibility criteria

##### Nursing staff

All permanent and fixed-term employee nursing staff will complete HIRAID® Inpatient training and be invited to participate in the evaluation of the intervention (i.e. surveys on quality of information in handovers and confidence in patient assessment and escalation of care).

##### Medical and allied healthcare staff

Medical and allied healthcare staff employed or contracted by partnering organisations will be eligible to participate in the evaluation of the intervention with no exclusions. Participation will include the completion of staff experience surveys [31], to understand how the intervention improved the quality of nursing communication and handover. Healthcare staff that meet the inclusion criteria will be invited to participate by email invitation for the survey.

##### Inpatients (or their carers) of a study ward

All patients admitted to the ward that meet study inclusion criteria, to evaluate experience, will be provided with a participant information sheet for the study and invited to participate in surveys. Patients are excluded if they are receiving end-of-life care or have moderate–severe cognitive impairment as identified by the treating clinical team.

If under 18, their carer (if over 18) will be approached; otherwise, they will not be invited. We are not targeting paediatric wards; however, there may be outlying paediatric patients admitted to adult inpatient wards.

If the patient is not clinically suitable to approach, (e.g. in significant pain, cognitive impairment), an appropriate carer may be approached. A waiver of consent has been approved for the collection of de-identified inpatient data, which will include medical records, care plans, charts, progress notes, and all associated documentation pertaining to clinical deterioration, incidents, hospital transfers and clinical costings.

##### Inpatient facilitators

All implementation officers and nurse facilitators will be included to evaluate implementation fidelity, as they will be responsible for the implementation of HIRAID® Inpatient.

#### Randomisation

Each cluster will be randomly allocated to one of four starting dates for implementation. Randomisation will be performed by the study statistician using computer-generated numbers. Participating wards will have no influence over the allocation prior to randomisation, ensuring concealment of the allocation sequence until wards are assigned. No further changes to clusters will be made once the allocation has been finalised to minimise the risk of bias.

We are unable to randomise sites to clusters because of geographical location and clinical governance. The significant geographical distance between sites, mitigates the risk of contamination due to reduced proximity. Randomising clusters based on health service provides consistent clinical governance for study operations.

#### Blinding

Blinding investigators and staff delivering the intervention to cluster allocation is impractical. However, researchers assessing nurse-associated clinical deterioration, hospital-acquired complications, and nursing documentation will remain blinded, as well as participants not directly involved in delivering or receiving the intervention (i.e. inpatients, family of inpatients).

#### Study Plan

##### Integration of HIRAID® Inpatient framework into hospital systems

The local electronic and paper-based documentation systems will integrate the HIRAID® Inpatient documentation templates. HIRAID® Inpatient will include a standardised template with fields and prompts for all framework elements. Hospital local factors, such as paper or electronic documentation will inform adaptation practices. Features like the product(s) used (e.g. PowerChart, Cerner electronic medical records (eMR)), automation features, and compatibility with existing tools are included. The documentation templates are designed to streamline assessment documentation, minimise duplication, alleviate administrative burdens, and reduce redundancies for hospital ward nurses. The HIRAID® Inpatient framework will be implemented alongside existing standard nursing care. No concurrent interventions are prohibited during the implementation period; however, any new initiatives or changes to routine care that may influence study outcomes will be documented.

##### Education and training of nursing staff

All nursing staff will participate in education sessions designed to teach the theory and application of HIRAID® Inpatient, using a pedagogically informed approach [32]. The education programme will be co-developed with experts from the participating organisations and will incorporate principles of constructive alignment [33–35], backwards design [36] and scaffolded learning [37] to ensure coherence between learning outcomes, resources and activities.

The education programme will consist of two different training sessions, the instructor and the provider training. The HIRAID® Inpatient Instructor training will be designed to equip healthcare professionals with the skills to teach and implement the framework, and focuses on developing teaching strategies, and applying HIRAID® to a clinical setting. CNCs/CNEs and senior nurses at each site will receive HIRAID® Inpatient Instructor training and will lead delivery of HIRAID® Inpatient Provider courses for all RNs and ENs from participating wards using a train-the-trainer model. Senior RNs will undergo Instructor training to independently deliver HIRAID® Inpatient training, supporting long-term sustainability. The HIRAID® Provider training will focus on equipping clinicians with the skills to use the framework. Completion of the HIRAID® Inpatient Provider Course will be mandatory for new nursing staff and will be embedded into orientation and onboarding programmes through the established instructor network.

##### Supporting materials

**Fig. 4:**
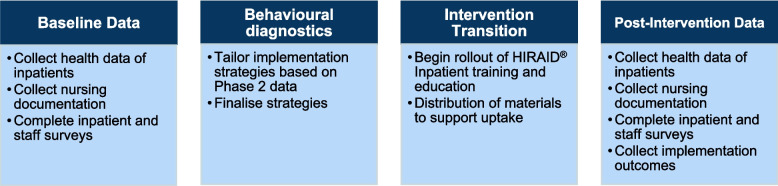
The overall process for the implementation and outcome measurement of the HIRAID® Inpatient intervention

**Fig. 5:**
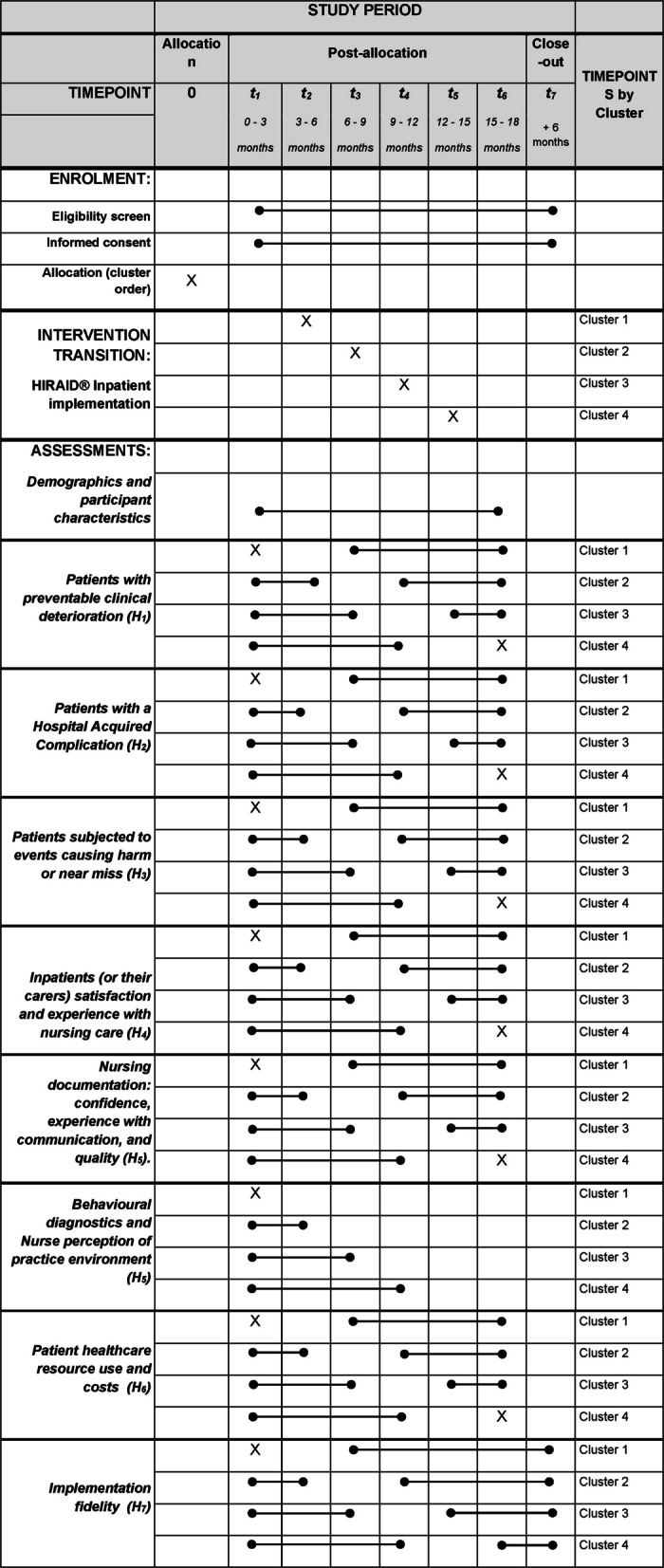
The schedule of enrolment, intervention, and assessments for HIRAID® Inpatient: Implementation evaluation of an evidence-informed hospital inpatient nursing framework (adapted from [59])

Multiple strategies, developed during Phase 2, will be implemented to address hospital-specific barriers and support the rollout. Co-designed educational resources will ensure accessible learning and ongoing support. Environmental prompts, such as posters and reference cards, will serve as quick reminders of the HIRAID® Inpatient framework and its components. Implementation research nurses, along with nurse champions from the workforce, will play a key role in encouraging adoption. These nurses will perform fortnightly audits of nursing documentation to track progress, document the implementation strategies used within their assigned cluster, provide updates, identify teaching opportunities, and address any challenges that arise during implementation. See Figs. 4 and 5 for the overall process of HIRAID® Inpatient implementation and outcome measurement (Phase 3).

#### Outcomes

##### Patients with a preventable clinical deterioration (H_1_)

Rate per 1000 admissions of preventable patient deterioration calls (RRT calls) related to nursing assessment and management, observation and monitoring. RRT activation is already recorded in local databases and will be used to identify patients for medical record review, independent assessment and verification of causal factors [38]. Secondary outcome is transfer to a higher level of care (e.g. to Intensive Care Unit) (Table 3).

**Table 3:** Summary of HIRAID® Inpatient outcome measure, data collection sources and measurement timepoints

Outcome	Outcome description	Outcome measure	Data analysis	Data source	Control (pre)	Implementation	Intervention (post)
Patient health outcomes							
Outcome 1 (H1)	Nursing-related preventable clinical deterioration	Incidence of clinical deterioration events related to nursing assessment and care	*Descriptive approach, generalised linear model (GLM) approach	Patient deterioration database, eMR records	✓	-	✓
Outcome 2 (H1)	Transfer to higher level care	Incidence of patients transferred to higher level care	*Descriptive approach, GLM approach	Patient deterioration database, eMR records	✓	-	✓
Outcome 3 (H2)	Hospital Acquired Complications (HACs) influenced by nursing care	Incidence of patient days with respiratory complication (HAC03), and/or healthcare-associated infections (HAC06)	*Descriptive approach, GLM approach	Health District data collections, Performance Planning Unit (PPU) database, eMR records	✓	-	✓
Outcome 4 (H2)	HACs influenced by nursing care–health service perspective	Incidence of hospital resource use and costs	*Descriptive approach, GLM approach	Health District data collections, PPU database, eMR records	✓	-	✓
Outcome 5 (H2)	HACs influenced by nursing care	Rate of all other HACs (not HAC03 and HAC06)	*Descriptive approach, GLM approach	Health District data collections, PPU database, eMR records	✓	-	✓
Outcome 6 (H3)	Events that cause patient harm or near miss	Incidence/proportion of adverse patient harm or near miss events	*Descriptive approach, GLM approach	Health District data collections, PPU database, eMR records	✓	-	✓
Patient and carer experience outcomes							
Outcome 7 (H4)	Patient and carer perceptions and experience of nursing care	Average score reported on the Australian Hospital Patient experience question set and Schmidt's Perceptions of Nursing Care Survey	*Descriptive approach, logistic regression for binary outcome variables, linear regression for continuous outcome variables	eMR records, patient/carer satisfaction surveys, patient complaints	✓	-	✓
Outcome 8 (H4)	Patient and carer overall care experience and patient complaints relating to nursing care	Average scare reported on remainder Schmidt’s Perceptions of Nursing Care Survey, and content analysis of complaints	*Descriptive approach, logistic regression for binary outcome variables, linear regression for continuous outcome variables	eMR records, patient/carer satisfaction surveys, Patient complaints	✓	-	✓
Staff experience and capability outcomes							
Outcome 9 (H5)	Staff satisfaction with communication (handover)	Average score on Staff Satisfaction with Handover Surveys (Inter-professional communication)	*Descriptive approach, multiple linear regression modelling, logistic regression for binary outcome variables, linear regression for continuous outcome variables	Nursing/allied health/medical staff surveys	✓	-	✓
Outcome 10 (H5)	Nurse confidence in patient assessment and escalation of care	Average score on Confidence with communication survey	*Descriptive approach, multiple linear regression modelling	Nursing/allied health/medical staff surveys	✓	-	✓
Outcome 11 (H5)	Quality and quantity of nursing documentation	Average score of modified D-catch tool	*Descriptive approach, multiple linear regression modelling	Nursing/allied health/medical staff surveys	✓	-	✓
System-level and Implementation outcomes							
Outcome 12 (H6)	Acute treatment costs	Average of incremental net health service cost per rapid response team calls avoided	*Descriptive approach, Extrapolation modelling, time horizon modelling, Discrete event simulation (stochastic)	Health District data collections, PPU(Costs) database	✓	-	✓
Outcome 13 (H7)	Implementation fidelity	Outcomes reported on the implementation fidelity surveys and nurse interviews	*Descriptive approach, process evaluation, structured fidelity scoring	Audits, Nursing staff interviews, Nurse facilitator survey	-	✓	✓

*Descriptive approach—means and standard deviation for continuous variables, and frequencies and percentages for categorical variables. Cluster comparisons—ANOVA for continuous variables; Pearson’s Chi-Square tests for categorical variables

##### Patients with a hospital acquired complication (H_2_)

Rate per 1000 patient days of respiratory (HAC03) and healthcare-associated infections (HAC06), the leading preventable HACs influenced by nursing care. Nationally, HACs are reported by facility per 1000 patient days. In NSW, the study sites are above the state average of 2.17:1000 patient days. The secondary outcomes are hospital resource use and costs (health service perspective) and the rate of all HACs.

##### Patients subjected to events causing harm or near miss (H_3_)

Rate of Harm Score 1 per 1000 admissions. Harm scores range from 1 (death) to 4 (near miss) based on the severity of outcome and additional post-incident treatment requirements. In 2023, the study sites had a total of 14 Harm Score 1, 97 Harm Score 2, and 1829 Harm Score 3 events. The secondary outcomes are the rate of harms per 1000 for 2, 3 and 4.

##### Inpatients (or their carers) experience with nursing care (H_4_)

The two validated instruments, the Schmidt’s Perceptions of Nursing Care Survey [39] and the Australian Hospital Patient Experience Question Set (AHPEQS) [40], will be used to measure patient perceptions and experience of nursing care. The resources will be translated into the top five languages for each cluster to enable inclusion of patients who do not speak English [41]. Remuneration will be offered per the NSW Classification and Remuneration Guidelines for NSW Government Boards and Committees ($20 voucher suitable for rural/regional participants) and as a strategy known to increase response rates. The primary outcome is the patient (or their carers’) experience of nursing care. The secondary outcomes include the following: overall care experience; Schmidt’s subscales, including Seeing the Individual Patient, Explaining, Responding, Watching Over; and patient complaints relating to nursing care [39].

##### Nursing documentation (H_5_)

(i) Experiences with the quality of information transfer from nursing staff during handover, and (ii) nurse confidence in patient assessment and escalation of care will be measured through a validated electronic survey of nurses, medical and allied health staff [42]. Clear, coherent and comprehensive communication is essential for patient safety and collaboration [43]. (iii) Documentation quality will be evaluated using the D-catch tool [44] and records checked for sufficiently linguistically correct, legible, and using the correct terminology to meet all medico-legal requirements [45]. A sub-sample of patient records from H_1_ will be used.

##### Patient healthcare resource use and costs data (H_6_)

Inpatient healthcare resource use and costs will be recorded from the administration systems at participating hospitals for usual clinical care activities. In addition, resource use associated with HIRAID® Inpatient implementation will be recorded prospectively. Healthcare resource use will be costed from the perspective of the health service using actual costs or market rates. These data will be used to inform cost-effectiveness analyses.

##### Implementation fidelity (H_7_)

To address H7 (HIRAID® Inpatient is implemented with high fidelity (80%)), CNCs/CNEs employed to assist with education and rollout will be invited to evaluate implementation fidelity. All records of communication kept will be de-identified by the facilitator making the record. A copy of the participant information statement will be provided to all those involved in leading implementation. The HIRAID® implementation evaluation will be guided by the RE-AIM framework [46] and scoring instrument [47]. *Reach* will be measured by the proportion of eligible nurses participating in the programme, *effectiveness* through the end user (nurses’) opinions on the usefulness of HIRAID®, and *adoption* by the proportion of eligible sites implementing HIRAID®. *Dose* is the consistency of exposure to HIRAID® across settings and *fidelity,* the degree to which individual components and the overarching implementation strategy were delivered. *Sustainability* will be determined through demonstration of ongoing HIRAID® training programme use post implementation, along with the proportion of nurses reporting they use HIRAID® at ≥ 6 months.

#### Sample size

Sample sizes were calculated for each of the hypotheses. Hypotheses H1, H2, H3, and H6 sample size estimations were conducted collectively, as they will use medical record and cost data from relevant systems for the period of 1 st July 2026 to 1 st December 2027 (Table 4—shaded grey). To maximise the study’s power in detecting true differences, should they exist, the largest sample size estimation is reported and will be used. Sample sizes were calculated with an assumed intra-class correlation of 3%, 5% significance (two-tailed) and 80% power, and applying Woertman’s SW design estimate approach [48]. These sample sizes have been calculated using the most recent data from the study sites and/or previous work by the investigators in this field. To evaluate implementation fidelity HIRAID® facilitators (CNCs/CNEs) employed to assist with education and roll out will be invited to participate in the evaluation of implementation fidelity. The number of staff to be involved in this process will be determined by each LHD.

**Table 4:**
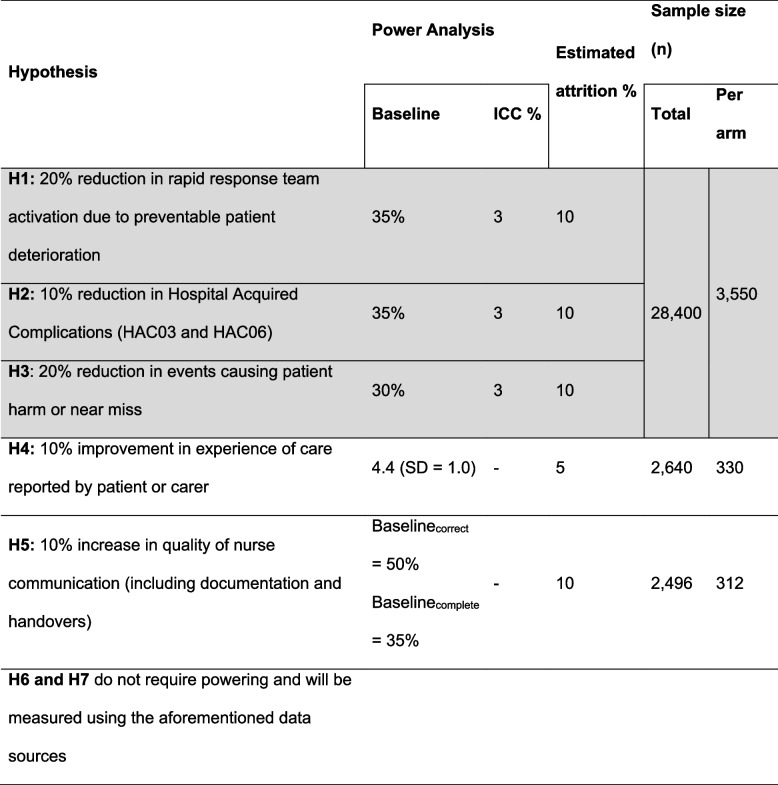
Sample size calculations for seven hypotheses showing the target total and per arm (per data collection period) sample sizes

α =0.05, *β* = 0.20; *ICC* intra-class correlation

#### Data collection

Several outcome measures will be collected from a variety of data sources during the control and intervention periods to evaluate the effectiveness of HIRAID® Inpatient and address the study hypotheses. Data will be collected from the patient health records made available by each ward, from patients, carers of patients, nurses, medical staff and the nurse facilitators who are employed to deliver the HIRAID® Inpatient training. Data collected on patient experience, record reviews, and staff surveys will be collected and directly entered into REDcap™ (Research Electronic Data Capture) (https://catalyst.harvard.edu/services/redcap/), a secure web-based application for data management and survey tool, managed by the University of Sydney. The type of data that will be collected from each of these sources, and when they will be collected, are summarised below and in Table 5.

**Table 5:** Summary of the data to be collected from each population at pre- and post-intervention, and the instrument that will be used

Population	Pre-intervention (baseline)	Post-intervention (outcomes)	Instrument
Nurses	Behavioural diagnostics		Theoretical domain framework, focus groups
		HIRAID® Inpatient evaluation and feedback	Feedback survey
	Satisfaction with communication and handover		Satisfaction with communication survey
	Perception of factors that influence nursing care		Practice Environment Scale of the Nursing Work Index
	Quality and quantity of nursing documentation		Modified D-Catch Tool
	Confidence in communication		Confidence with communication survey
Patients/Carers	Satisfaction with nursing care		Schmidt's Perceptions of Nursing Care Survey
	Experience		Australian Hospital Patient Experience Question Set
Medical and Allied Health staff	Satisfaction with communication and handover		Satisfaction with communication survey
Patients	Health data		Patient health records
	Satisfaction with nursing care		Schmidt's Perceptions of Nursing Care Survey
	Experience		Australian Hospital Patient Experience Question Set
Nurse facilitators	Implementation fidelity		Implementation fidelity survey

##### Deteriorating patient database and electronic medical record (eMR)

Patient’s that have a rapid response call activated while an inpatient in a study ward will be identified through existing site rapid response databases. In addition to reviewing the quality of documentation using the audit tool (Supplement 1), these records will be reviewed to identify causal factors associated with the rapid response call and determine if suboptimal inpatient nursing care was a precursor, for example, unreported or delayed reporting (> 30 min) of vital sign abnormalities fulfilling hospital criteria. The instrument (Supplement 1) outlines the variables collected and incorporates the validated Human Factors Classification Framework for patient safety [38], which considers equipment, work environment, staff action and patient factors. To ensure consistent data collection, research nurses assessing clinical documentation and patient deterioration events will complete data collection on a minimum sample of 10 duplicate records to complete inter-rater reliability at commencement. Ongoing audits will maintain data collection consistency.

##### Hospital data collections

Reports from existing hospital data collections will be accessed. The principal executive at each cluster has confirmed that these data can be provided. Data will be extracted from the relevant financial unit for each ward pre and post implementation to enable economic evaluation of any cost benefits associated with adverse event rates associated with the implementation of HIRAID® Inpatient framework across the sites.

##### Surveys

Surveys will be distributed electronically to nominated emails for patients/carers, and staff emails to all nursing, medical and allied health staff who meet the inclusion criteria. Participants who complete the pre- and post-intervention surveys will be offered a range of $20 vouchers, in accordance with the NSW Classification and Remuneration Guidelines for NSW Government Boards and Committees and as a strategy known to increase response rates.

##### Patient carer experience surveys

To assess patient experience, two instruments will be used (Supplement 2). The AHPEQS [49] is a validated survey that has been developed with input from Australian consumers [40]. The AHPEQS consists of 12 questions related to patient experience. The validated Schmidt’s Perceptions of Nursing Care Survey [50] will be used to measure patient perceptions of nursing care. The survey consists of 15 items on a 5-point Likert scale from strongly disagree (1) to strongly agree (5).

On the day of discharge or within two weeks of discharge from the hospital ward research nurses will make a follow-up call (Supplement 3) to complete the survey. If the time is not convenient, they will arrange an alternate time and day for survey completion as soon as practical. While the likelihood of the patient being deceased is low, as part of the preliminary phone call to carers, the well-being of the patient will be ascertained using sensitive and considerate language (outlined in the interview script). The research nurse will also access the patient’s medical record to ascertain if the patient has been readmitted since discharge and is an inpatient again, or deceased. If deceased, they will not call. The same approach will be used if any subsequent calls are required to complete the survey (Supplement 3).

##### Nursing staff surveys

For the nursing staff, a survey will be distributed to collect the following:

1. Nurse characteristics (control and intervention). A short survey will collect data on nurses’ position, experience and expertise. Gender will not be collected, as we do not plan to target male, female, or non-binary staff any differently in our implementation strategy [51].
2. Nurse confidence (control and intervention). Confidence in patient assessment and communication will be measured using a validated survey for use in the Australian setting [31]. The scale consists of 14 items and nurses indicate their level of confidence for each item on an 11-point Likert scale ranging from no confidence (0) to complete confidence.
3. Nurse experience with communication (control and intervention). We will measure nurse experience with registered nurse (RN) communication and quality of information received during handover, using a previously validated instrument [31]. This instrument consists of 8 items on a 11-point Likert scale, five yes/no questions and a free text box for written responses.
4. Nurse perception of practice environment (control only). We will measure nurses’ perception of the practice environment using the validated *Practice Environment Scale of the Nursing Work Index (PES-NWI).* This will determine if the proposed implementation, use and effect of HIRAID® Inpatient varies due to organisational and other factors that influence a nurse’s ability to practice skilfully and deliver high quality care. The PES-NWI consists of 28 items and nurses indicate their agreement on a 4-point Likert scale ranging from strongly disagree (1) to strongly agree (4).
5. HIRAID® Inpatient feedback and evaluation (intervention only). This brief survey will measure nurses’ overall perception of HIRAID® Inpatient, its effectiveness and the implementation process developed in Phase 2 of the study and used in the rollout of the framework across the local health district (Phase 3). As such, the exact number of items is not yet known. Responses will follow a yes/no format or a 5-point Likert scale measuring frequency (1—never to 5—always), with opportunity to provide free text explanations. Findings will inform a future toolkit, focusing on implementation strategies, organisational support, uptake of protocols, utility and sustainability.

Data from these surveys will have multiple purposes. Responses to the practice environment (PES-NWI) and behavioural diagnostic instruments (control only) will inform the implementation strategies of the HIRAID® Inpatient intervention at the respective ward. The final feedback and evaluation instrument will collect nursing staff feedback on intervention implementation, assessing rollout strategies to inform the development of a future toolkit.

##### Medical and allied health staff survey

1. Medical and allied health staff satisfaction with nurse communication (control and intervention). Staff satisfaction with RN communication and quality of information received during handover, using a previously validated eight-item, 11-point Likert scale, plus five yes/no questions instrument [31]. The opinion of permanently employed medical and allied health staff is sought as a component of HIRAID® Inpatient communication. A unique identifier will be generated by responders to enable post-intervention matching. The survey will be distributed electronically and used to inform H5 (improved quality of nursing communication and handover).

##### Implementation fidelity

Prior to measuring the effectiveness of the intervention on patient and health services outcomes, the implementation strategy requires evaluation. This evaluation will focus on implementation fidelity, where the extent to which an intervention is delivered per the intended implementation plan is measured [52, 53]. Evaluation of our behaviour-change-informed strategy will be developed using the RE-AIM Scoring Instrument and measured using data from (i) the post HIRAID® implementation nurse survey, (ii) HIRAID® Facilitator surveys, and (iii) documentation audits.

1. HIRAID® Inpatient Facilitator survey. Facilitators will independently complete intervention fidelity scoring, that asks to what degree the behaviour change mechanisms were implemented [53, 54]. Implementation scores range from 0 (“not implemented”) to 2 (“fully implemented”). Adaptions for each mechanism will be scored from 1 (“just as planned”) to 4 (“many changes from plan”) [55].

#### Statistical methods

##### HIRAID® effectiveness/clinical outcomes

Survey and health record data collected for implementation evaluation will be analysed using descriptive statistics to assess fidelity levels and feedback from study sites. Descriptive statistics will be calculated using the chi-square test for categorical variables, and the *T*-test (normally distributed data) or the Mann-Whitney *U* test (non-normally distributed data) for continuous variables. All analyses will follow the intention-to-treat principle, with all clusters evaluated according to their original group allocation, regardless of adherence or protocol deviations. Comparisons within and across clusters will be conducted using the generalised linear mixed model (GLMM) approach, applying appropriate link functions tailored to each outcome variable. For binary outcome measures, a GLMM with a binary logistic link function will be used, while for count outcome measures, GLMMs with Poisson or negative binomial link functions will be applied. The model will account for the random effect of the cluster and will adjust for relevant confounders, identified in the descriptive analyses, as fixed effects. SPSS V28™ (IBM Corp., Armonk, NY, USA) will be used and the alpha set to < 0.05 [18].

##### Implementation fidelity audits

Data will be analysed using descriptive statistics to determine the level of implementation fidelity. This process of evaluation will be conducted to assess for fidelity and success of implementation across sites [56, 57]. Fidelity controls will be deployed for internal and external validity of implementation and measured through audits and implementation logs.

##### Handling of missing data

Missing data will be addressed using appropriate imputation methods, informed by the pattern of missingness (e.g. whether data are missing at random or not). Sensitivity analyses will be conducted to assess the robustness of findings, including complete-case analysis and the use of alternative imputation strategies if patterns of missingness suggest potential bias. Additional analyses per protocol may also be applied to further examine the robustness of results.

#### Data management

##### Data storage and access

Data will be managed in accordance with NSW Health research data management policy and procedures. All data will be kept for a minimum of 15 years post-publication, in accordance with the 2018 National Health and Medical Research Council (NHMRC) Australian Code for the responsible conduct of research and accompanying guides [58].

##### Data de-identification and linkage

Research nurses at the study site will have access to eMR data, used to identify patients that meet study inclusion criteria and determine those patients that require medical record review and/or rapid response review. Only site researchers will have access to their identifiable site data. Once data collection is complete, all data will be imported into Excel and then integrated using the identifiers of MRN, name and age. These three identifiers are required to maximise the likelihood of correctly matching patients between datasets.

### Ethics and dissemination

#### Data monitoring

A Data Monitoring Committee will not be required for this trial due to the absence of significant blinding and the relatively low-risk nature of the intervention. Trial Steering and Investigator Committees will be established to oversee the study which includes the chief investigator, site leads, and consumer representatives. Any decision to modify or terminate the trial will be made collaboratively by the Steering and Investigator Committees in consultation with the trial sponsor and ethics committee. The Chief Investigator, supported by a project manager and site staff will manage daily operations including recruitment, data handling, stakeholder engagement and reporting. The project statistician will monitor data to ensure consistency. Data entered by research officers into REDCap™ will undergo automated plausibility checks (i.e. acceptable value range, detection of invalid characters) and regular audits. The University of Sydney will be the coordinating centre.

#### Harms

While participants are not expected to experience adverse effects from direct involvement in the study, provisions are in place to address any emotional distress that may arise. Should a participant become emotionally distressed during survey or focus group participation, the activity will be ceased immediately. Support will be offered including referral to their General Practitioner or appropriate support services such as Beyond Blue, Lifeline, or 13YARN. The research nurse will assist in arranging support if needed.

Adverse events will be managed according to site procedures and reported in line with NHMRC Guidance on Safety Monitoring and Reporting guidelines and the University of Sydney’s Safety Reporting Guidelines 2021. The chief investigator, along with site investigators, will collect, oversee documentation and reporting. Serious adverse events will be reported to the University of Sydney Clinical Trial Support Office, the relevant hospital governance office and escalated to the Trial Steering Committee for review and management.

#### Dissemination

Irrespective of the outcome, the findings will be shared through various platforms, including open-access peer-reviewed journals, conference presentations, and local, national, and international forums. Authorship for publications will be determined prior to submission and will adhere to the International Committee of Medical Journal Editors’ guidelines for peer-reviewed works [59]. Anticipating intervention efficacy, the results will be communicated to relevant national agencies and stakeholders to influence nursing practice, health policy, and nurse education.

## Discussion

HIRAID® Inpatient aims to optimise nurses’ contribution to the quality and safety of hospital care nursing care by preventing adverse events, improving patient experiences and strengthening clinical communication through a standardised, evidence-informed framework for assessment, decision-making and action. Study strengths include methodological design, theoretical foundations, and implementation strategies that will be underpinned by evidence derived from the development, refinement and implementation of HIRAID® in the ED [7] and aged care settings [32]. Hospital inpatient wards provide care that is often long-term, with a focus on ongoing treatment plans and recovery. This differs from the ED, where care is provided rapidly in a fast-paced environment, and aged care facilities where the focus of care is on managing enduring health conditions and supporting activities of daily living. However, all care settings share commonalities as they are complex, resource-constrained environments that require person-centred care, clear communication and adherence to standards and policies. The experience and insights gained from implementing HIRAID® in other settings will provide a foundation for approaches to increase the likelihood of successful and sustained implementation.

A diverse sample of specialty wards within hospitals of different delineations, including rural, regional and metropolitan hospitals, will be engaged in this study to maximise the generalisability of findings and provide insights relating to the site-specific needs, adaptations and considerations. The incremental implementation of the stepped-wedge design is well-suited to the evaluation of health service outcomes and practical to implement [30]. By each cluster contributing data to both control and intervention phases, the risk associated with comparing heterogeneous hospitals is mitigated. Additionally, geographical separation minimises the risk of contamination between clusters/sites.

### Methodological considerations and risks

The study requires substantial commitment from healthcare partners, including the co-design, implementation, maintenance, and evaluation of HIRAID® Inpatient. Following success in the ED, nursing management advocated for HIRAID® adoption in the hospital inpatient setting. However, input and engagement from frontline nurses must be considered. In addition to Phase 2 surveys and focus groups, an assessment of current documentation, education and orientation practices in specialty wards will be conducted to engage nursing staff and address inherent challenges unique to these diverse patient care settings.

Due to the nature of the intervention, blinding will be limited. While recipients will be blinded to cluster order, they cannot be blinded to the transition from control to intervention conditions, introducing a risk of bias in self-report measures (e.g. nurses’ perceived confidence). For non-self-reporting measures (e.g. clinical deterioration events), objective assessment and repeated measurements across baseline and post-intervention periods mitigate bias. To minimise bias, assess long-term outcomes and detect relapse effects, most outcome measures will be collected for a range of 3–12 months post-intervention.

The application of HIRAID® to a ward context poses a delivery risk. To manage this, pre-implementation diagnostics will identify potential issues, and solutions will be incorporated into the site-specific implementation plans. Implementation research nurses will escalate and manage challenges through a hospital working group and research management group.

### Trial status

All ethical and governance approvals are in place at participating hospitals. The protocol is Version 5.0 dated 30th April 2025. Recruitment is scheduled to begin on the first of July 2026 and will be completed approximately on 31 st December 2027. The trial is prospectively registered with the Australia New Zealand Clinical Trial Registry ACTRN12625000639426 (registration date: 17/06/2025).

## Conclusion

Nurse-led frameworks improve patient outcomes; however, there is a paucity of standardised evidence-informed nursing frameworks used in the hospital inpatient setting. This contributes to increased preventable adverse events in Australian hospitals, leading to unintentional harm, death and healthcare costs. Based on previous application of HIRAID®, it is anticipated that the HIRAID® in the inpatient setting will provide a nursing framework that will be person-centred, fit-for-purpose and facilitate the assessment of the entire patient. Empowered with this tool, nurses will be able to deliver high-quality care and be better equipped to make sound and evidence-informed decisions.

## Supplementary Information

Supplementary Material 1.

Supplementary Material 2.

## Data Availability

Not applicable.

## Abbreviations

AHPEQS: Australian Hospital Patient Experience Question Set
BCW: Behaviour Change Wheel
CNC: Clinical Nurse Consultant
CNE: Clinical Nurse Educator
ED: Emergency department
eMR: Electronic medical record
EN: Enrolled nurse
HACs: Hospital-acquired complications
HIRAID®: History including Infection risk, Red flags, Assessment, Interventions, Diagnostics, communication and reassessment
HREC: Human Research Ethics Committee
ISLHD: Illawarra Shoalhaven Local Health District
NHMRC: National Health and Medical Research Council
NSW: New South Wales
NUM: Nurse Unit Manager
PES-NWI: Practice Environment Scale of the Nursing Work Index
RE-AIM: Reach, Effectiveness, Adoption, Implementation (dose/fidelity) and Maintenance/Sustainability
RN: Registered nurse
RRT: Rapid Response Team
SW-cRCT: Stepped-wedge cluster randomised control trial
TDF: Theoretical Domains Framework
WSLHD: Western Sydney Local Health District
