# Supplementary figures and images for: Implementation evaluation of an evidence-informed hospital inpatient nursing framework (HIRAID® Inpatient): a protocol for a stepped-wedge cluster RCT
Source: Trials. 2025 Dec 4;27:22. doi: 10.1186/s13063-025-09313-8 (PMC12798057; doi:10.1186/s13063-025-09313-8)

## Supplement 1


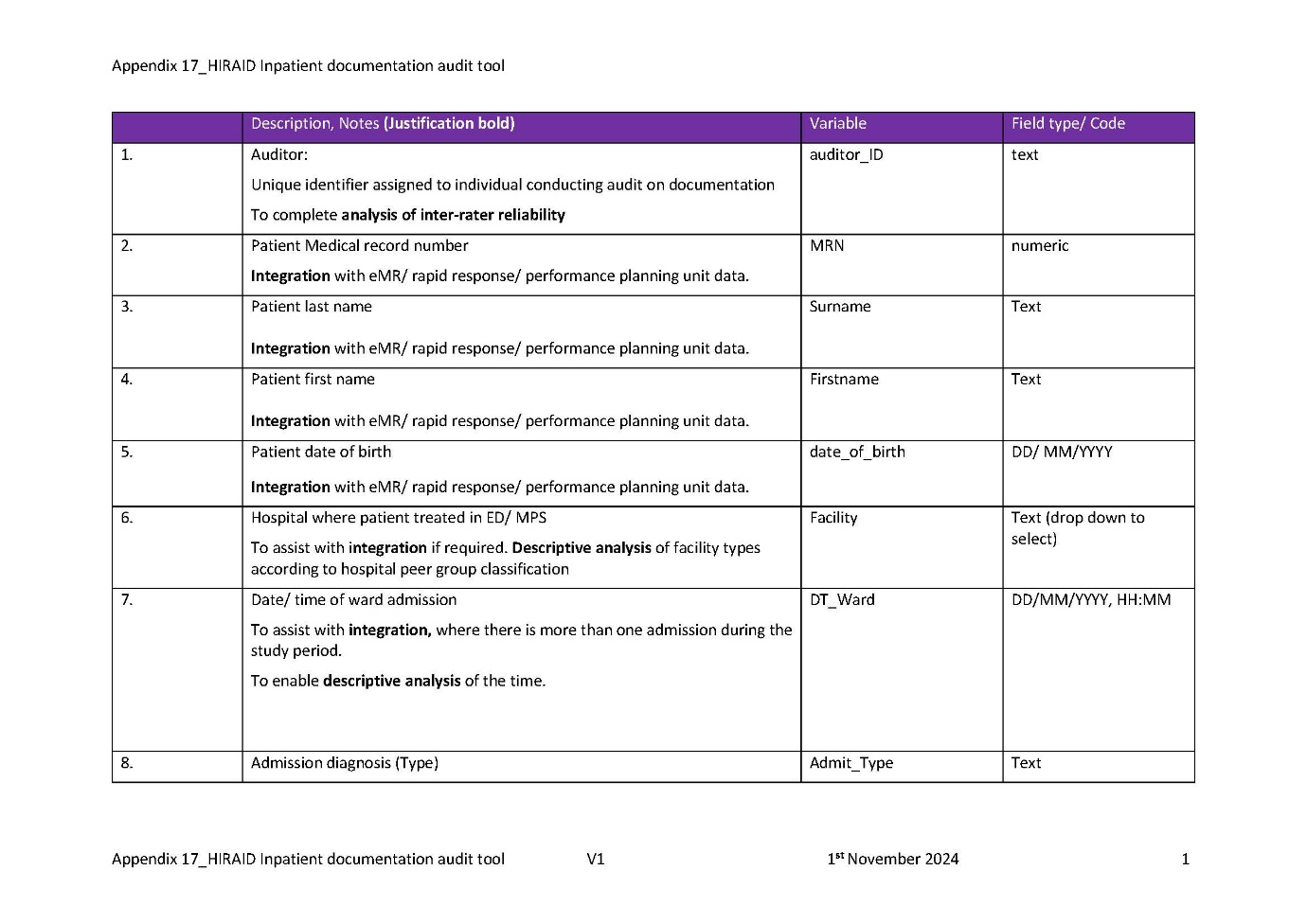


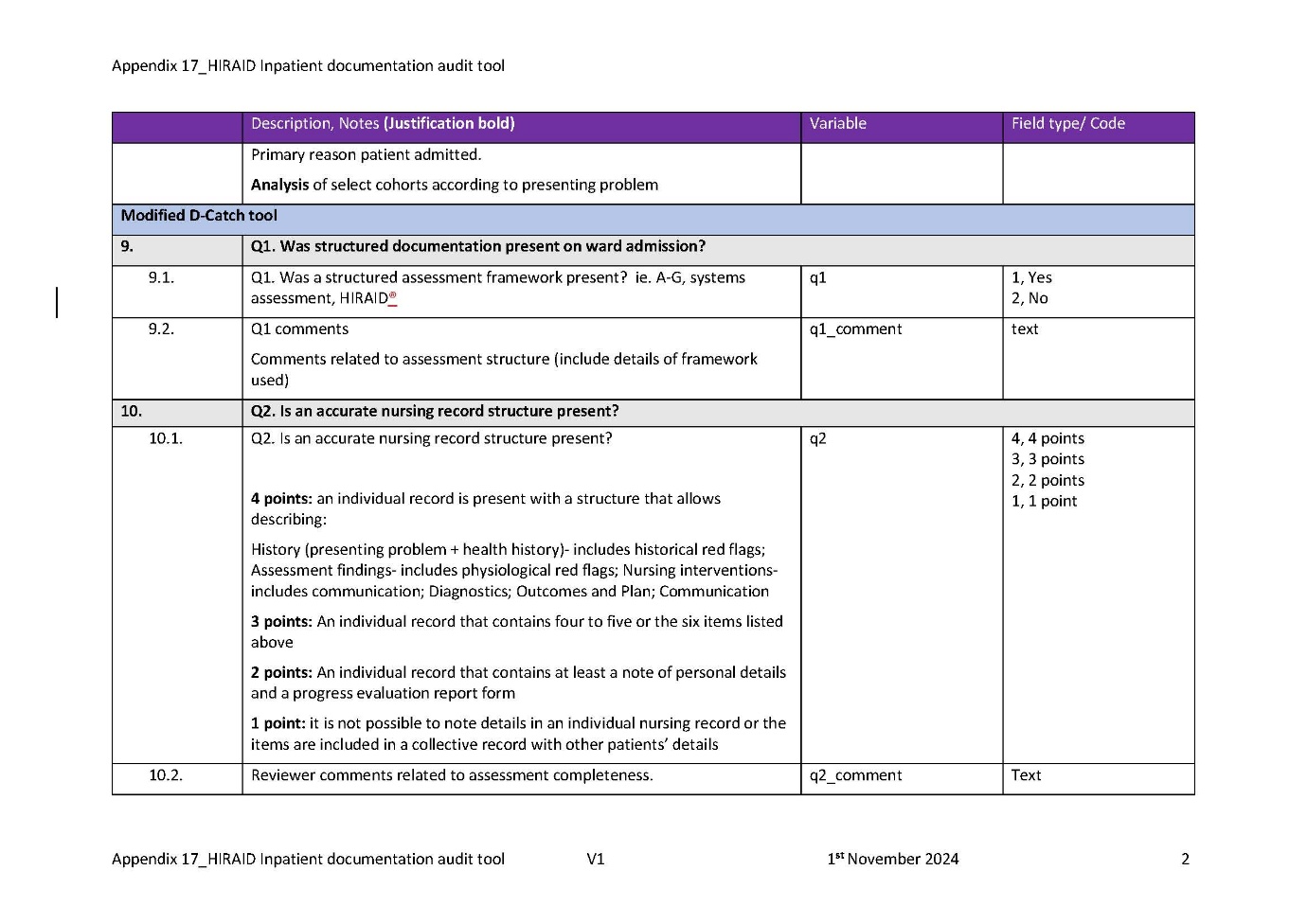


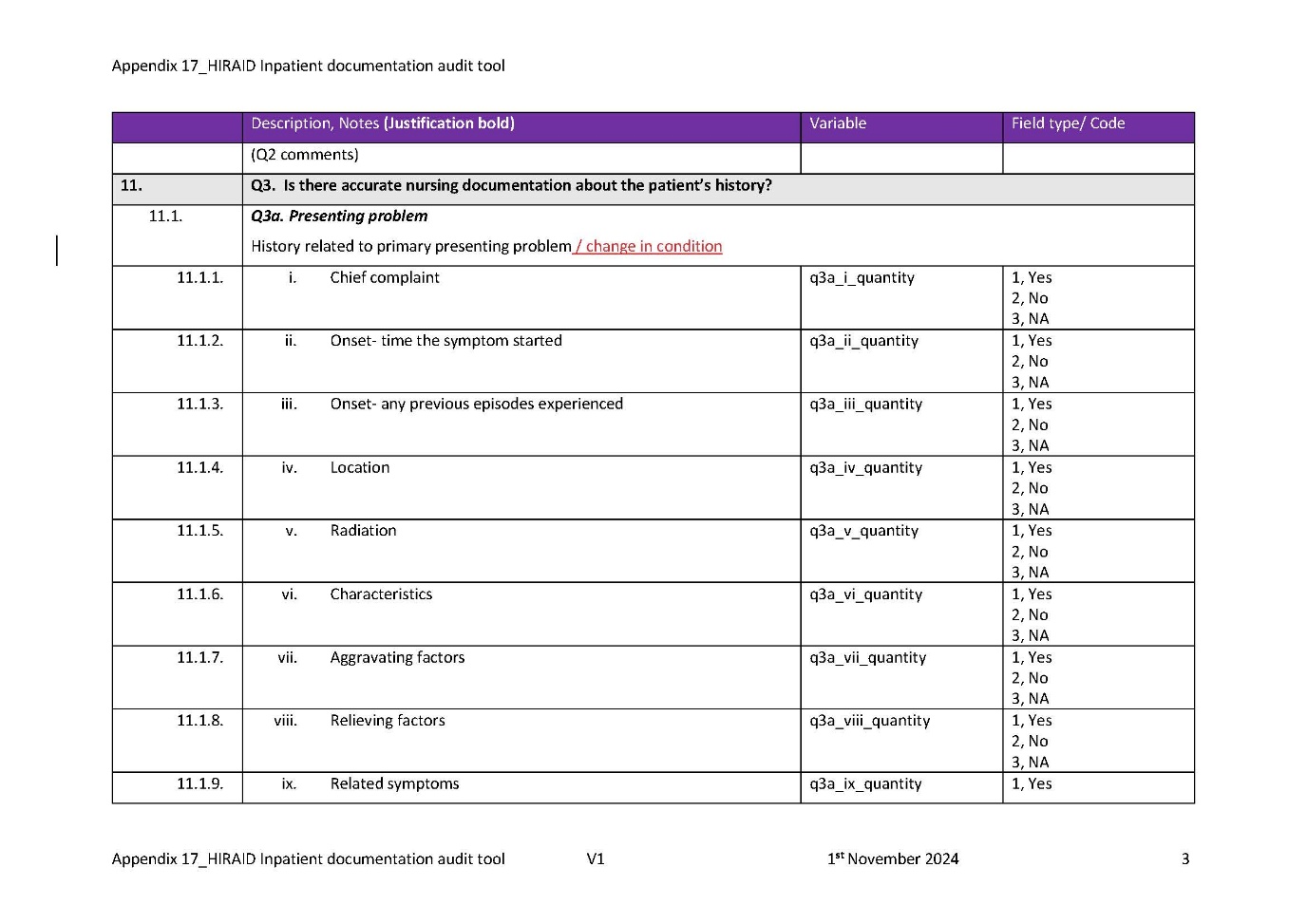


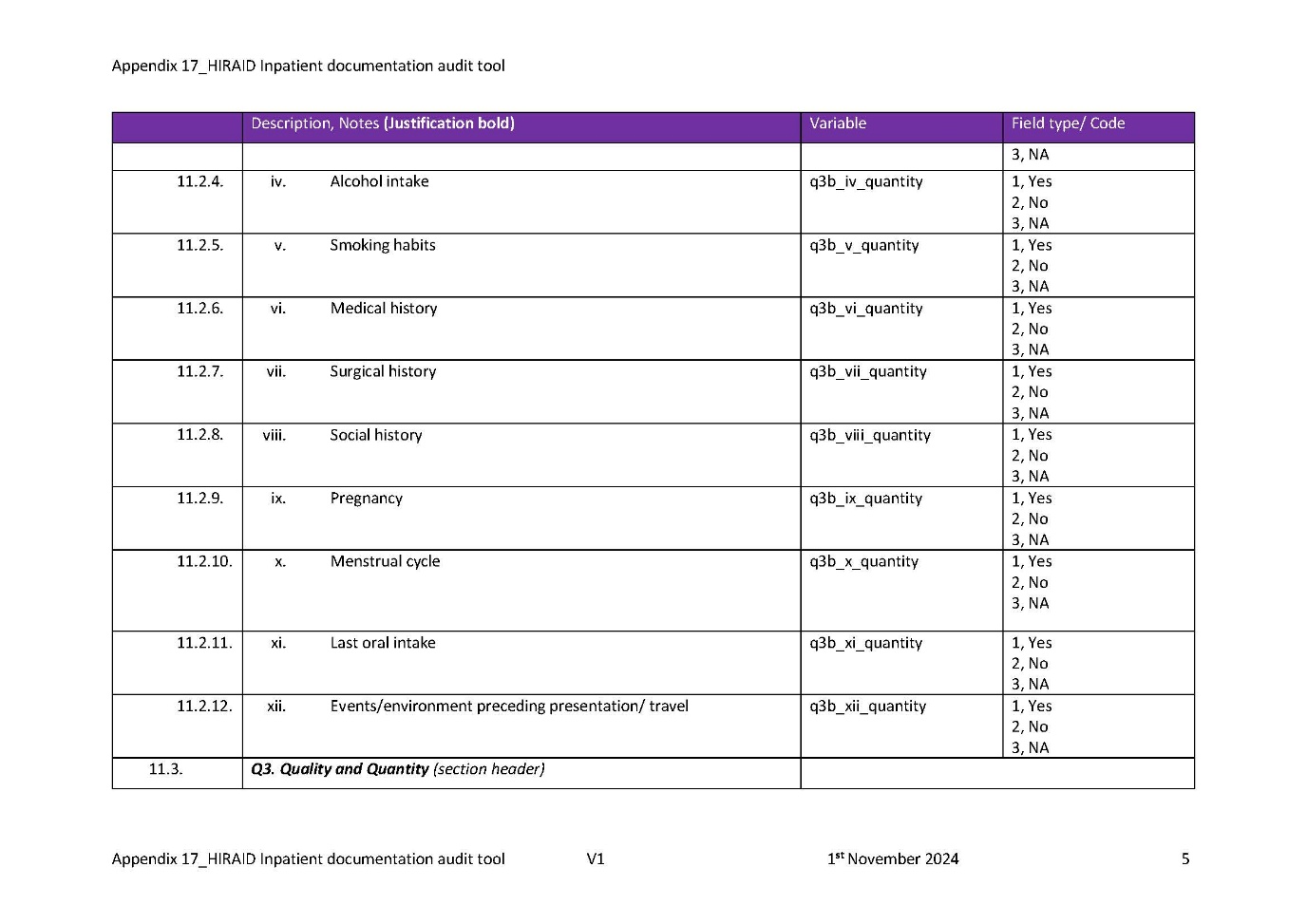


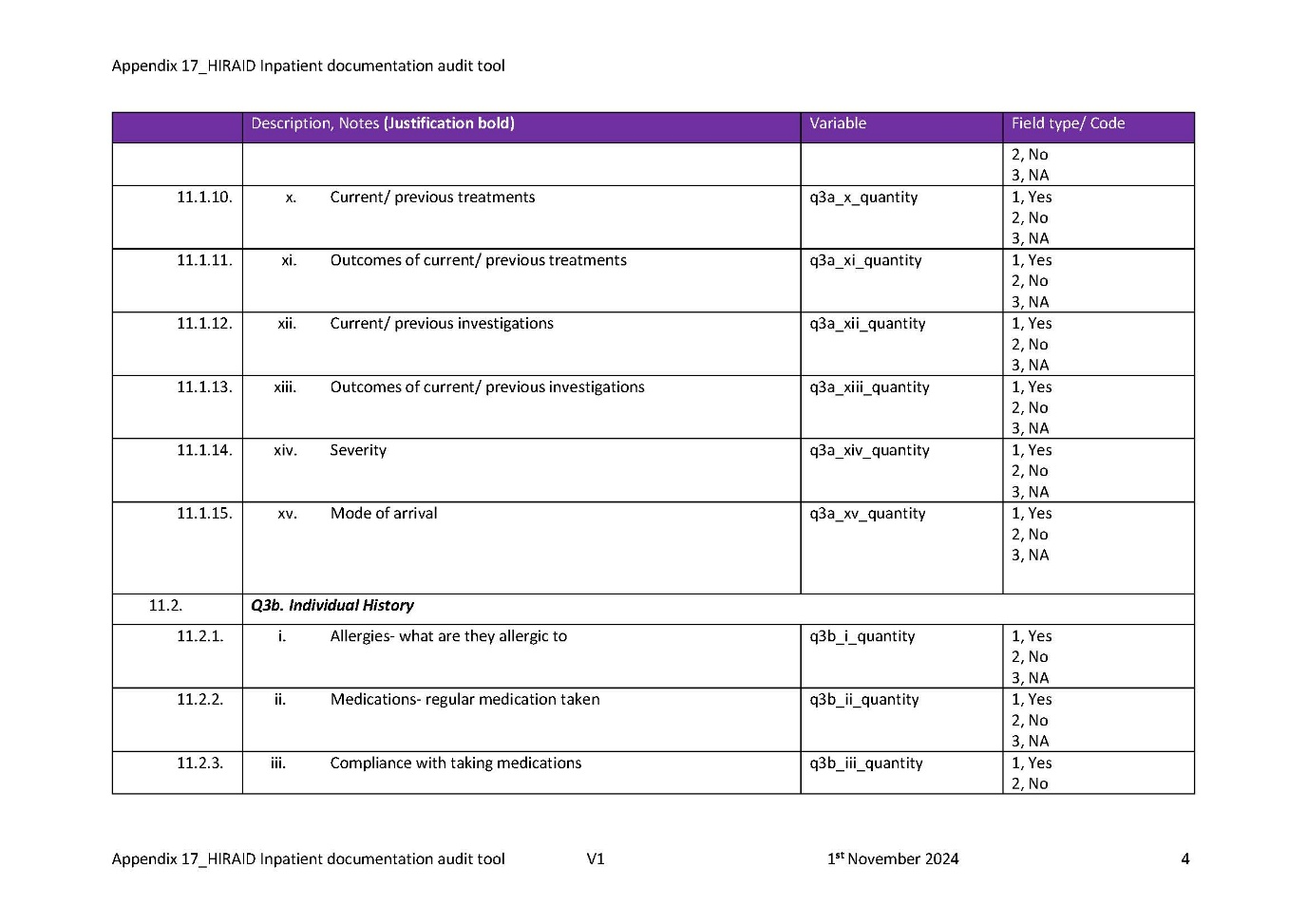


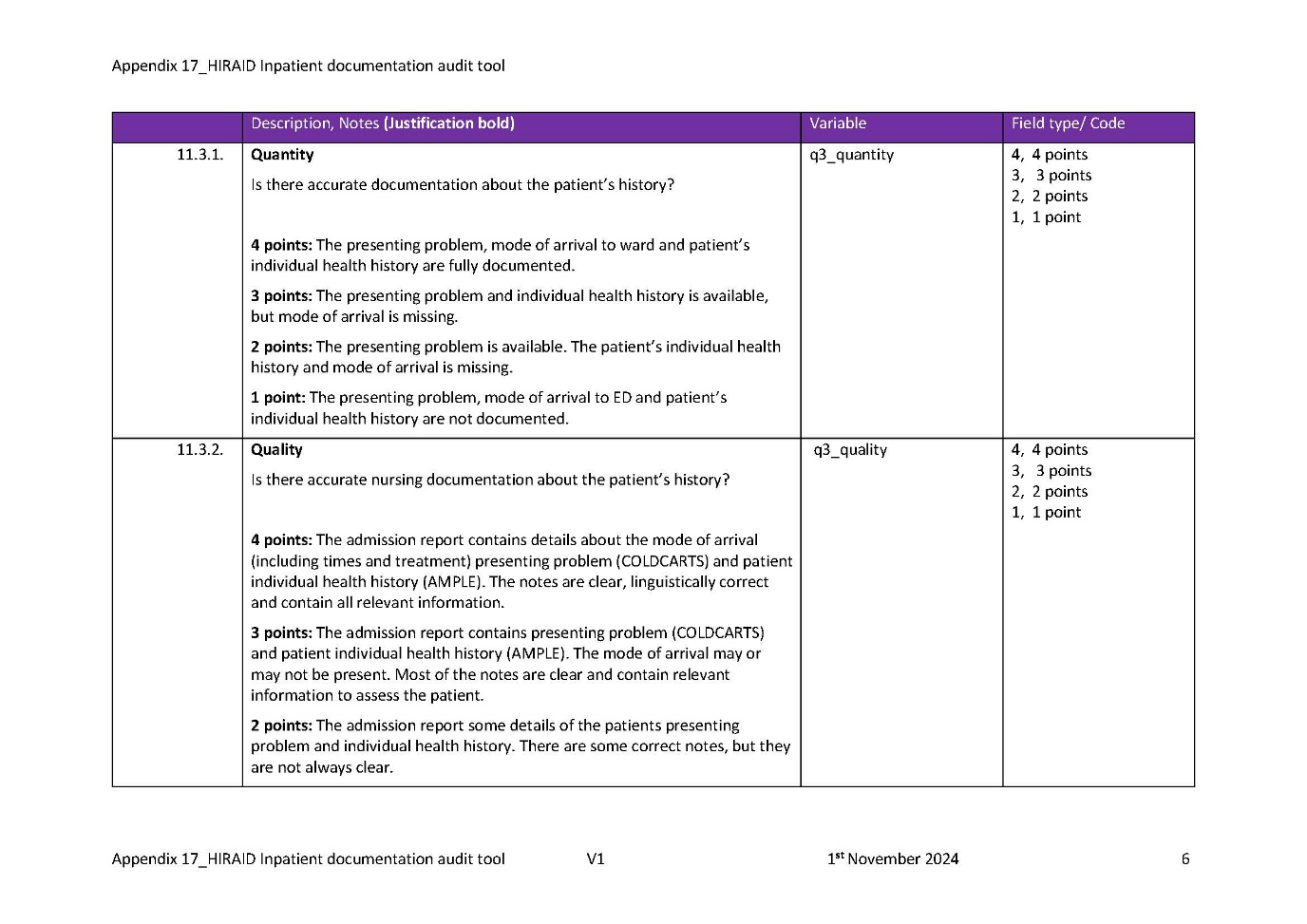


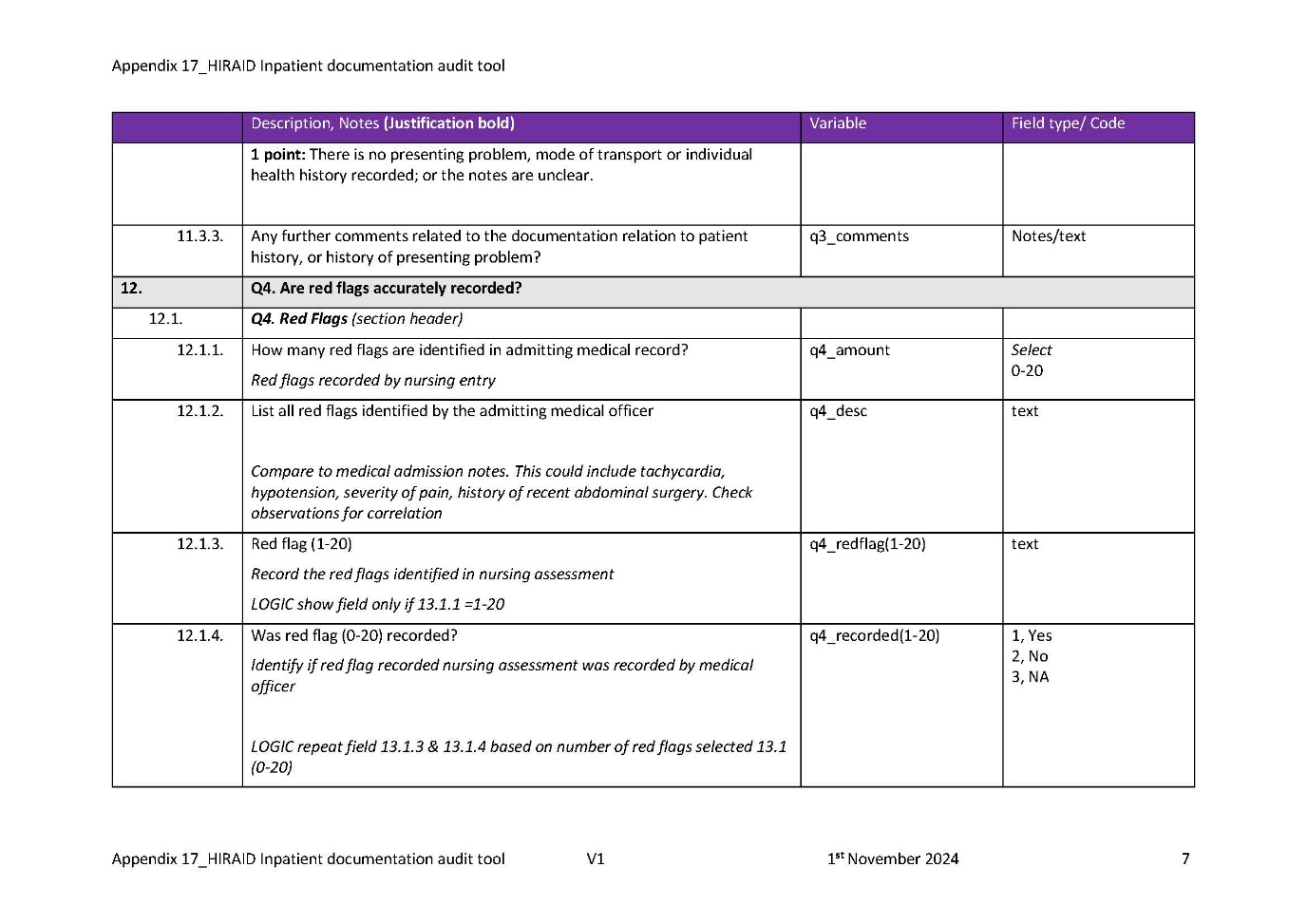


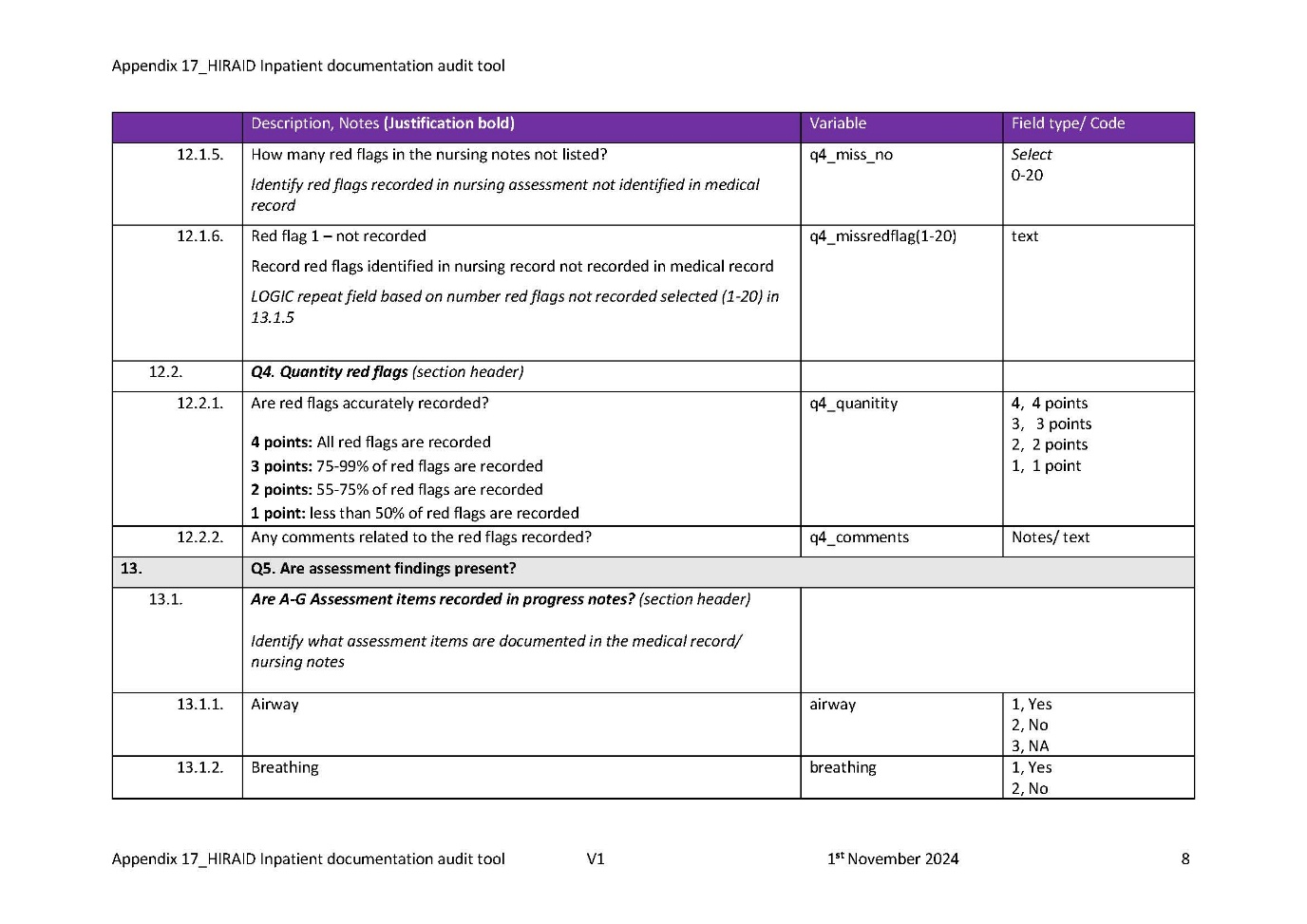


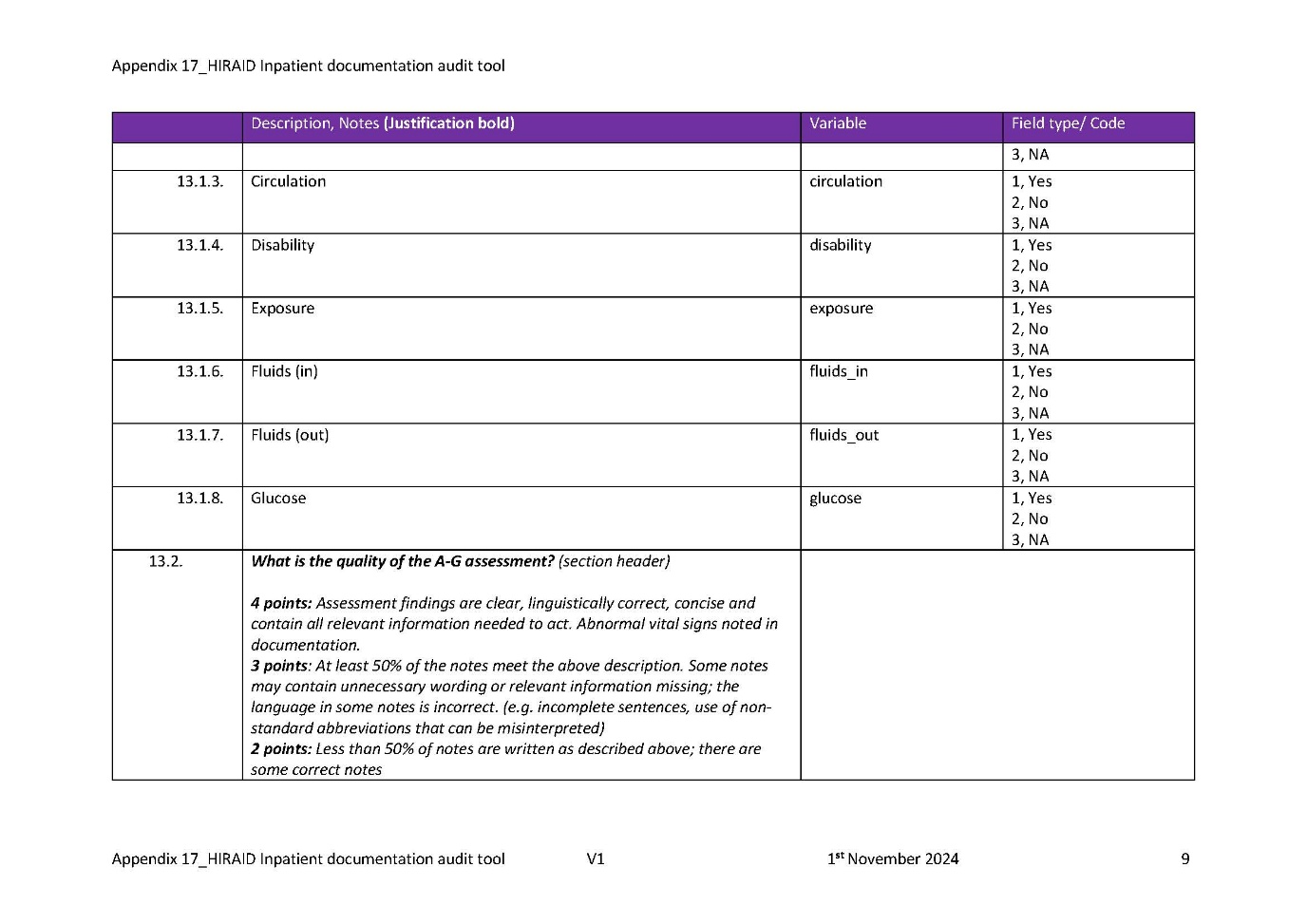


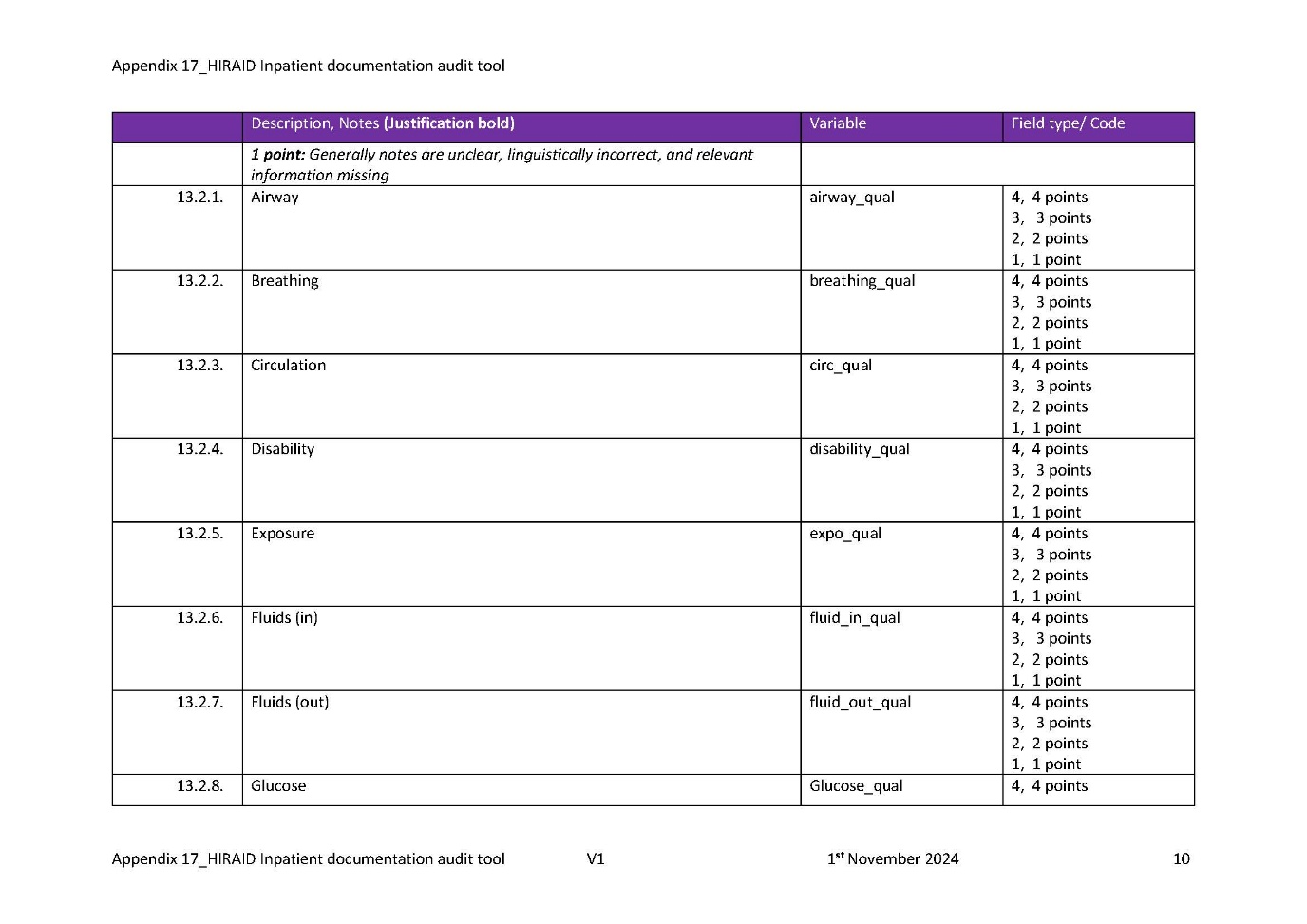


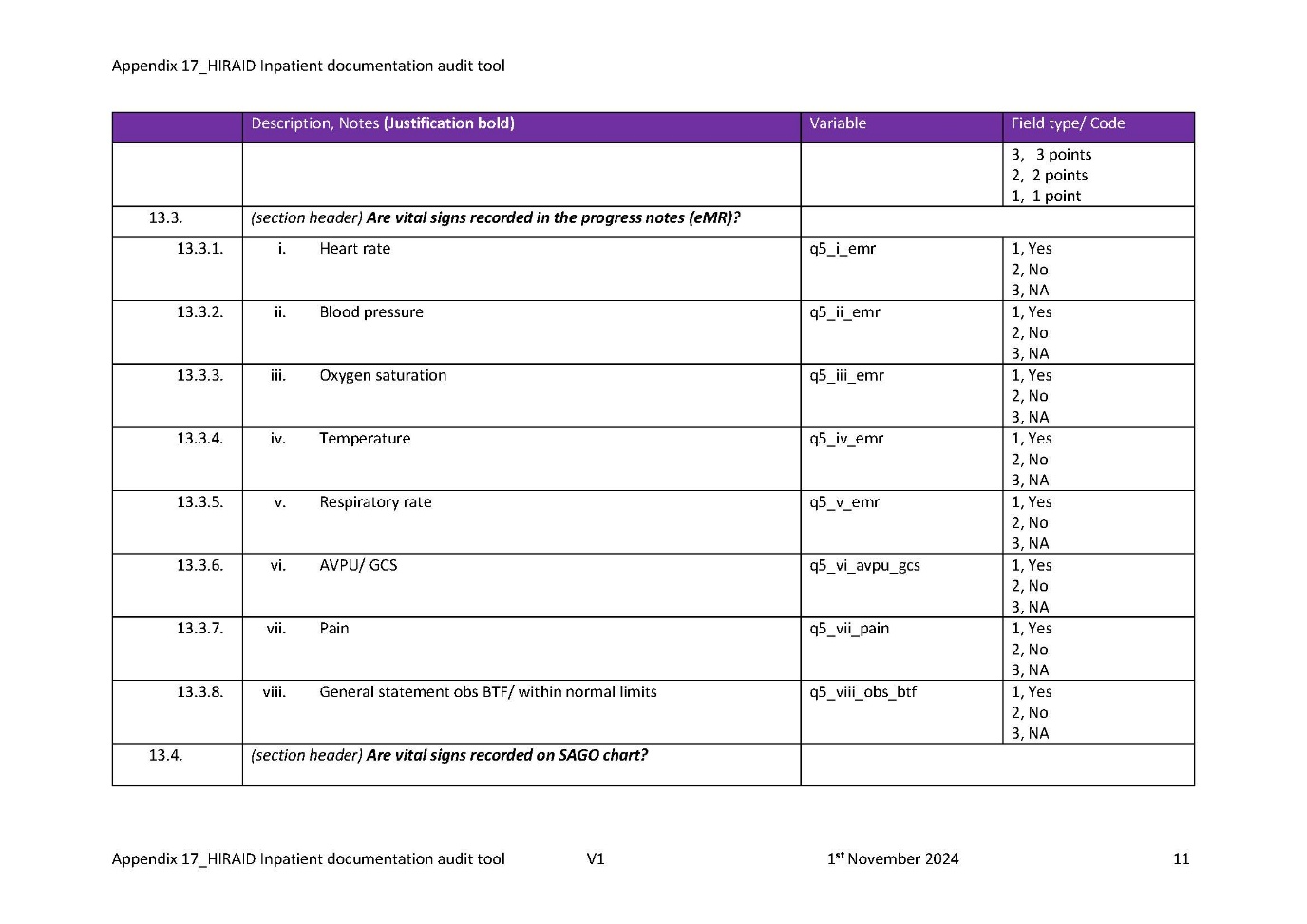


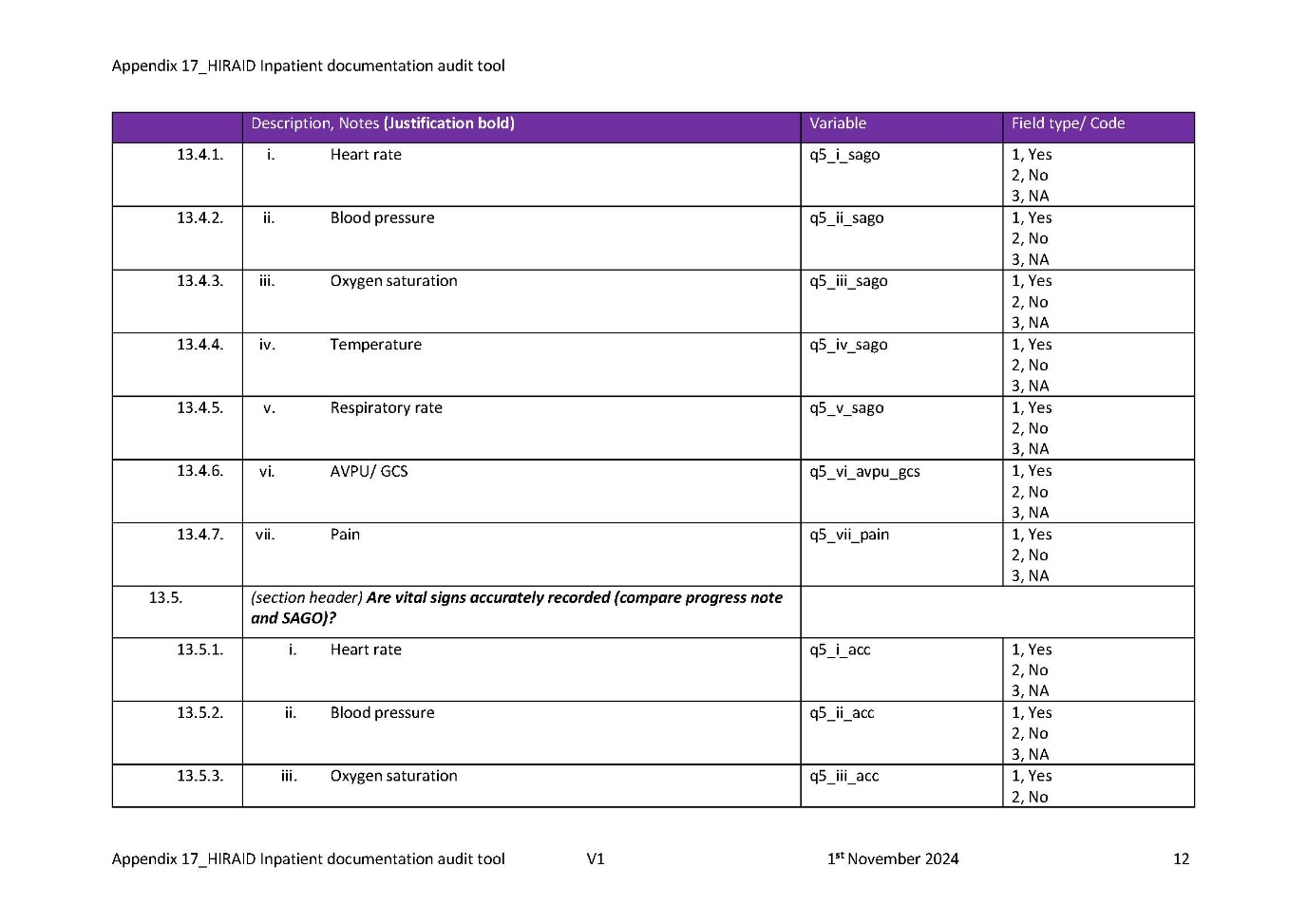


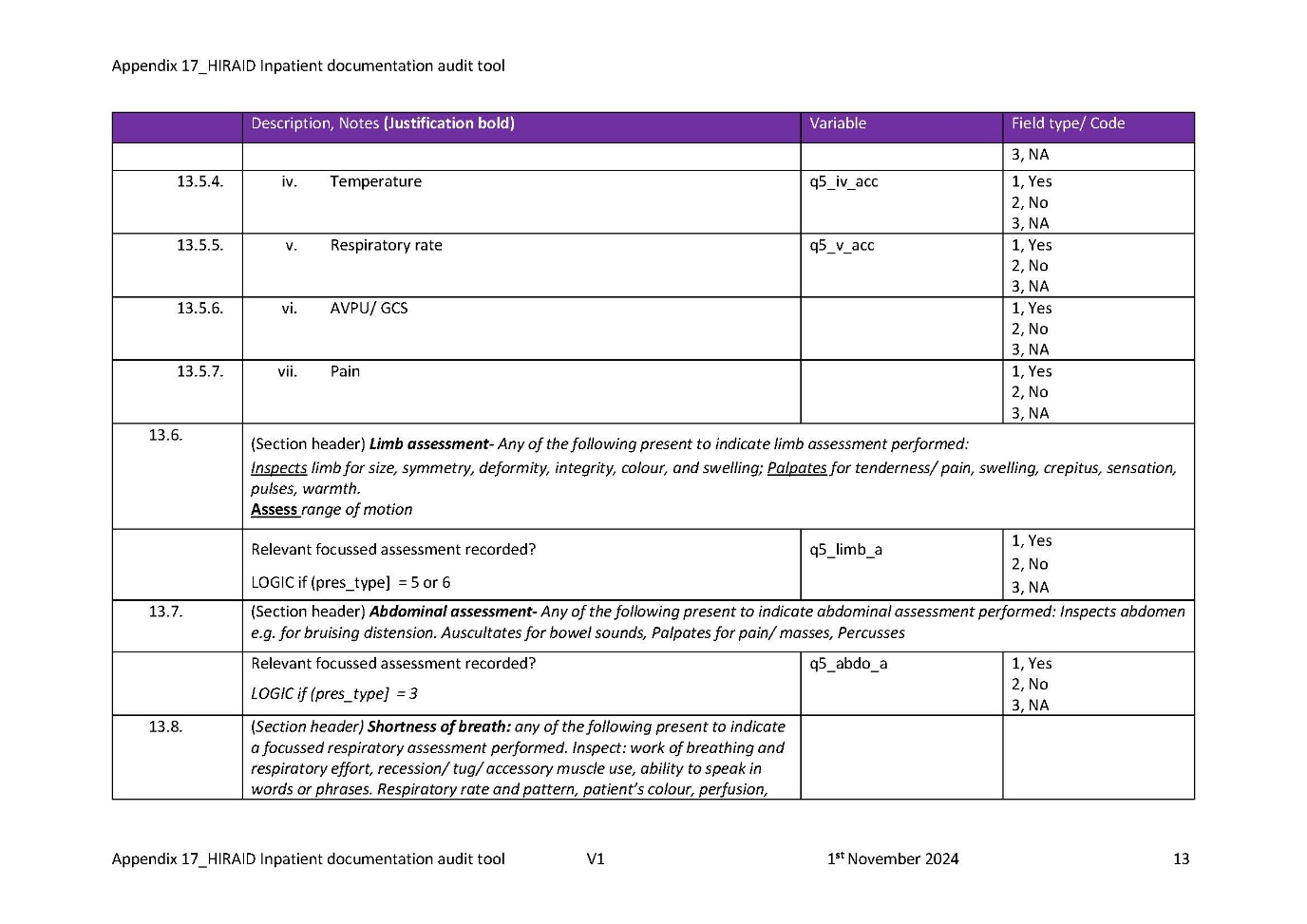


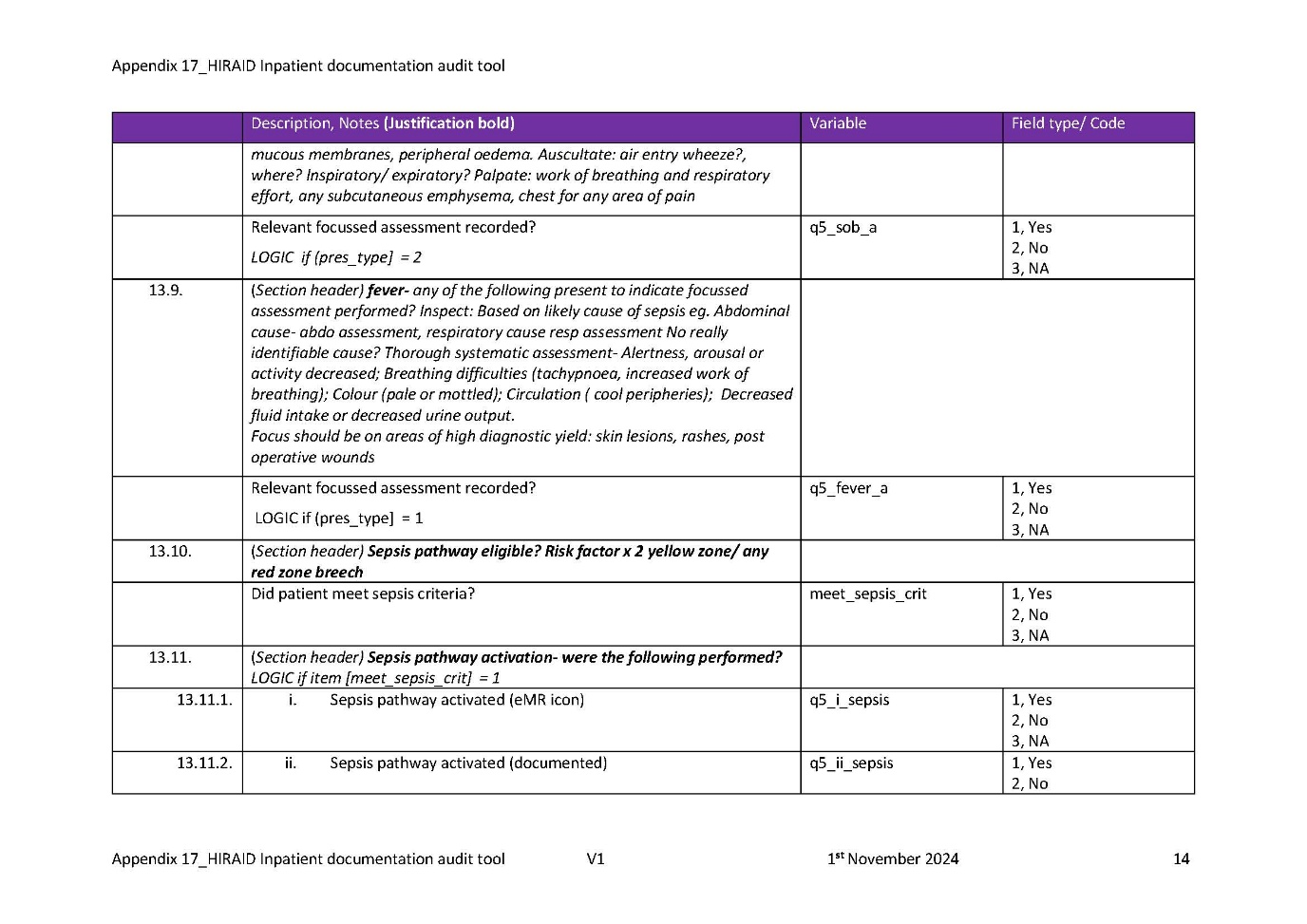

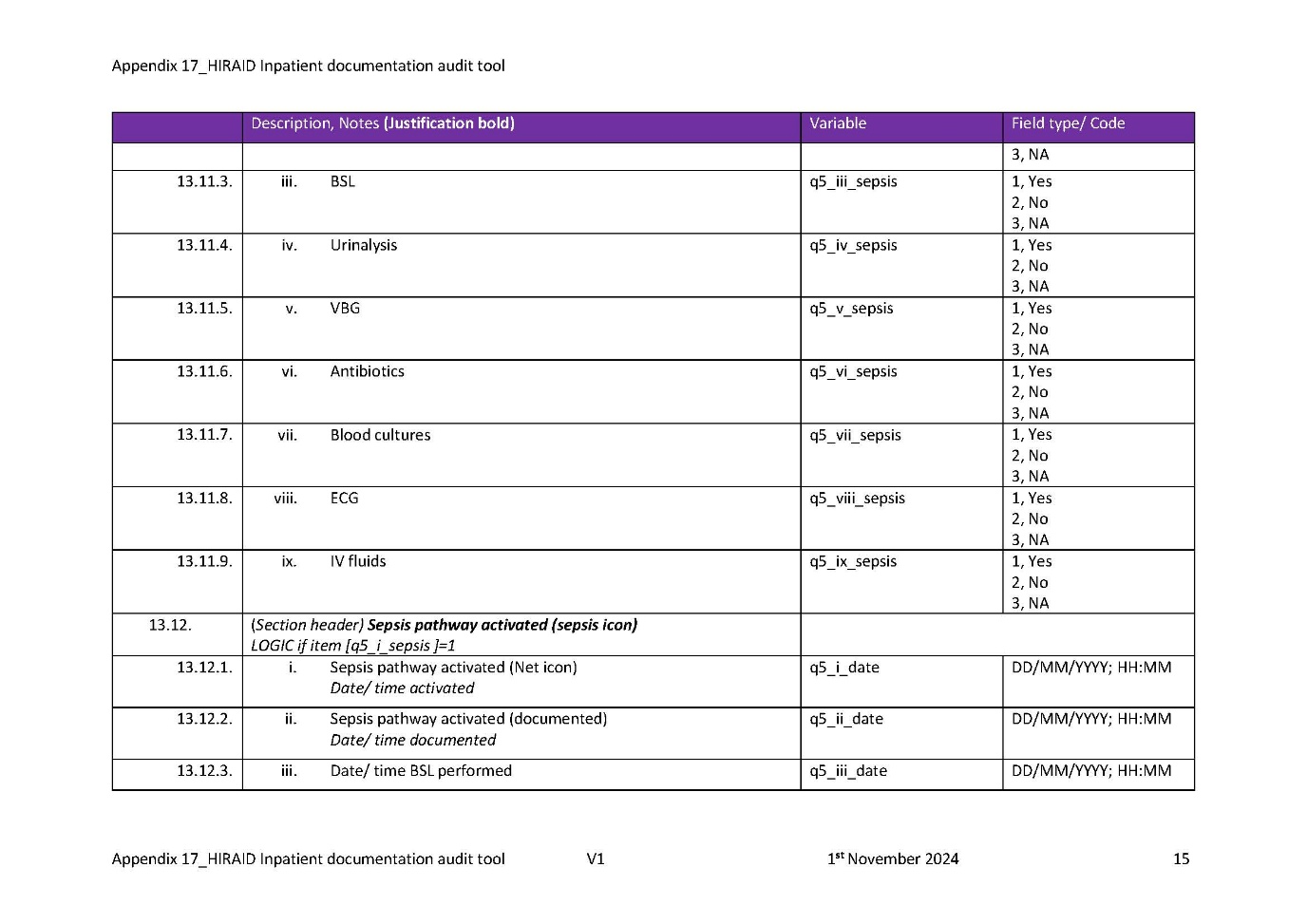


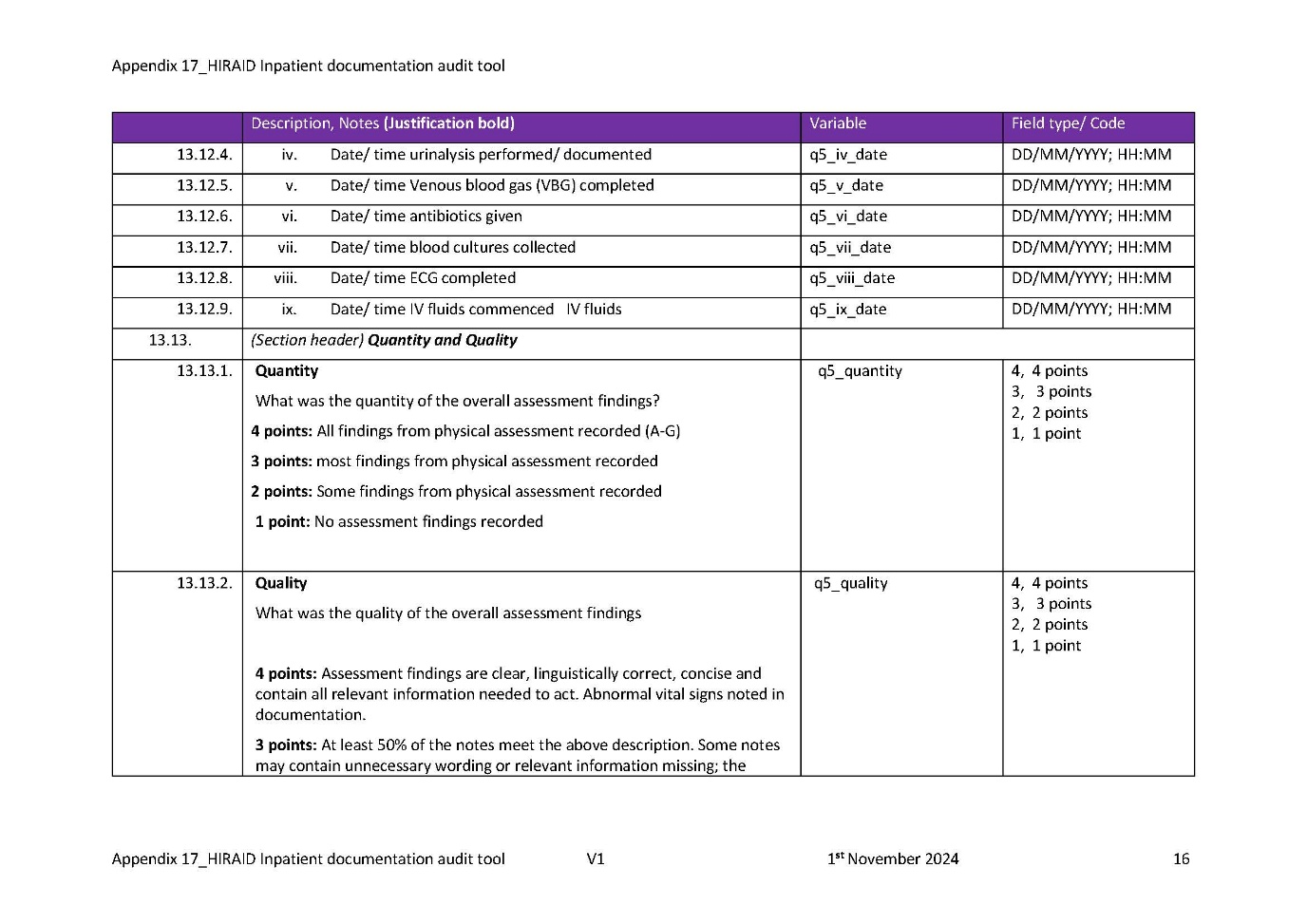


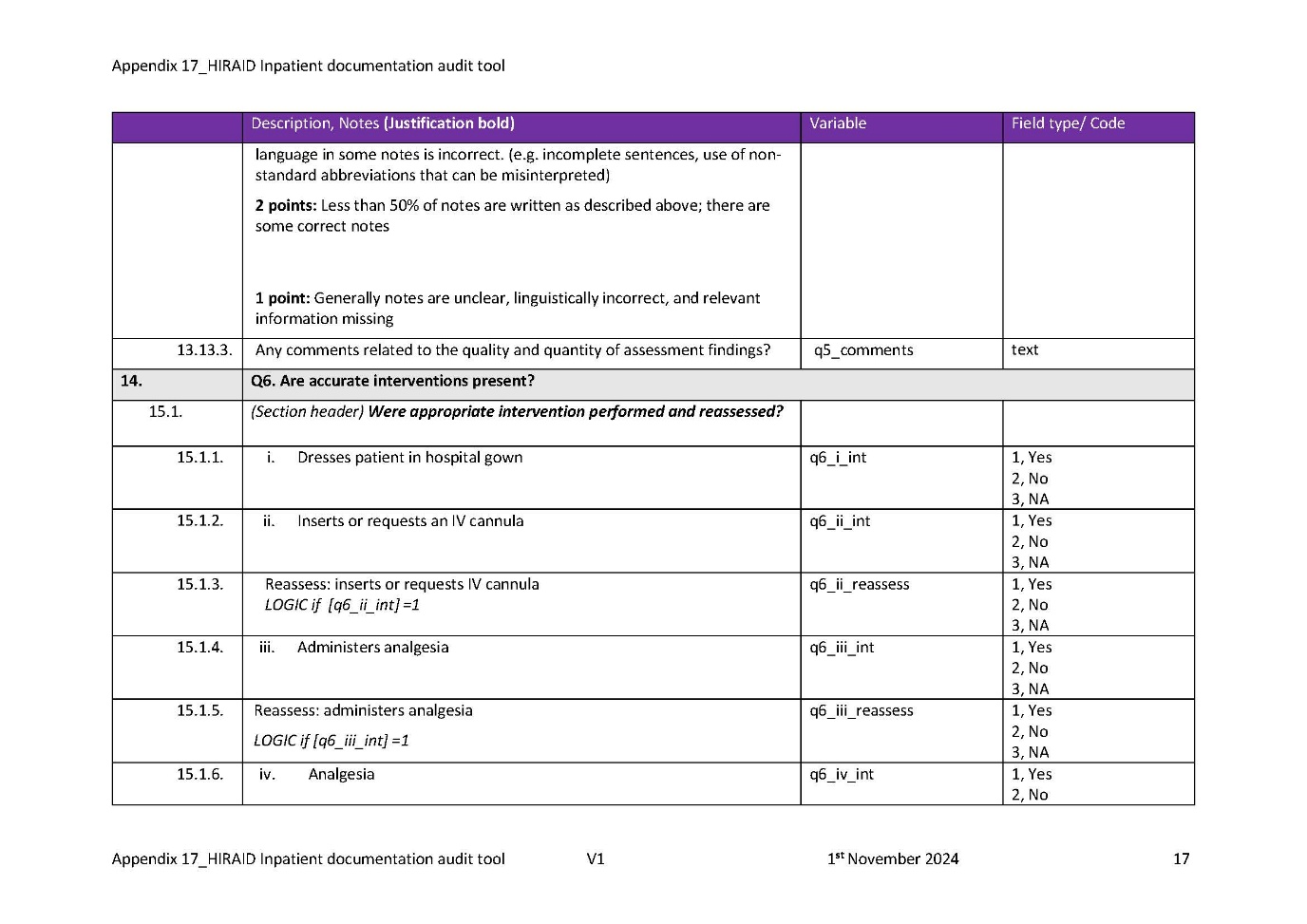


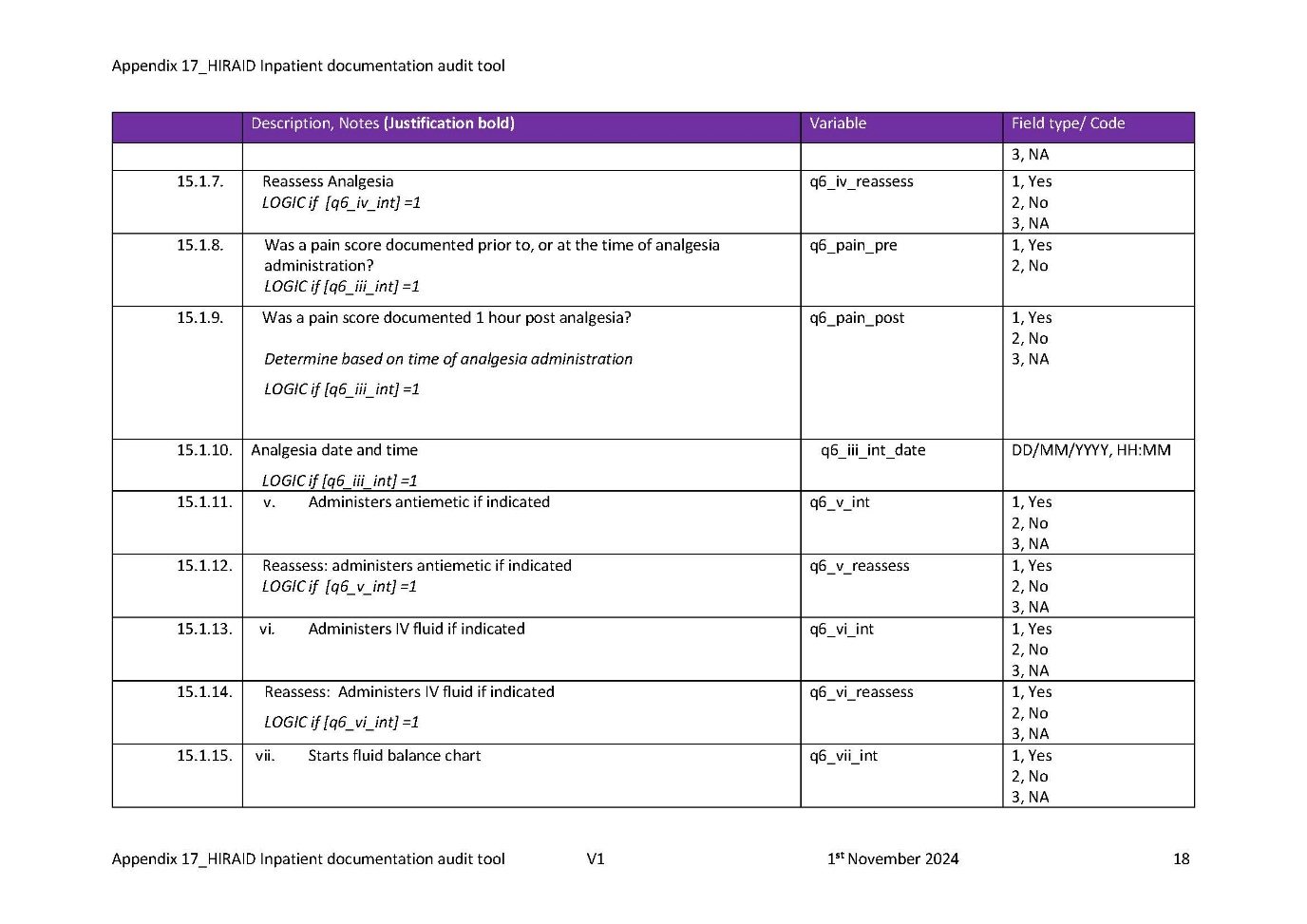


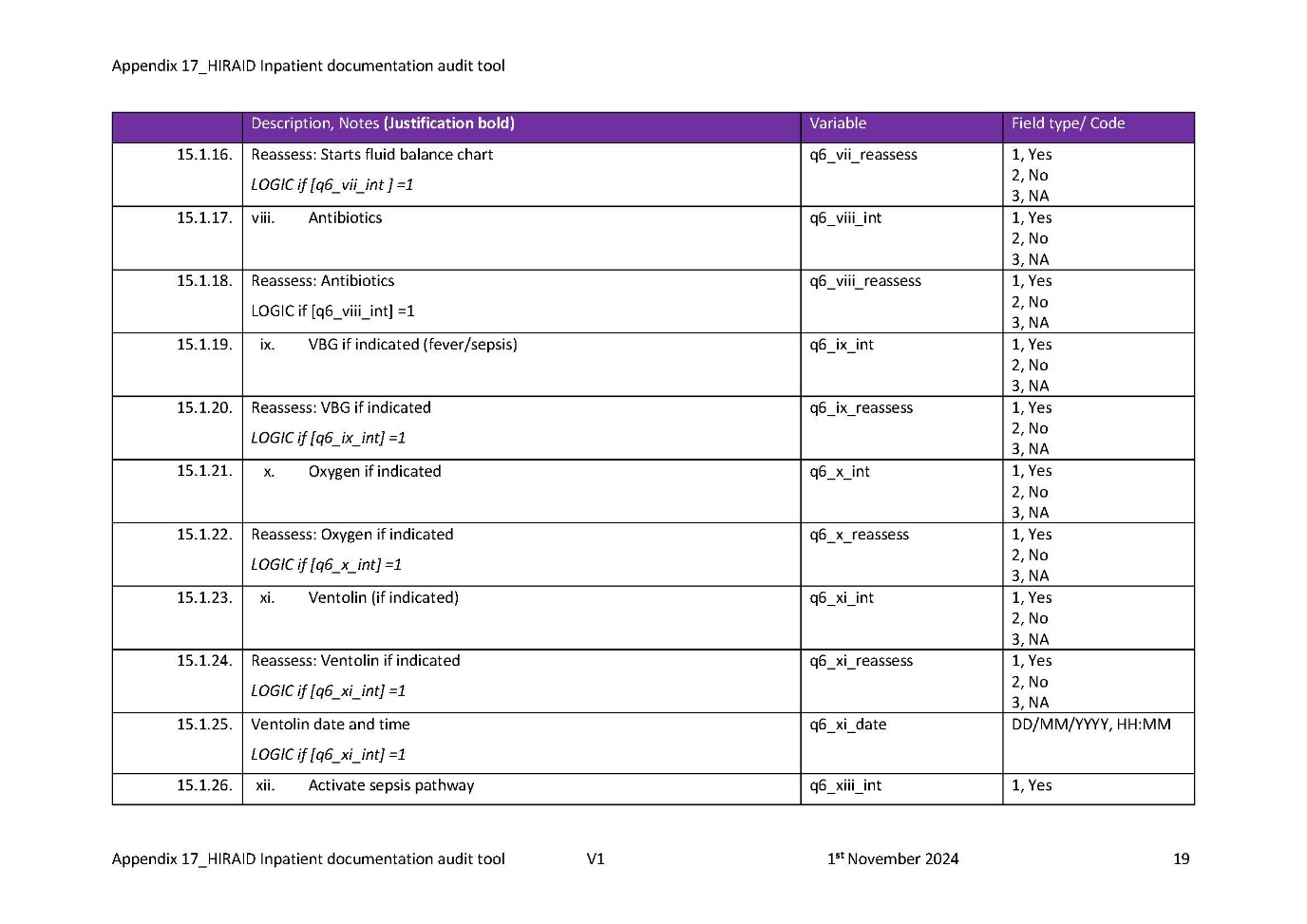


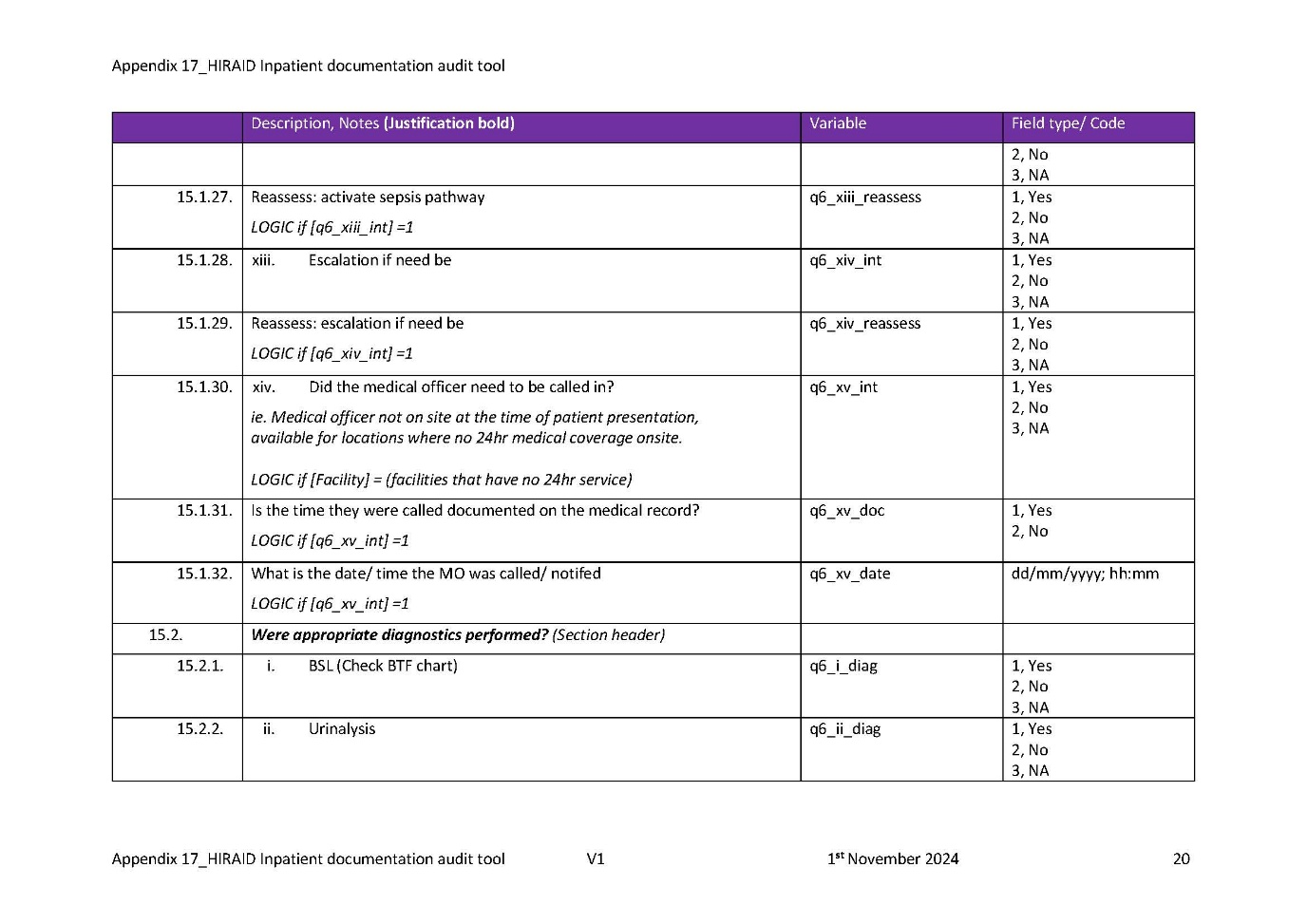


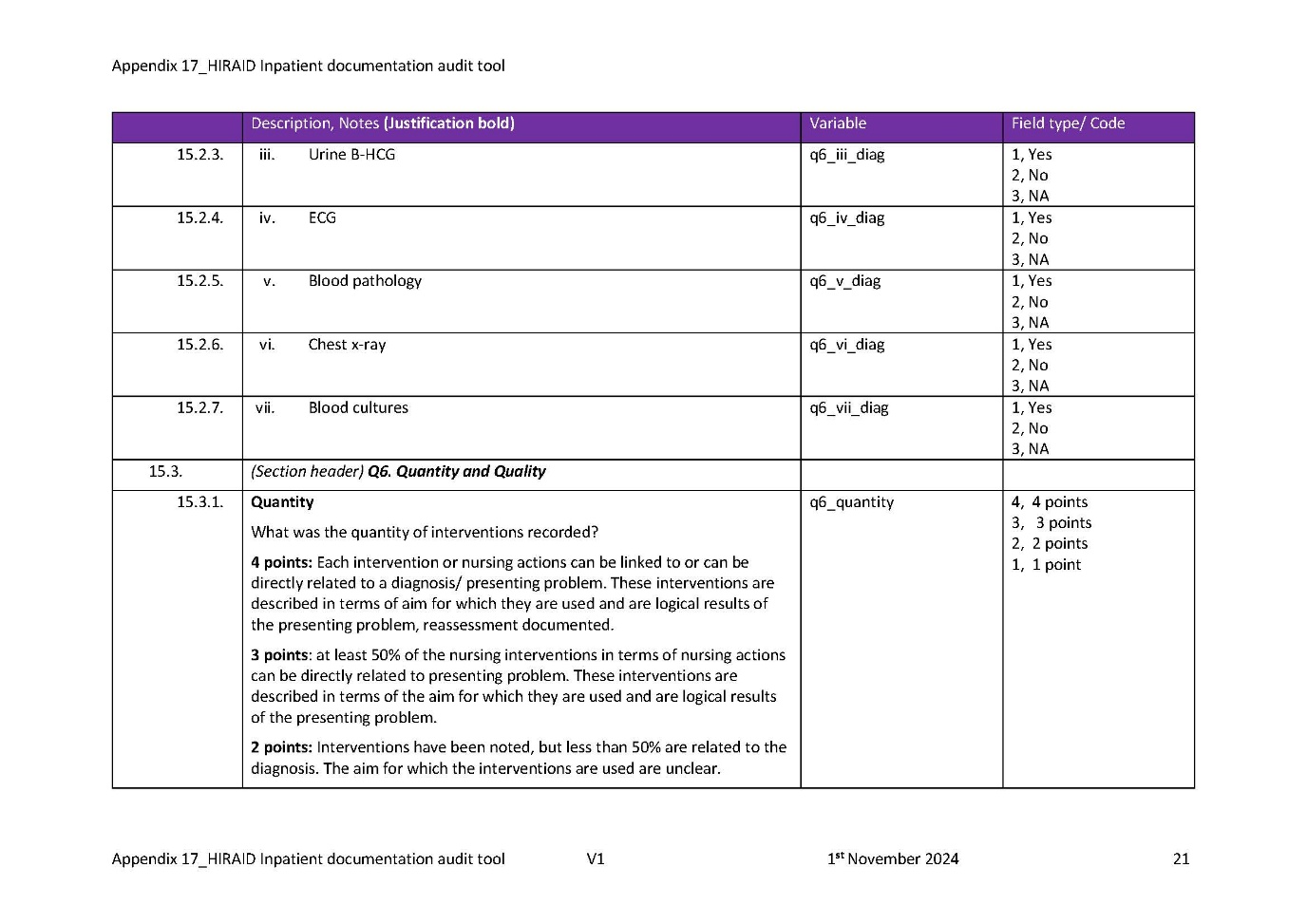


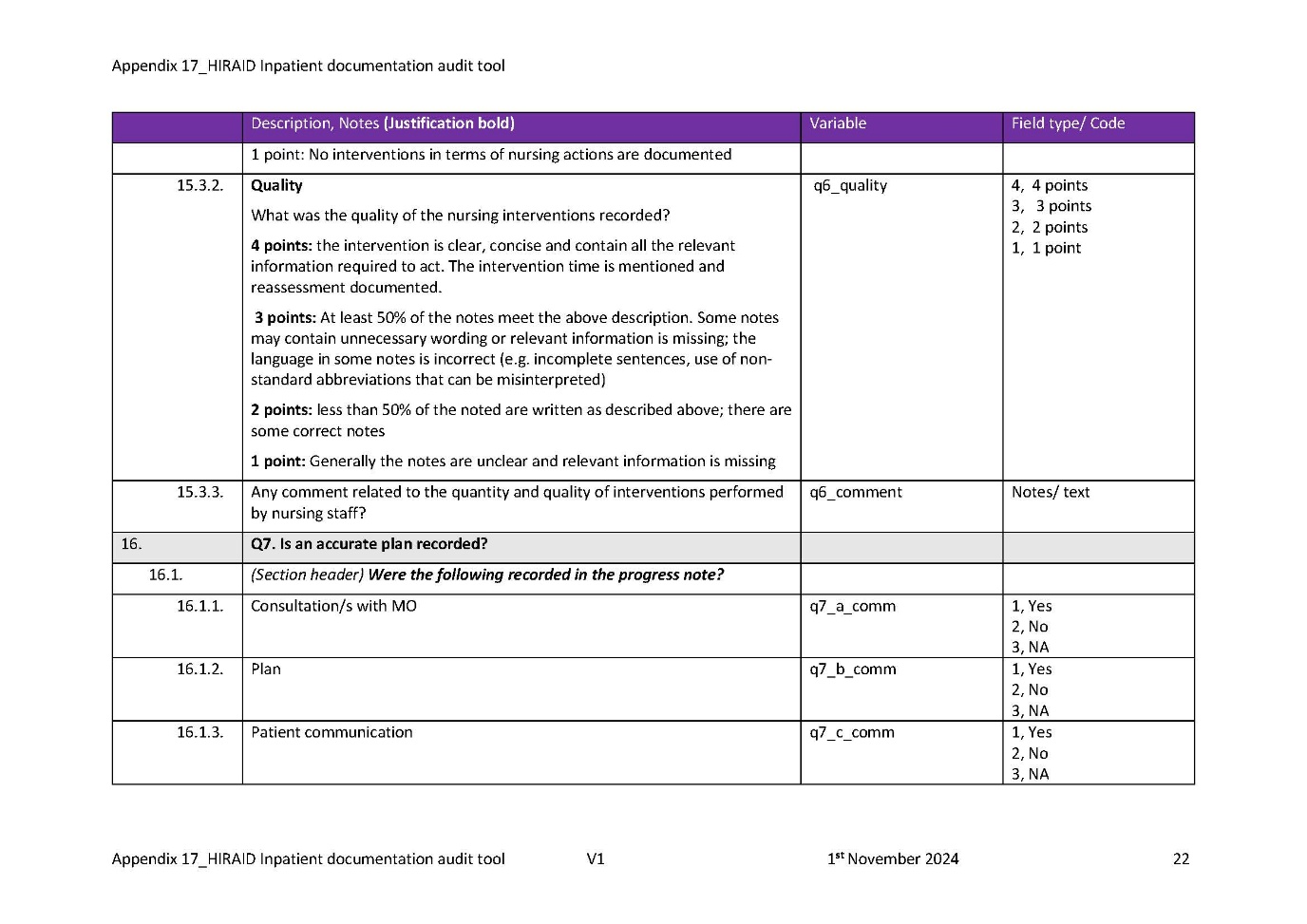


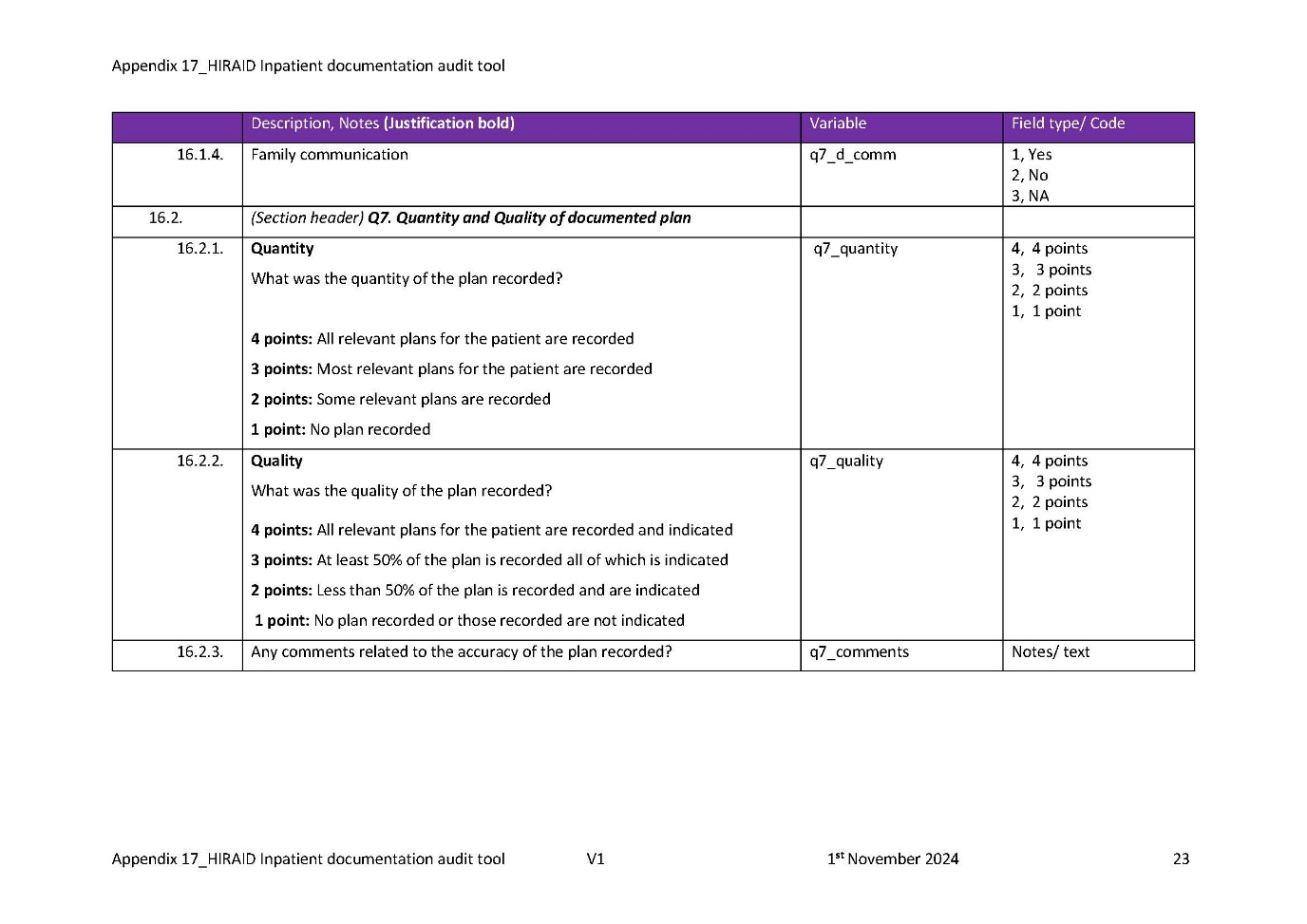


## Supplement 2


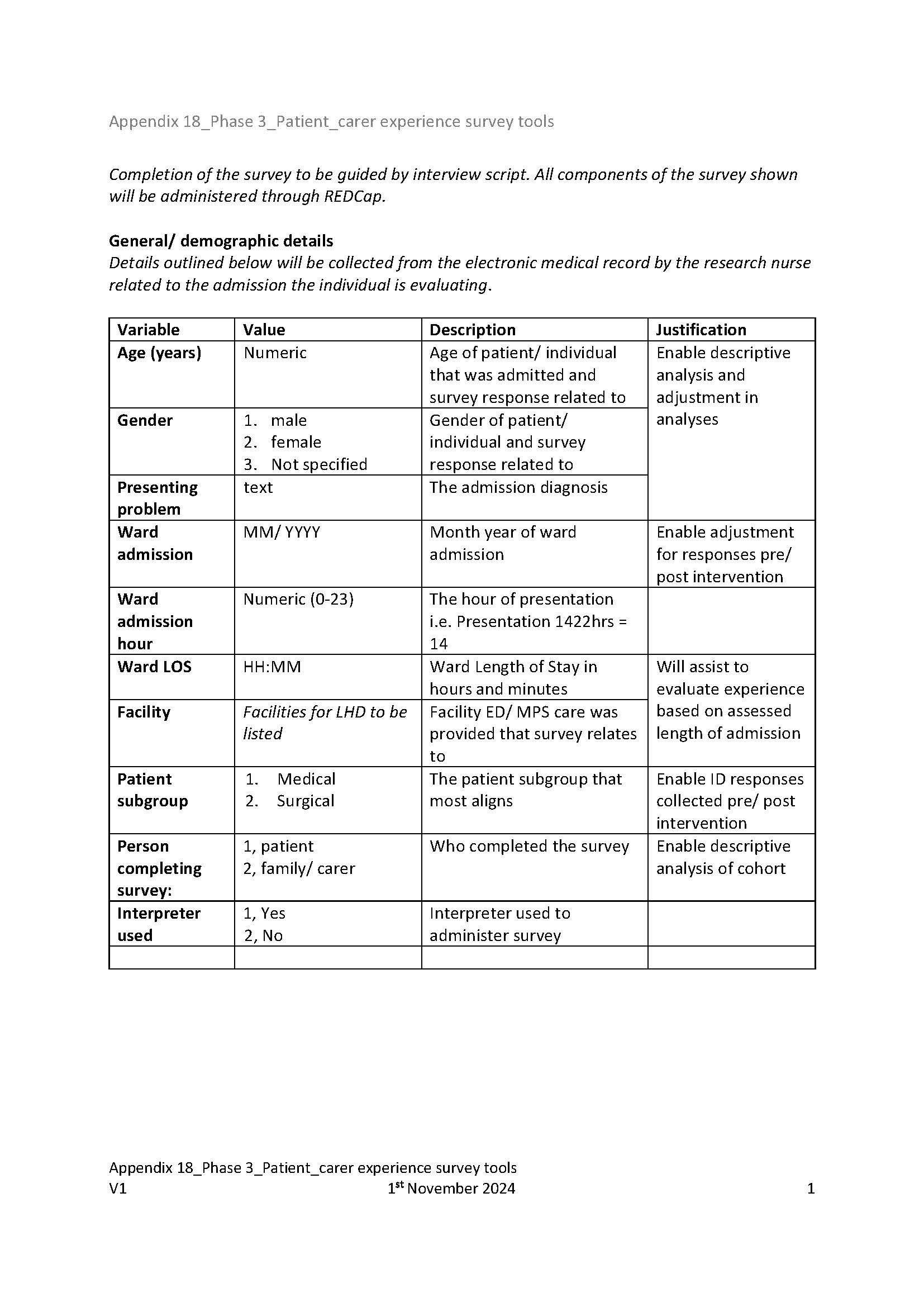


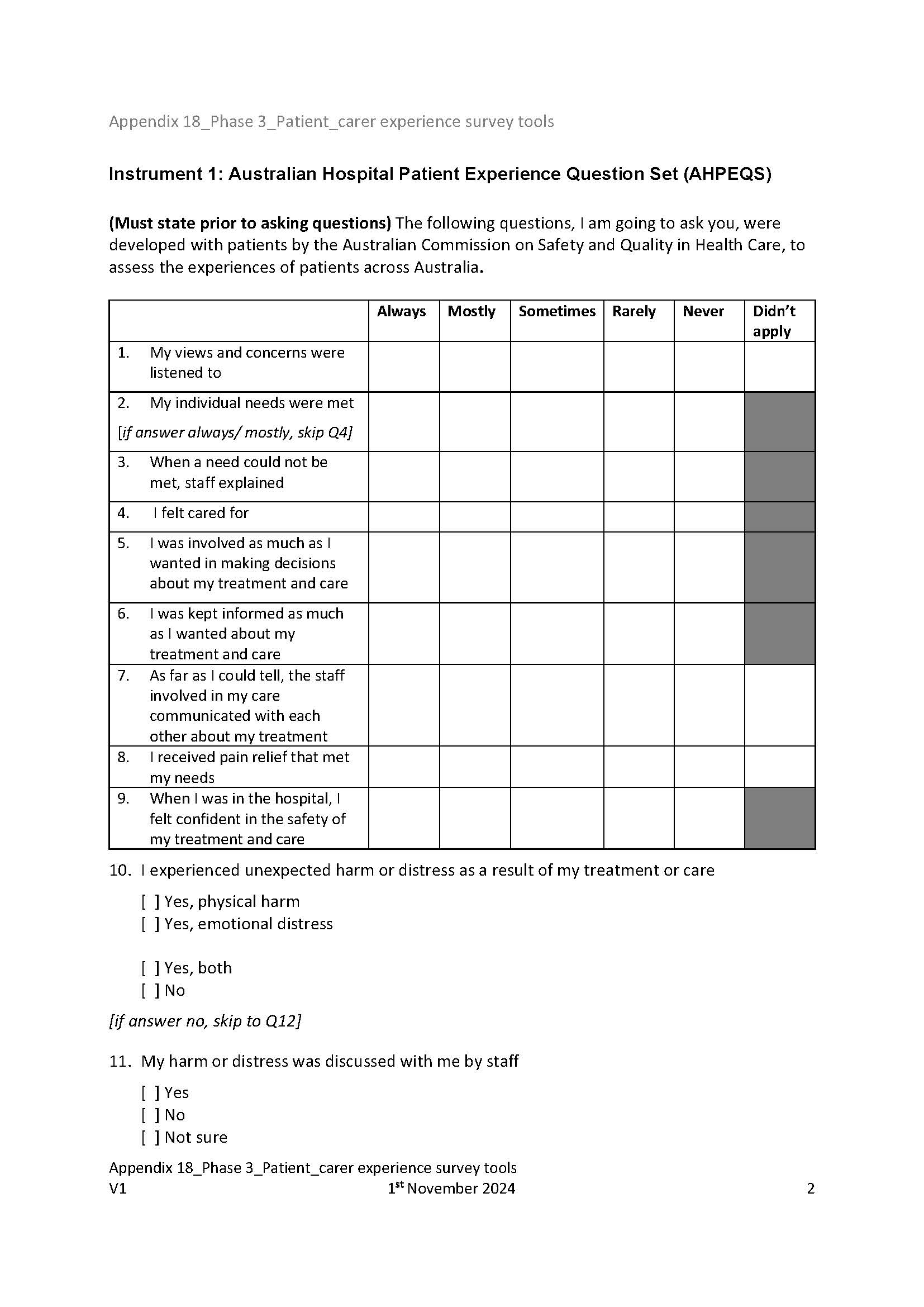


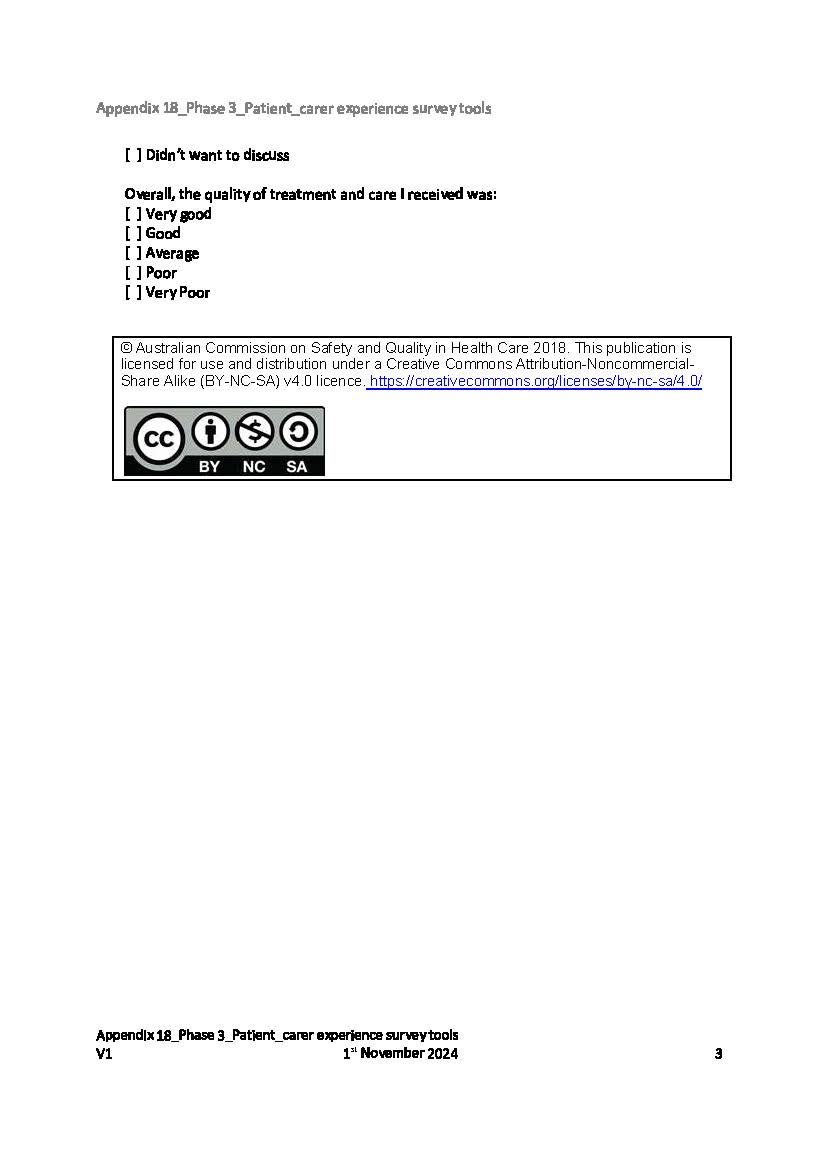


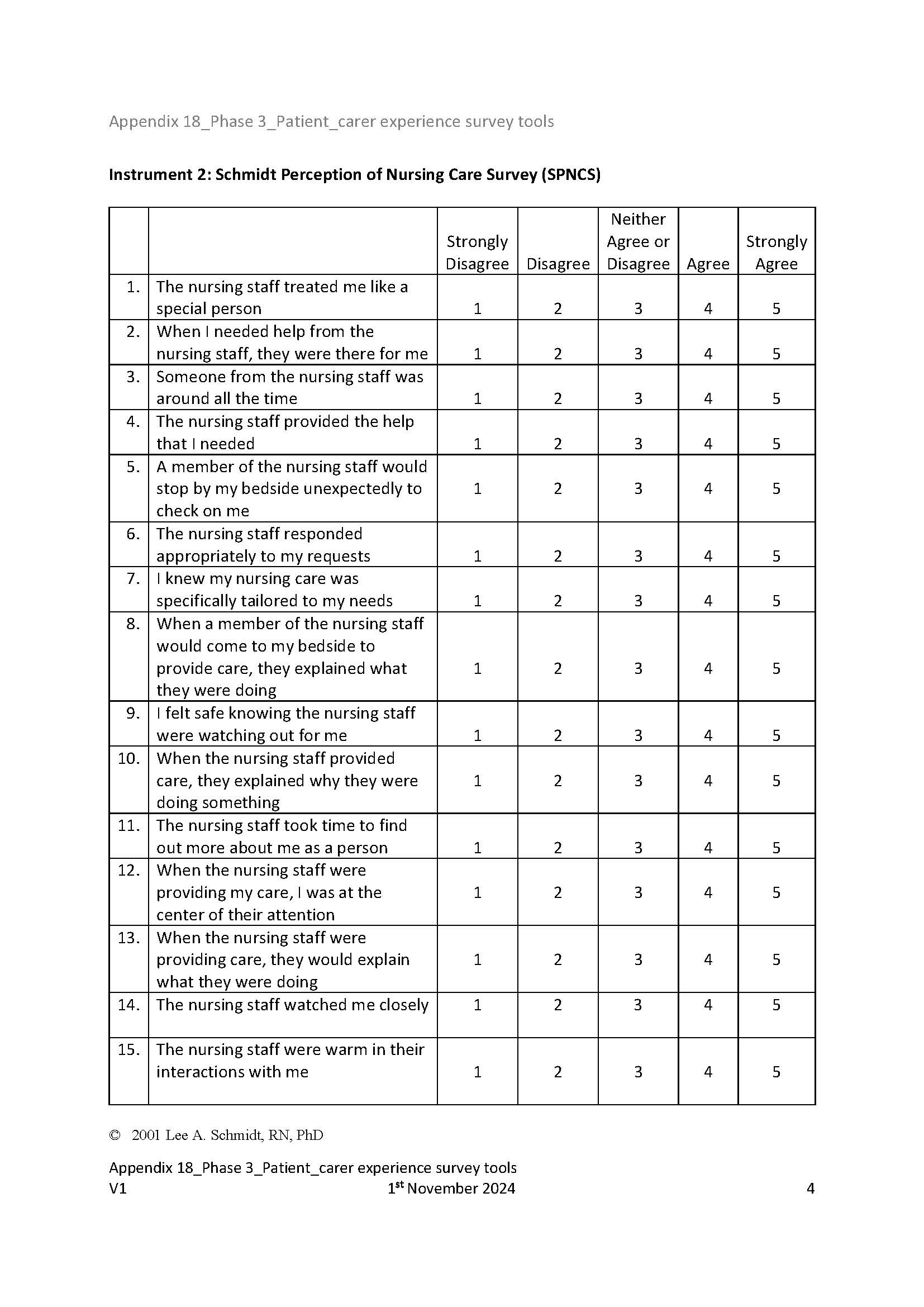


## Supplement 3


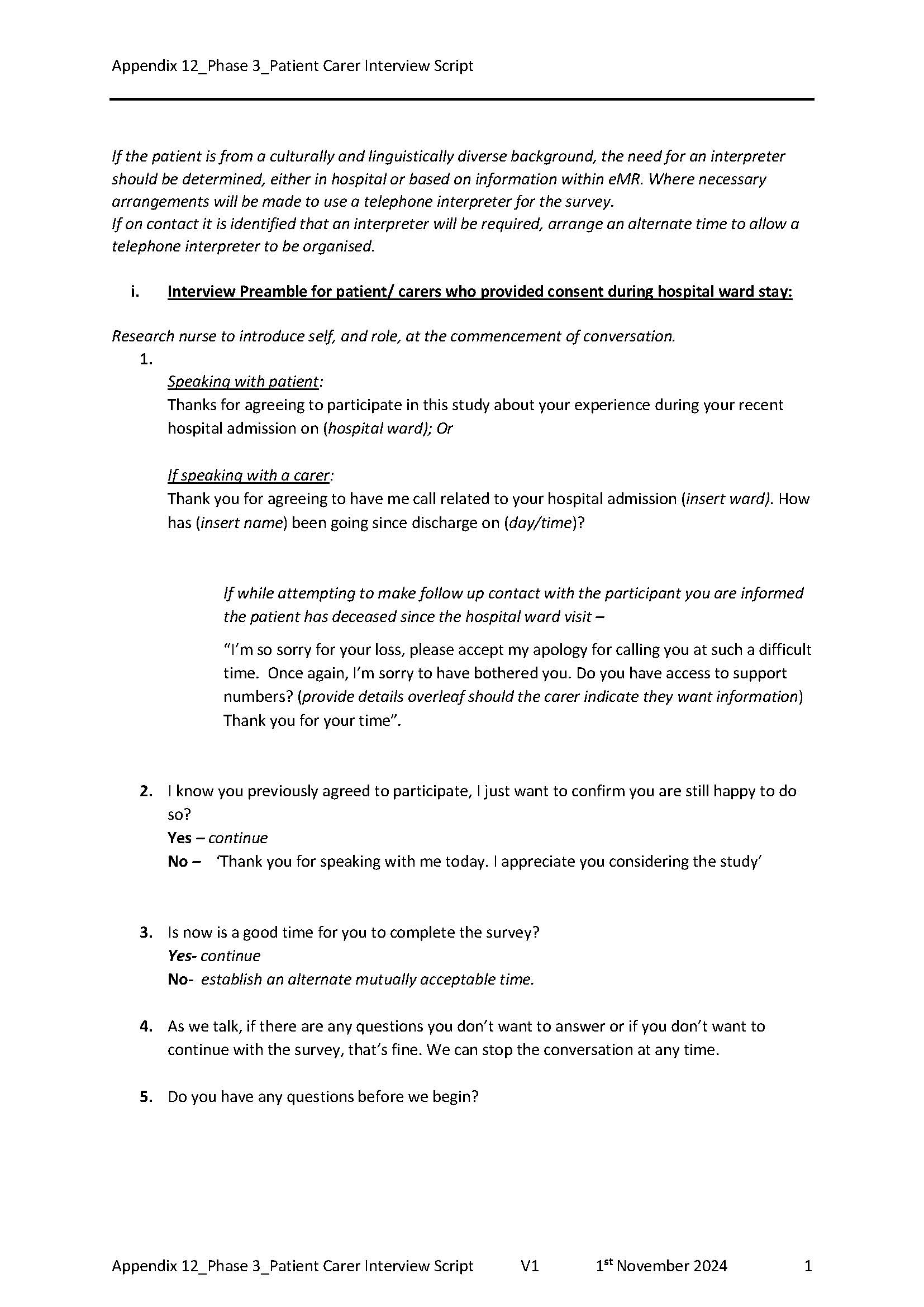


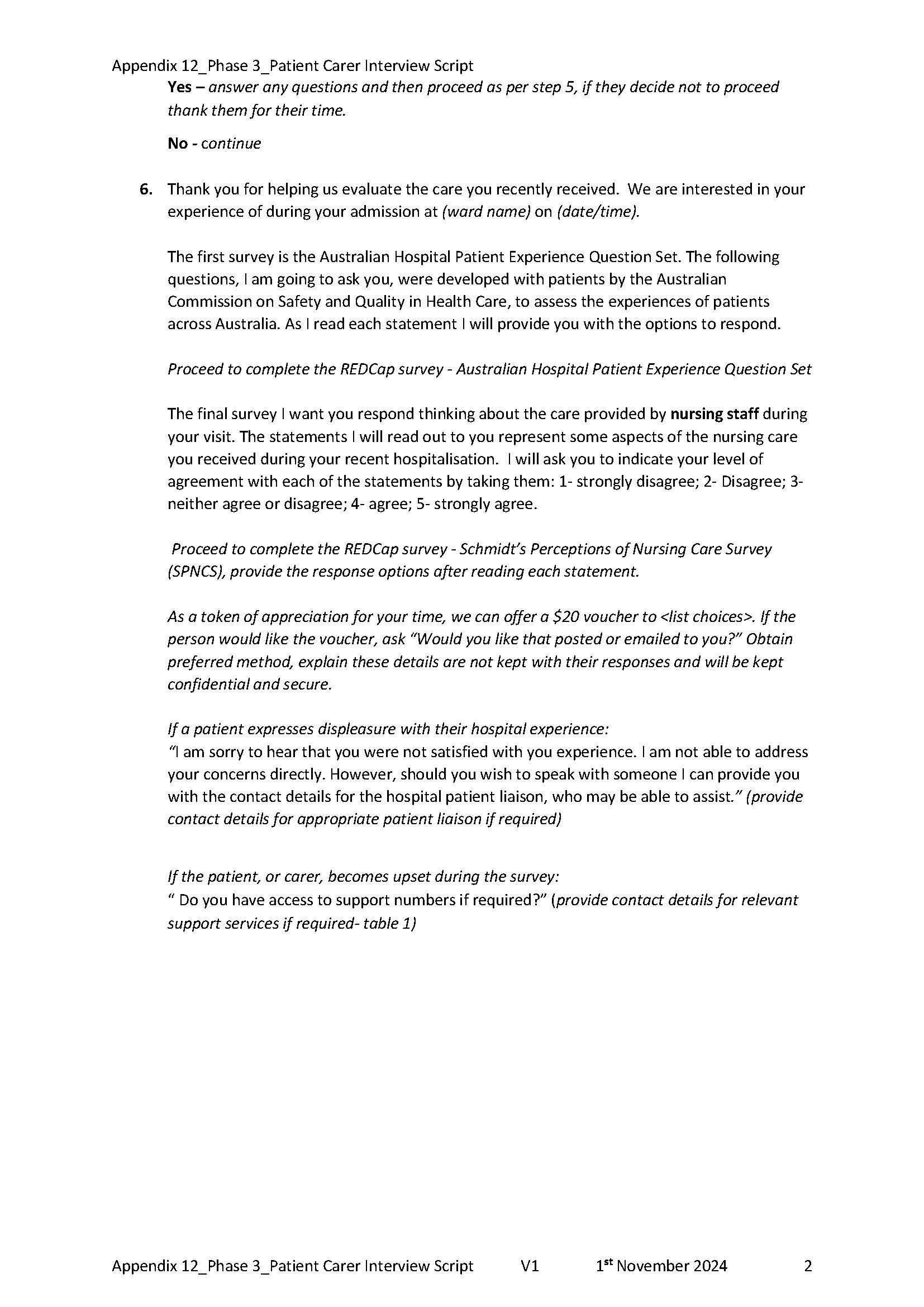

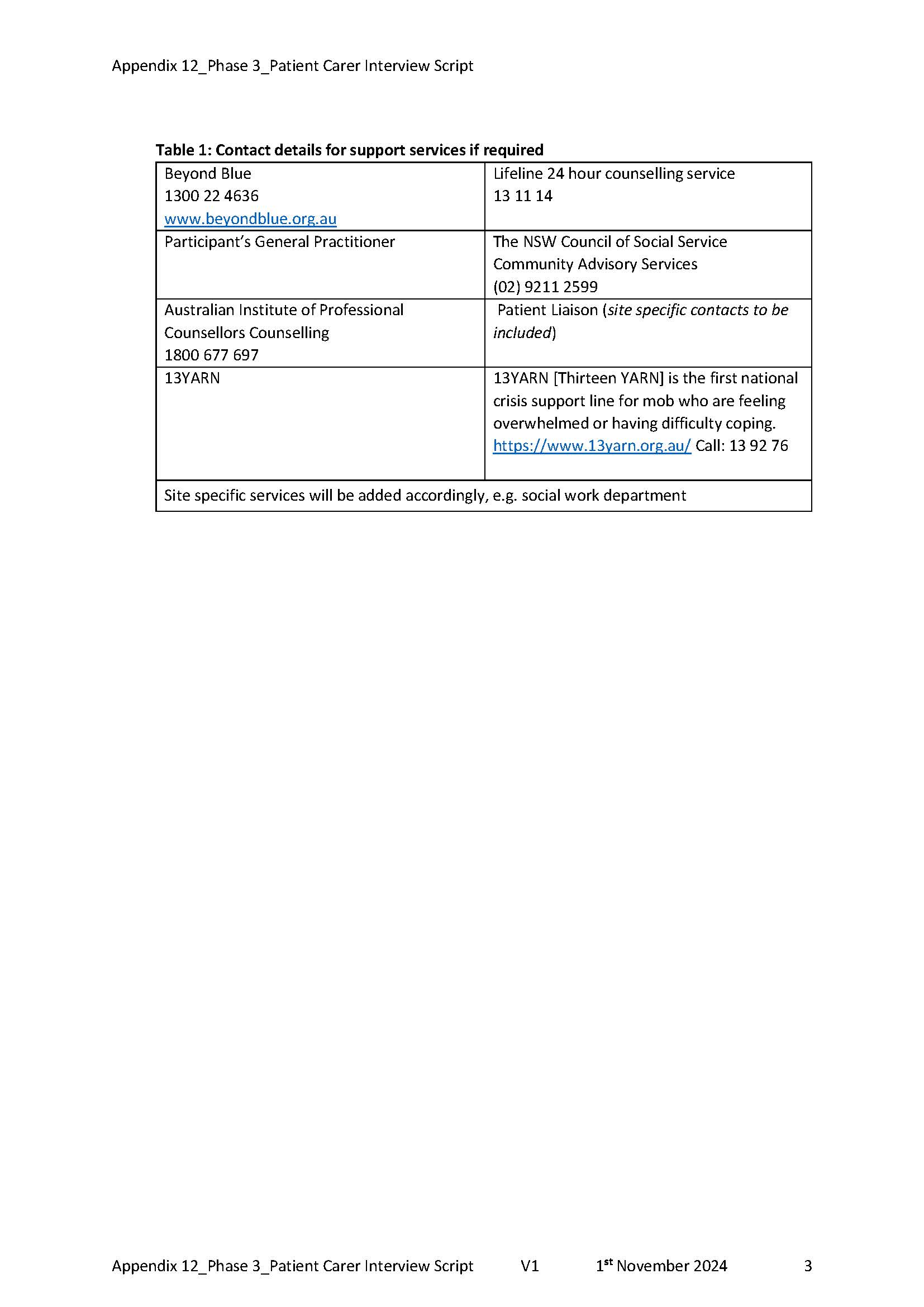


## Supplement 4


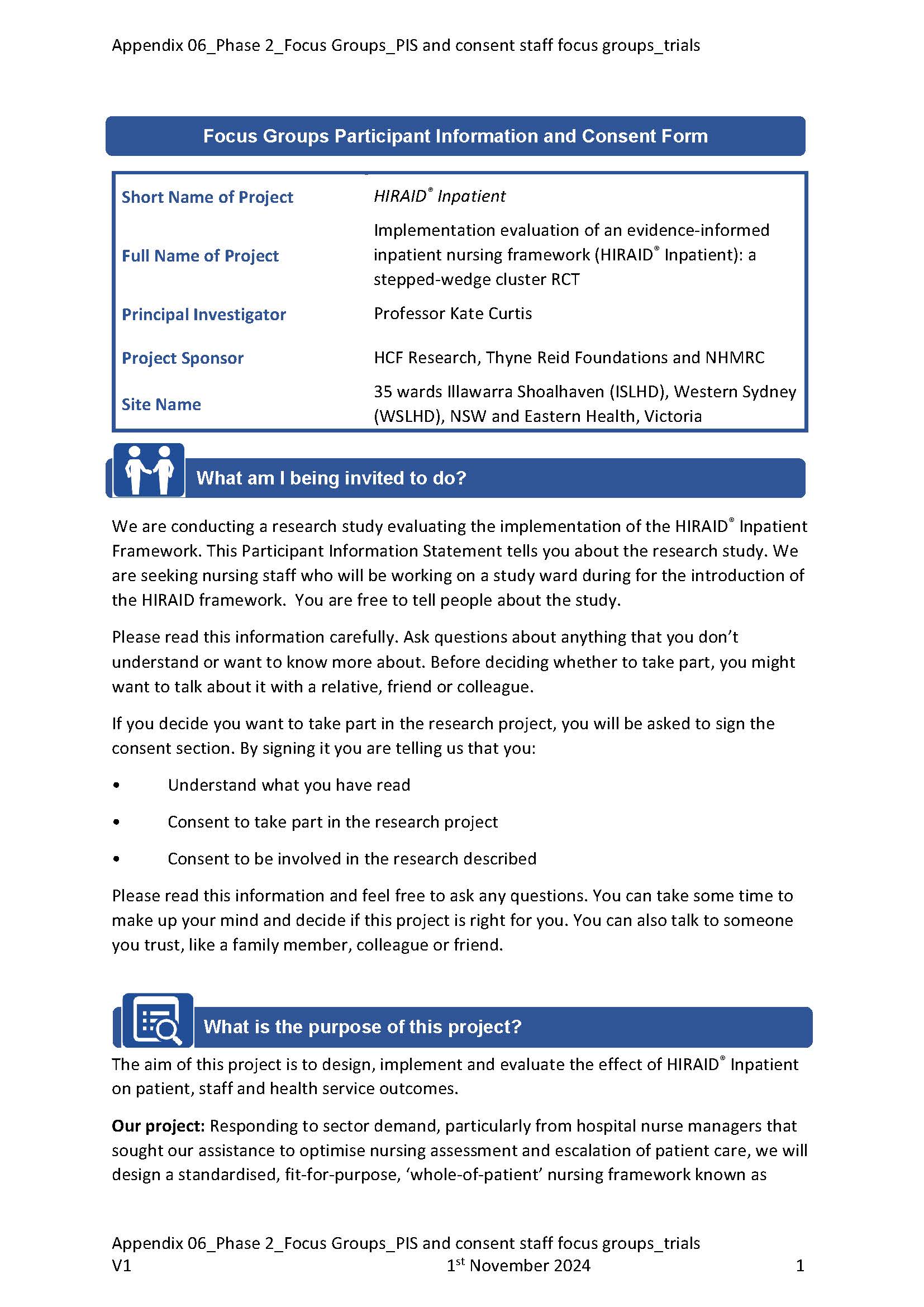


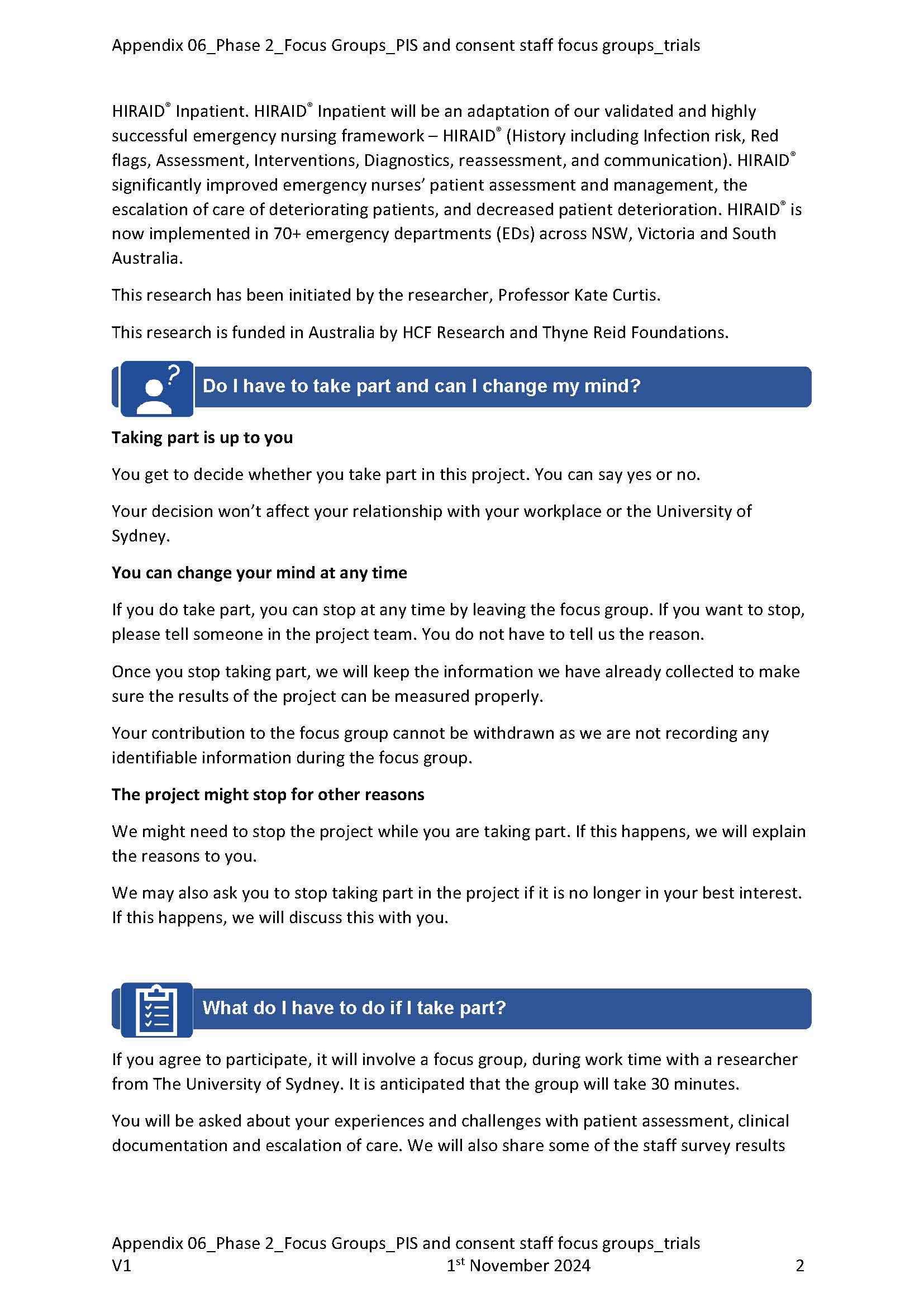


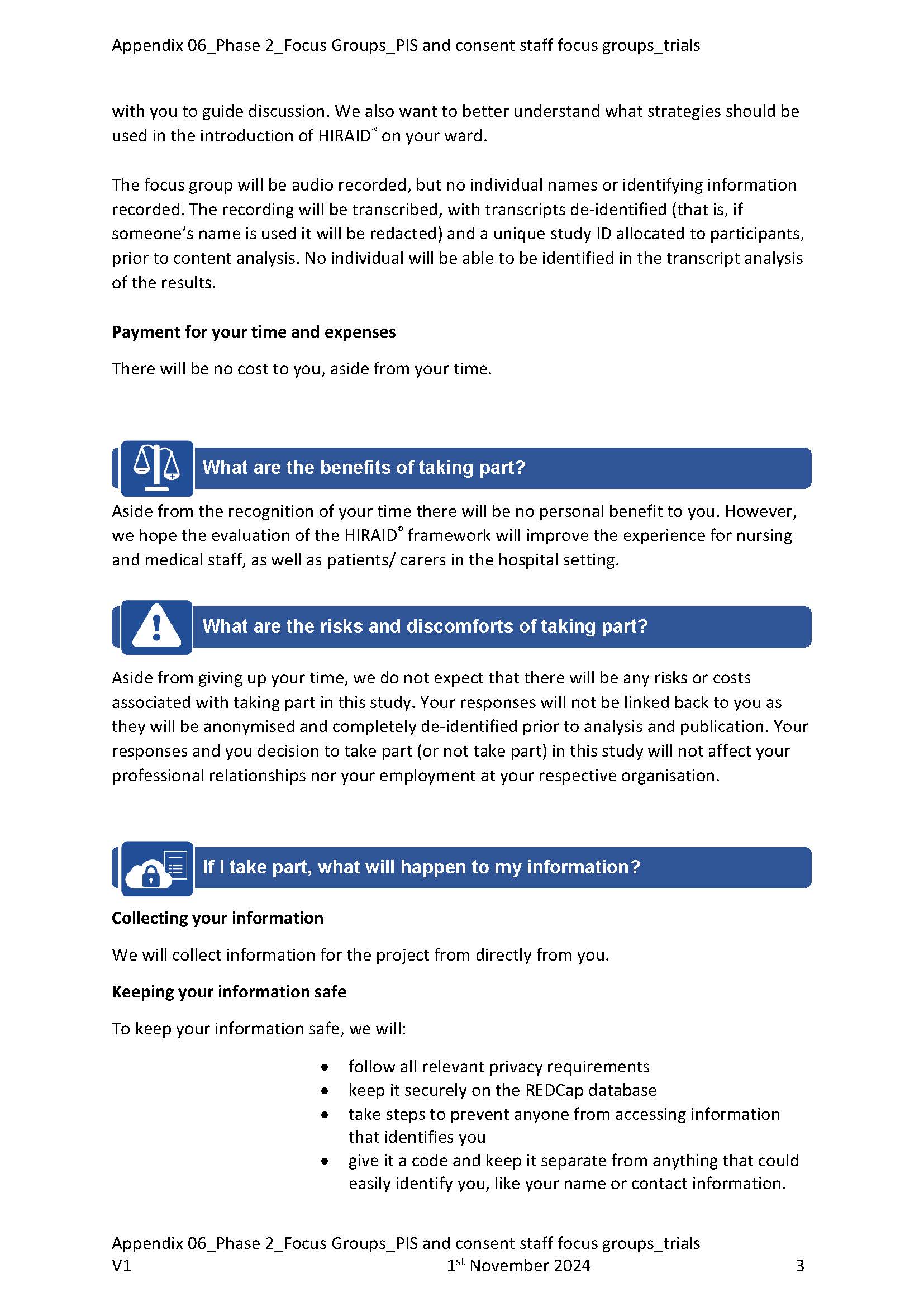

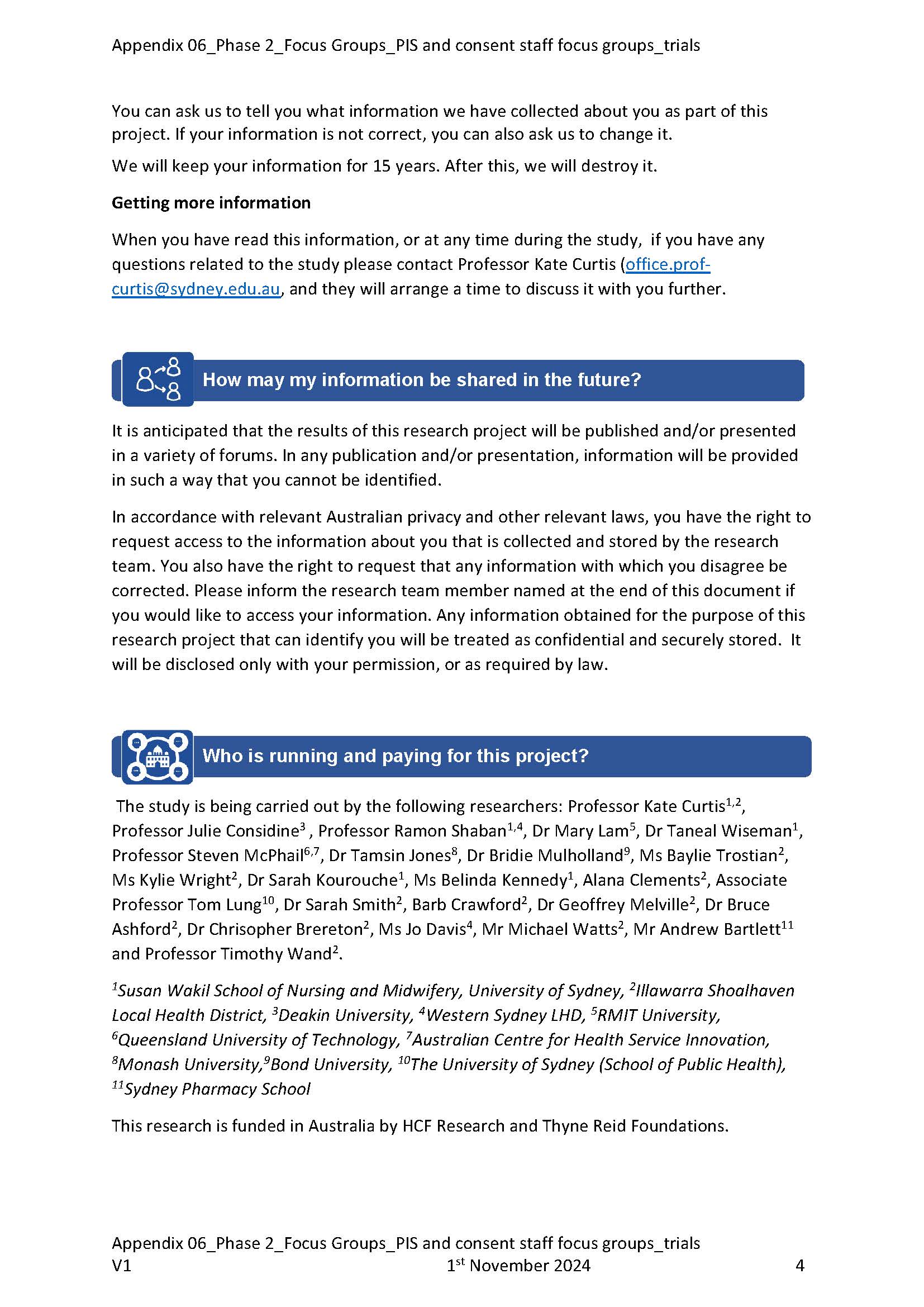

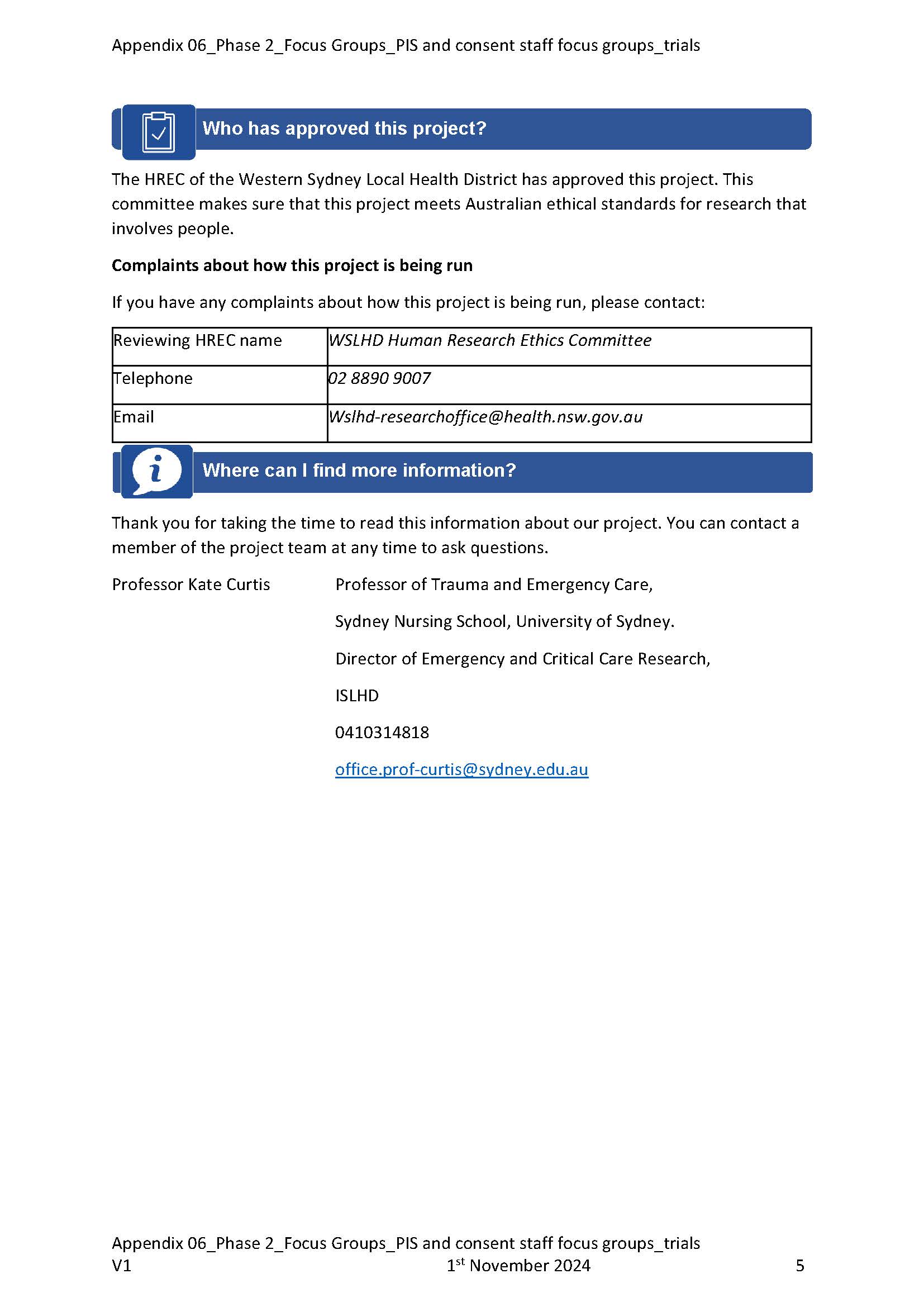

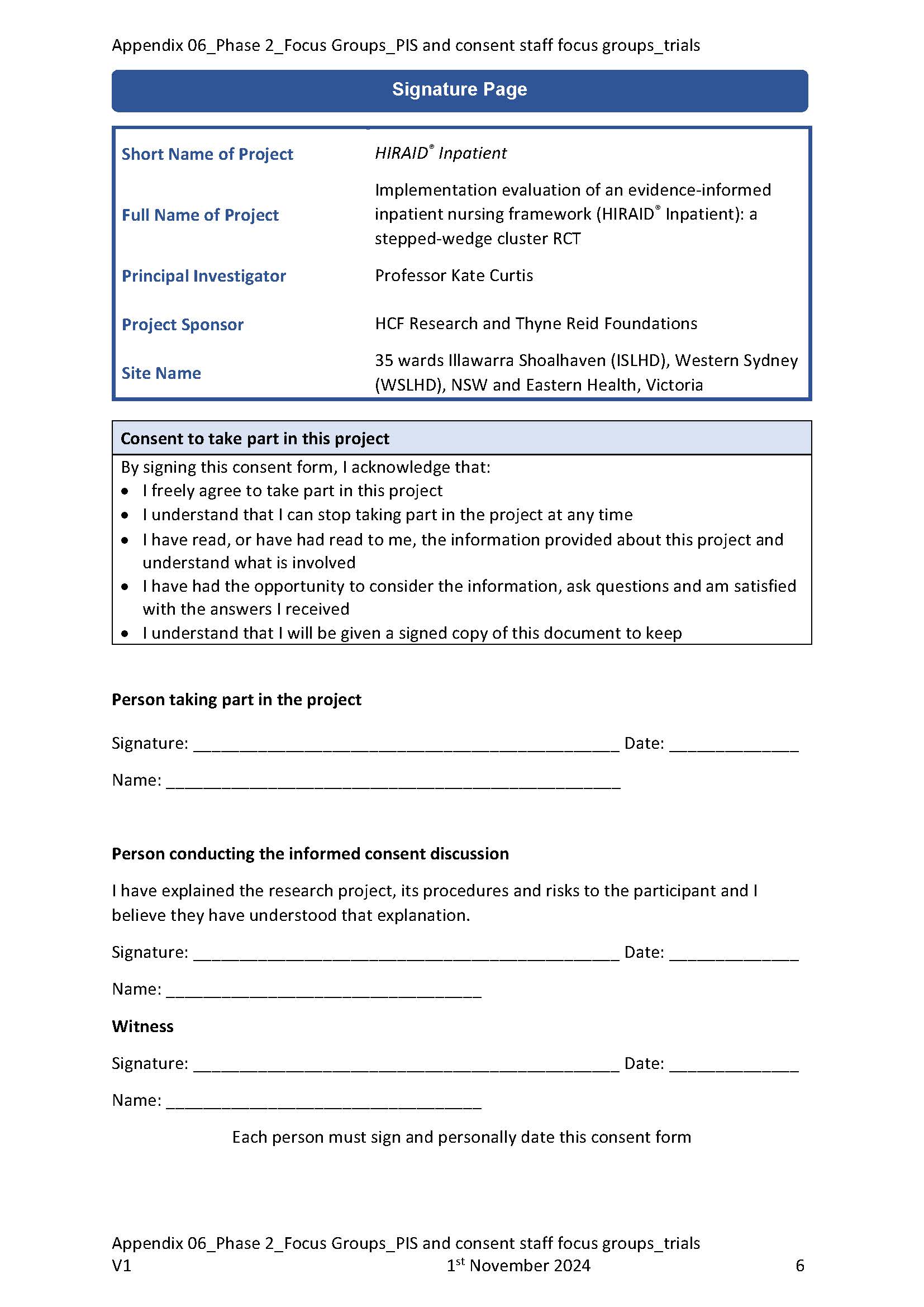


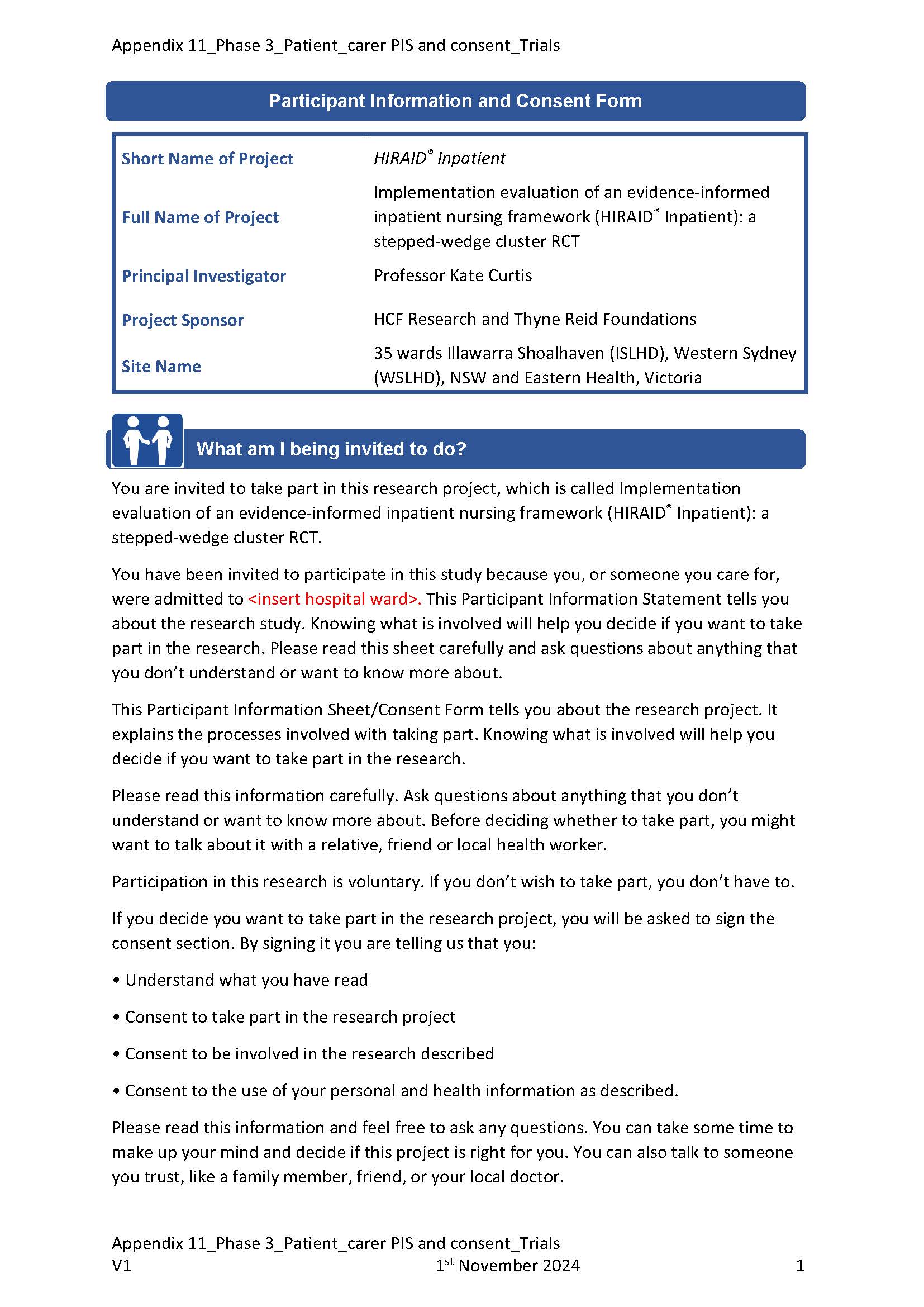


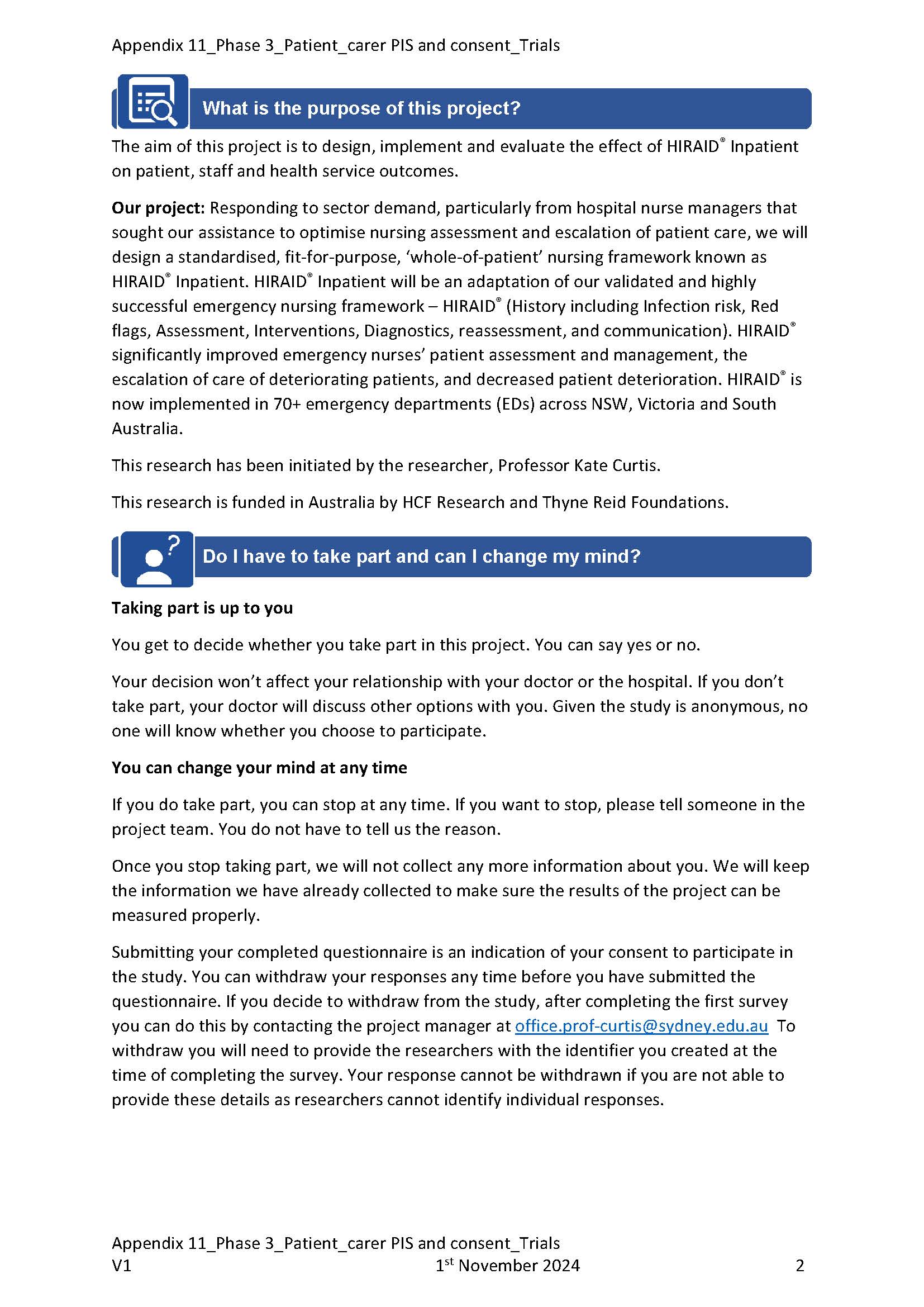

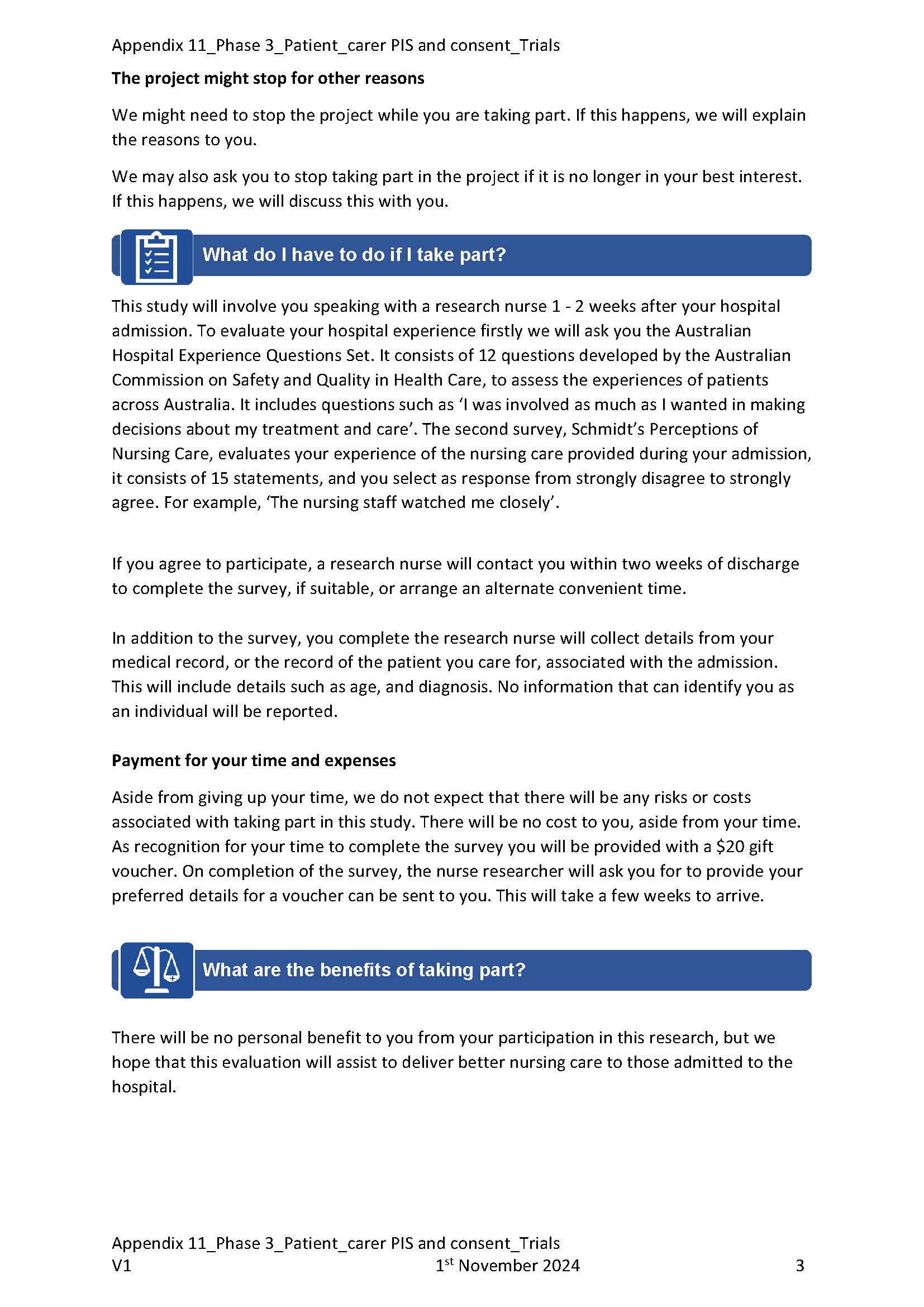

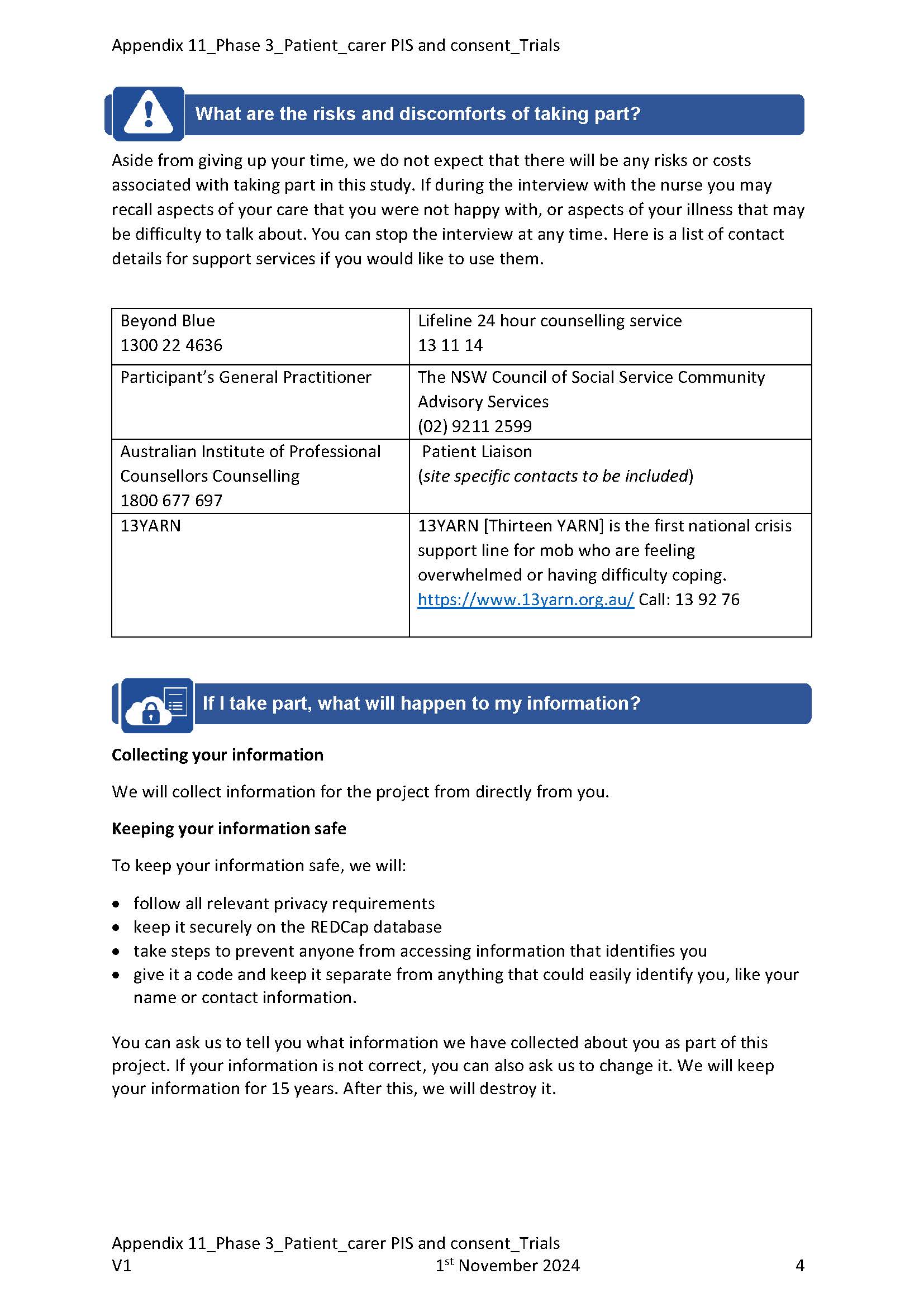

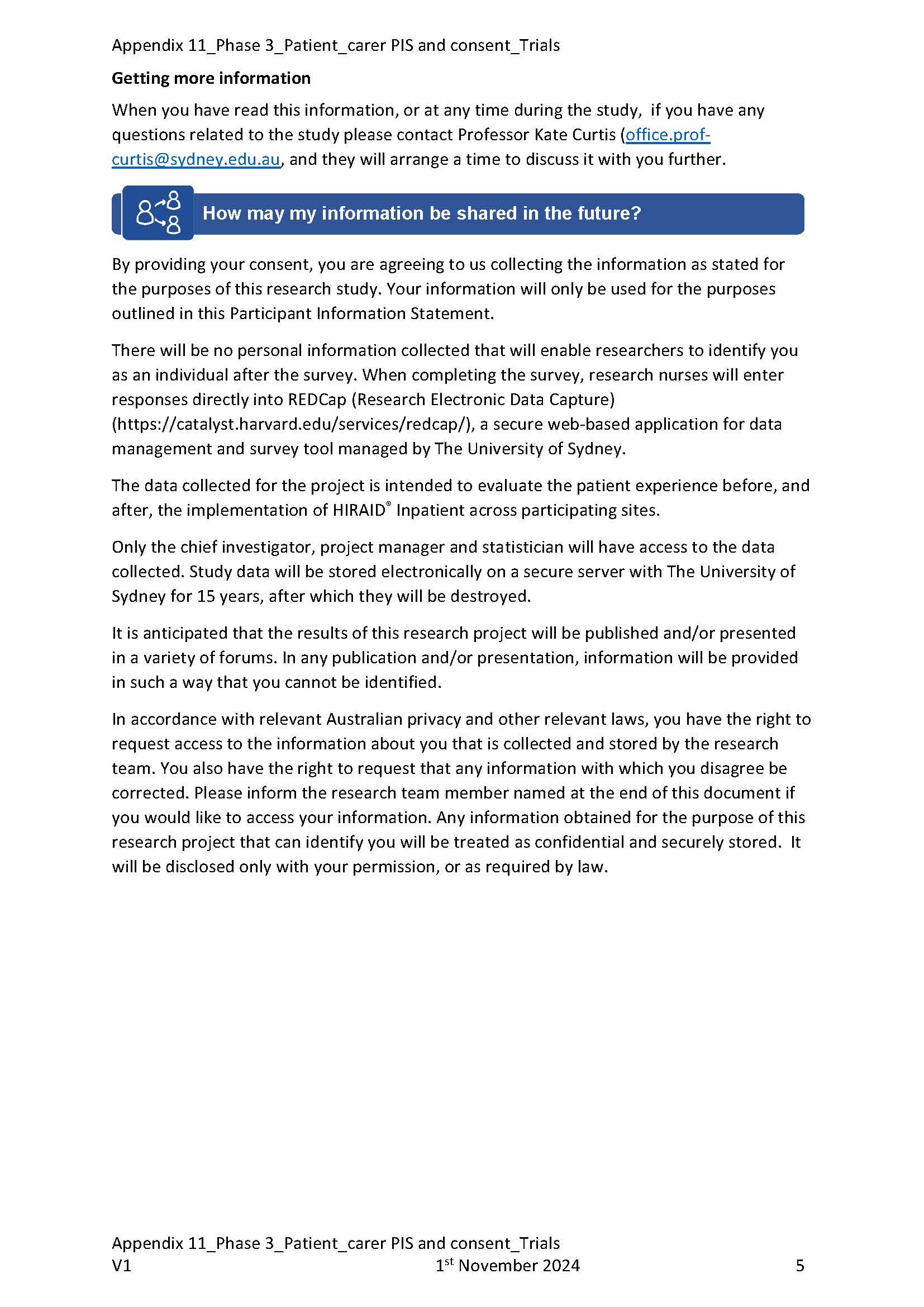

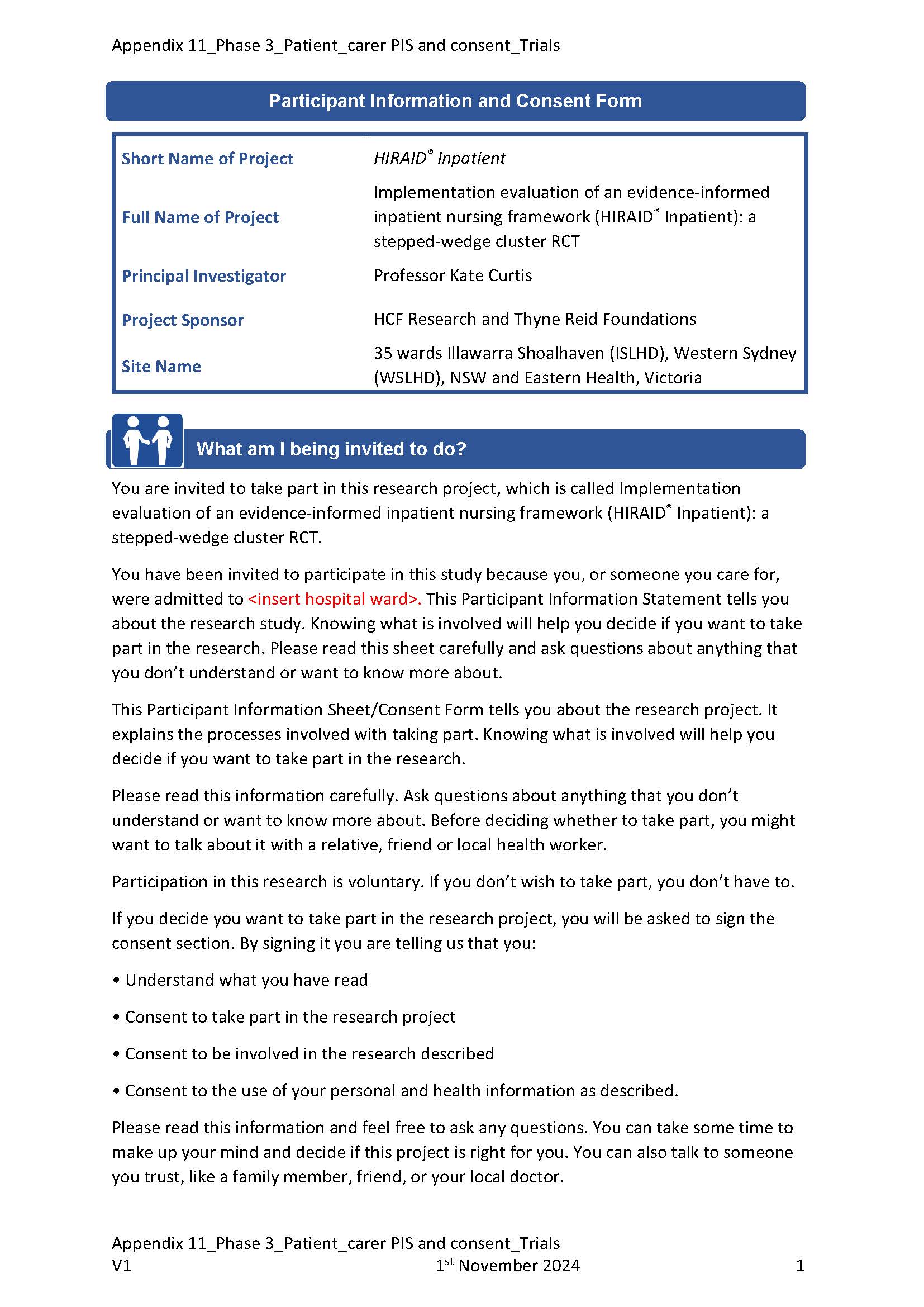

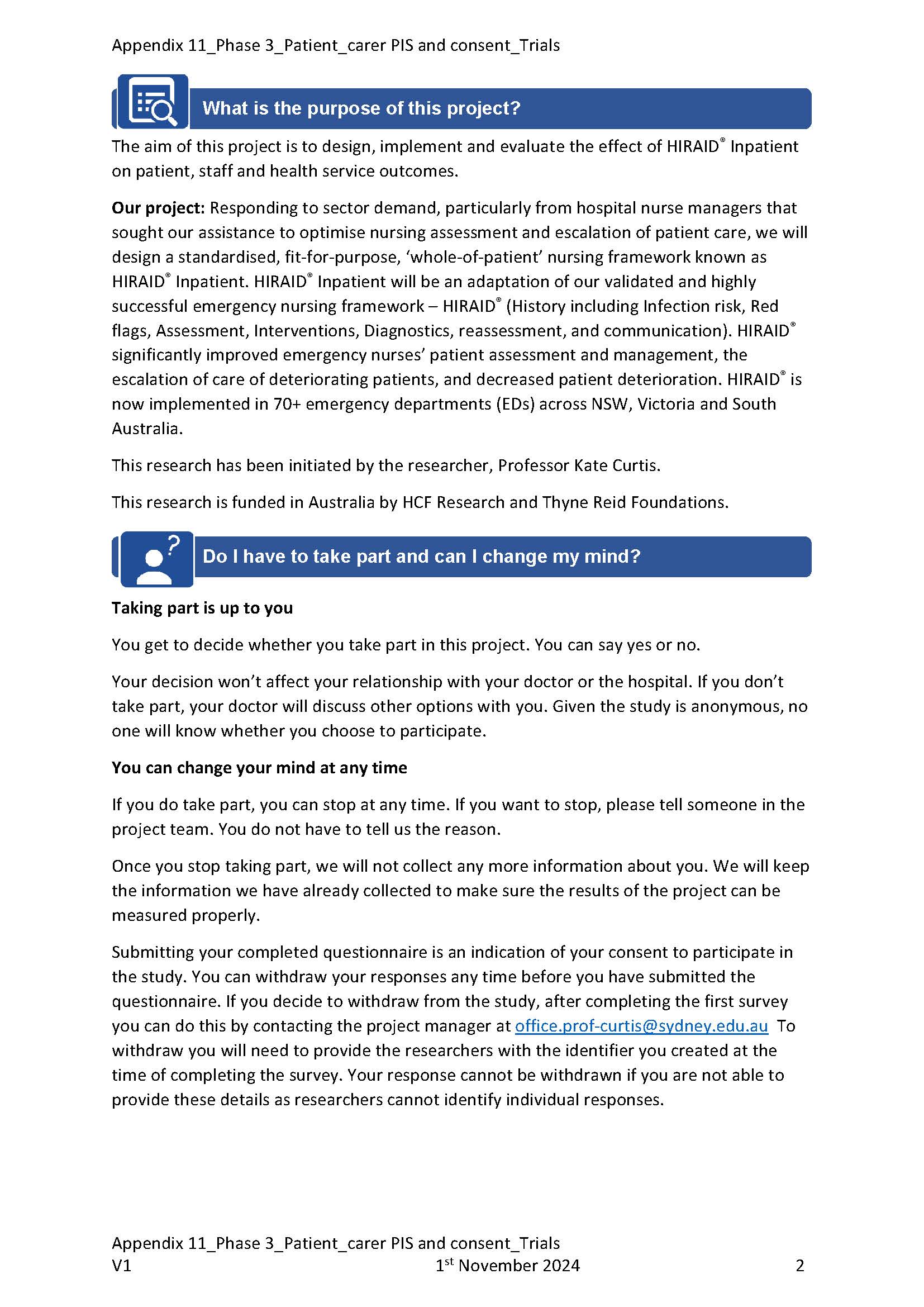


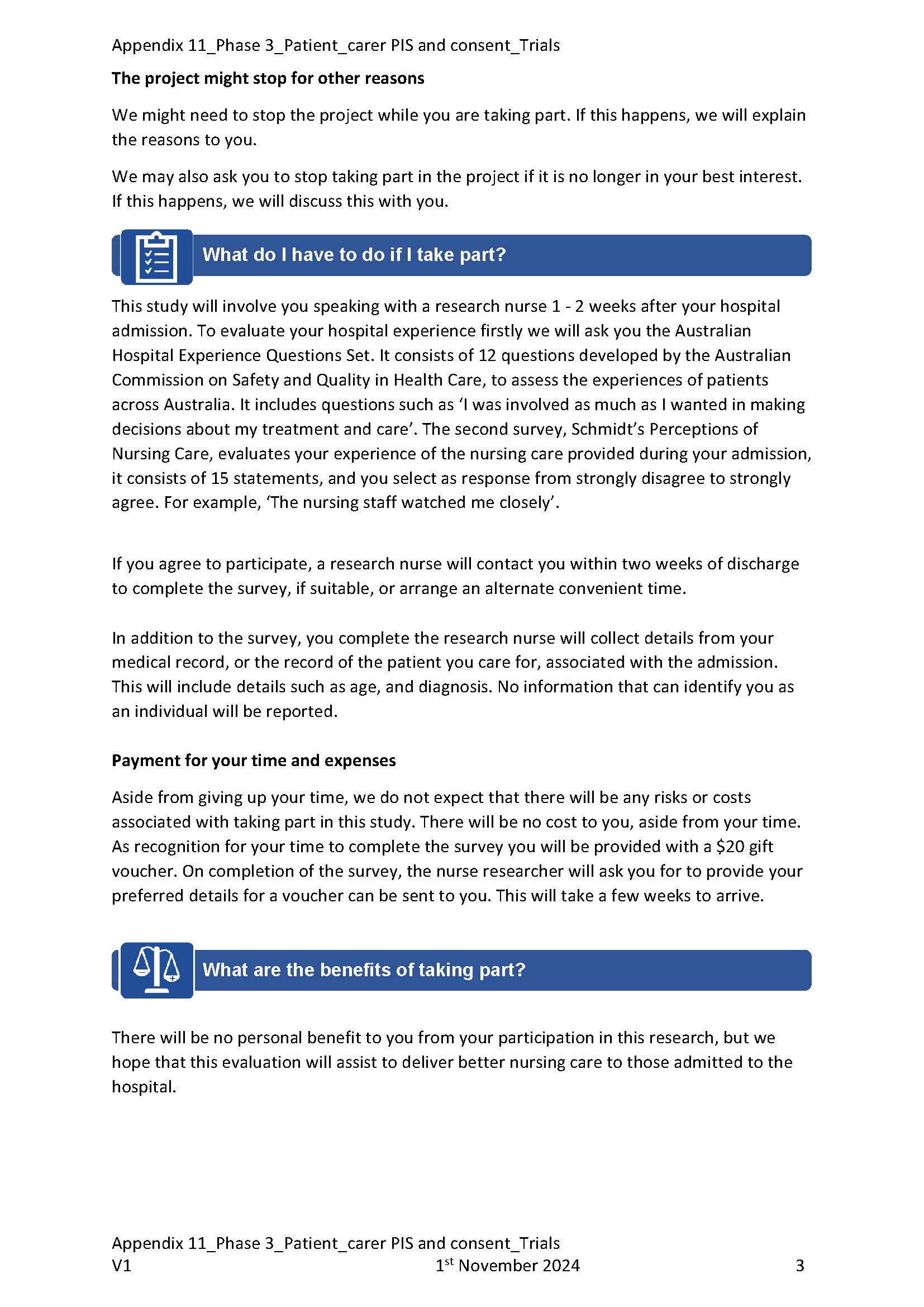


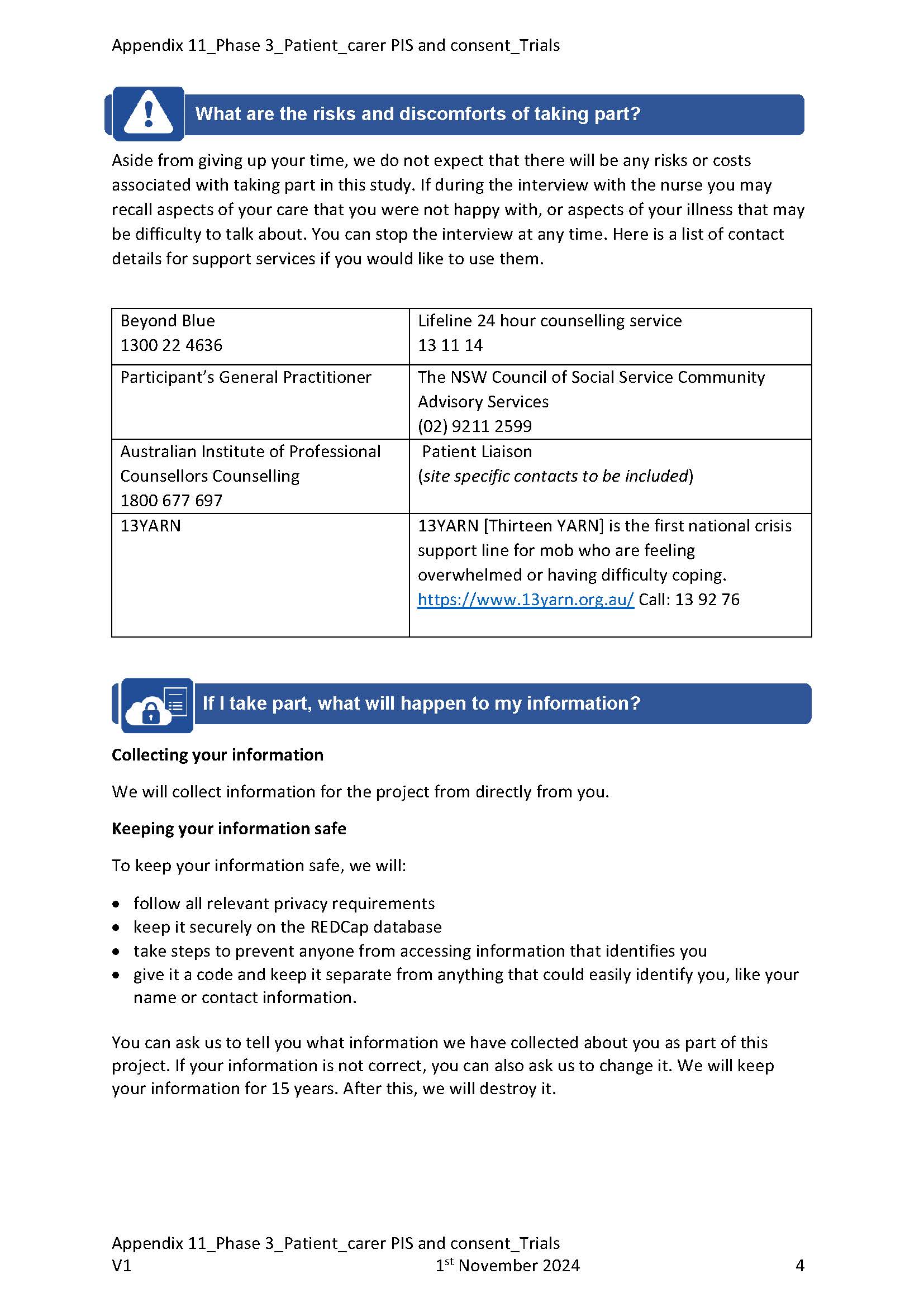


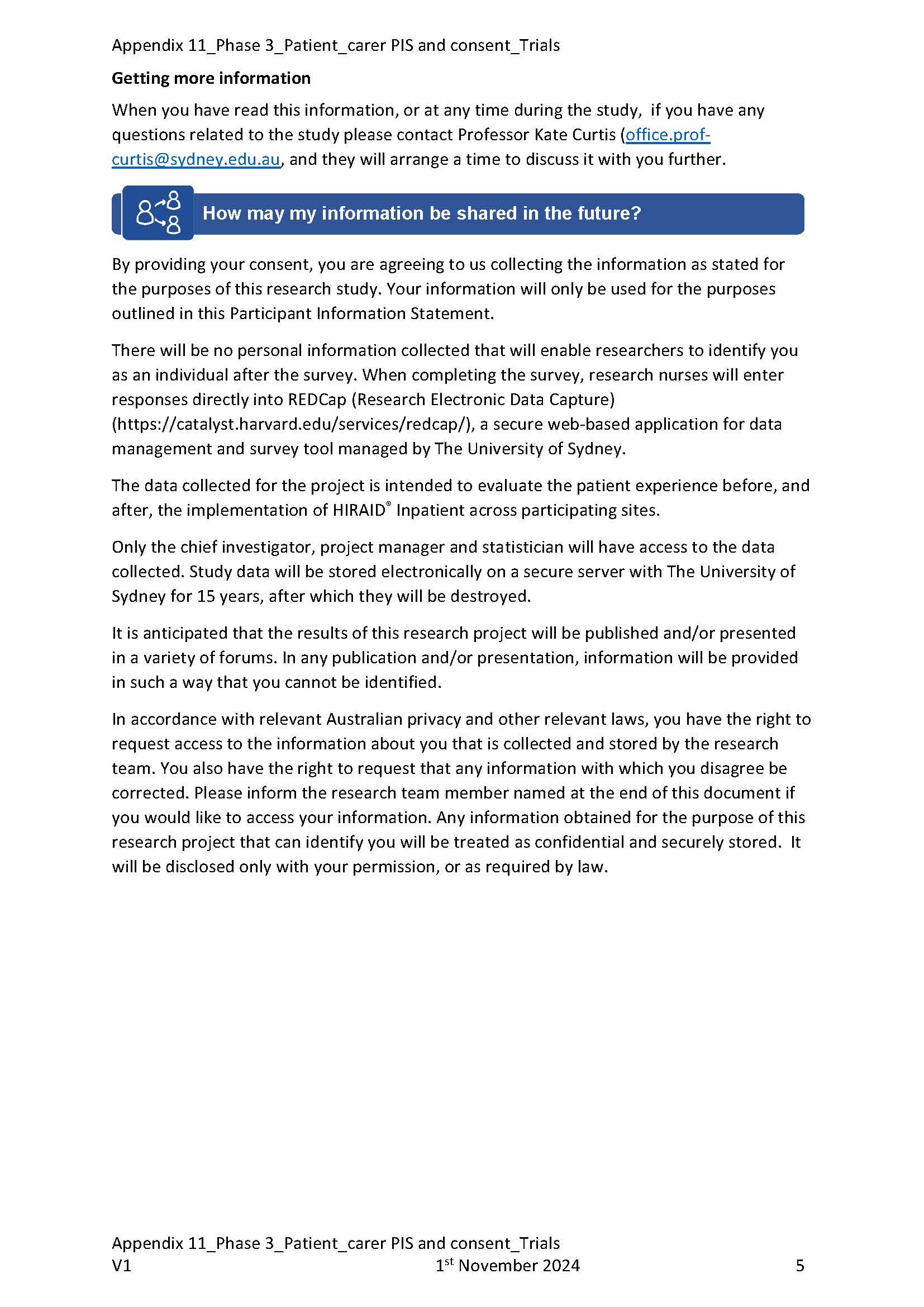


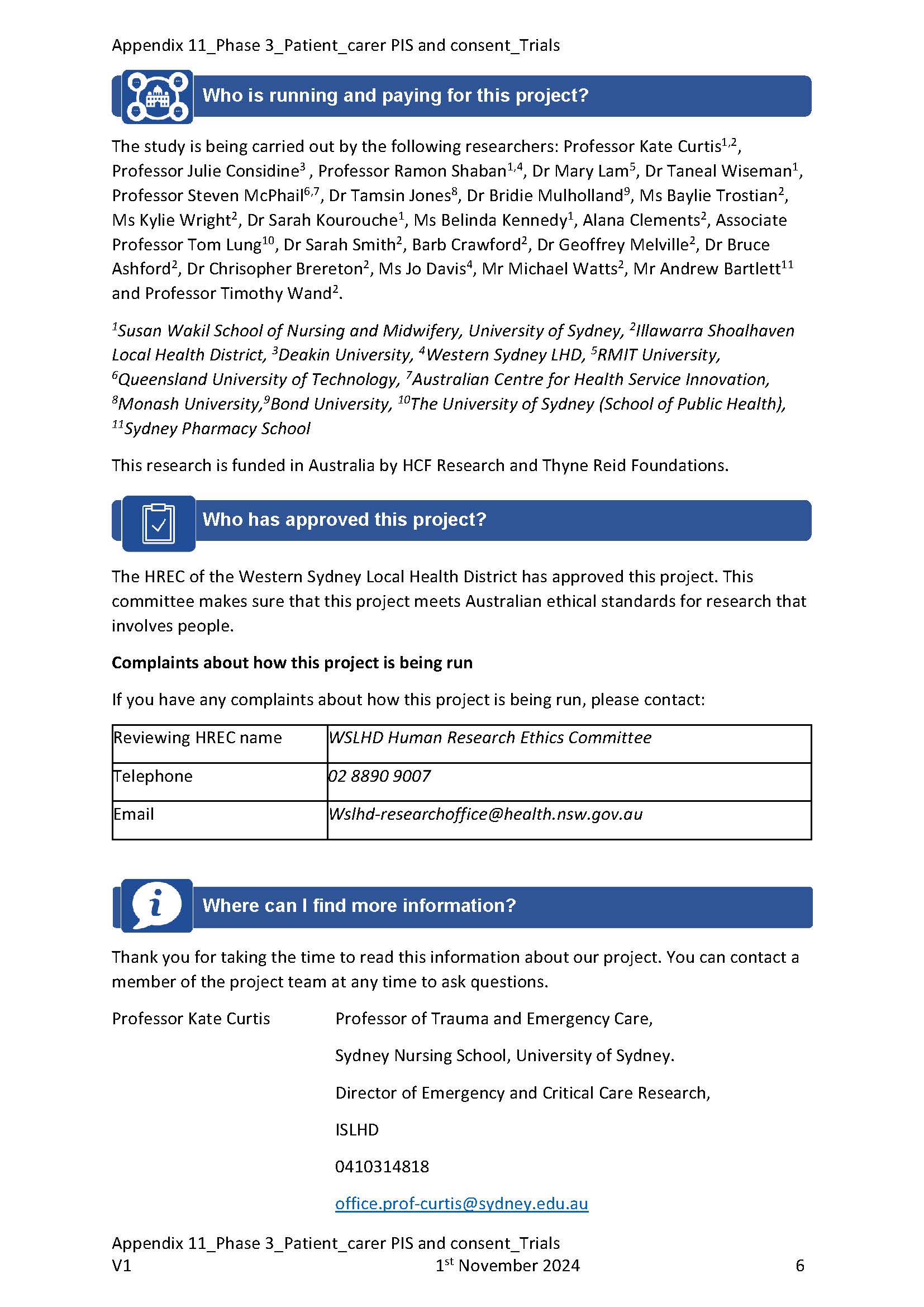


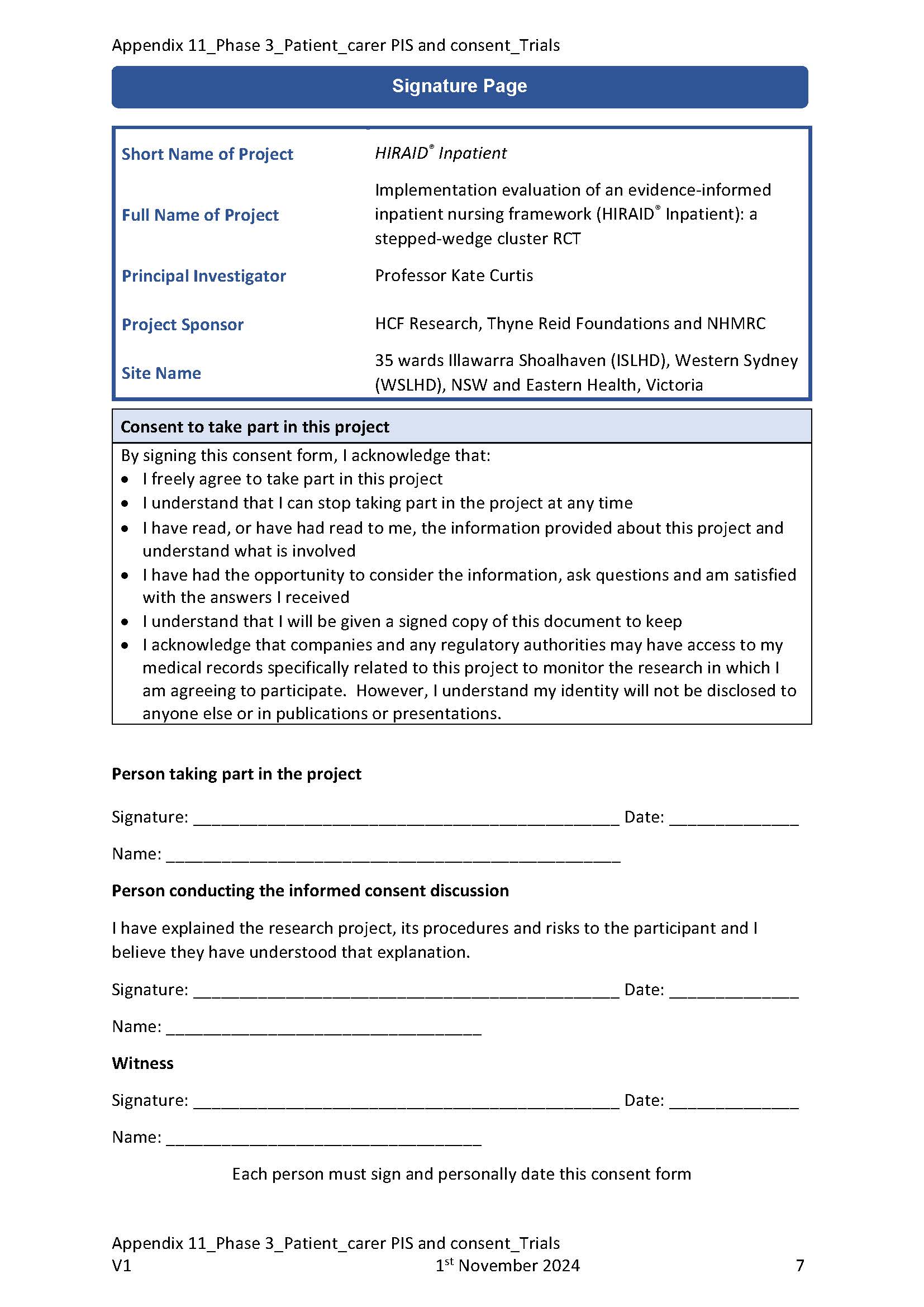

Supplement: Supplementary file 1 — Supplementary Material 1. [file 13063_2025_9313_MOESM1_ESM.docx]
