# Supplementary material for: Implementation evaluation of an evidence-informed hospital inpatient nursing framework (HIRAID® Inpatient): a protocol for a stepped-wedge cluster RCT
Source: Trials. 2025 Dec 4;27:22. doi: 10.1186/s13063-025-09313-8 (PMC12798057; doi:10.1186/s13063-025-09313-8)
Supplement: Supplementary file 2 — Supplementary Material 2. [file 13063_2025_9313_MOESM2_ESM.docx]

# **SPIRIT Checklist for *Trials***

Complete this checklist by entering the page and line numbers where each of the items listed below can be found in your manuscript.

Your manuscript may not currently address all the items on the checklist. Please modify your text to include the missing information. If you are certain that an item does not apply, please state "n/a" and provide a short explanation. **Leaving an item blank or stating “n/a” without an explanation will lead to your manuscript being returned before review.**

Upload your completed checklist as an additional file when you submit to *Trials*. You must reference this additional file in the main text of your protocol submission. The completed SPIRIT figure must be included within the main body of the protocol text and can be downloaded here: <http://www.spirit-statement.org/schedule-of-enrolment-interventions-and-assessments/>

In your methods section, please state that you used the SPIRIT reporting guidelines, and cite them as:

Chan A-W, Tetzlaff JM, Gøtzsche PC, Altman DG, Mann H, Berlin J, Dickersin K, Hróbjartsson A, Schulz KF, Parulekar WR, Krleža-Jerić K, Laupacis A, Moher D. SPIRIT 2013 Explanation and Elaboration: Guidance for protocols of clinical trials. BMJ. 2013;346:e7586

|  |  | **Reporting Item** | **Page and Line Number** | **Reason if not applicable** |
| --- | --- | --- | --- | --- |
| **Administrative information** | | | | |
| Title | [#1](https://www.goodreports.org/reporting-checklists/spirit/info/#1) | Descriptive title identifying the study design, population, interventions, and, if applicable, trial acronym | Page 1, Lines 1-5 | **Heading:** Article title |
| Trial registration | [#2a](https://www.goodreports.org/reporting-checklists/spirit/info/#2a) | Trial identifier and registry name. If not yet registered, name of intended registry | Page 5, Lines 43-45 | **Heading:** Abstract **Subheading:** Trial registration |
| Trial registration: data set | [#2b](https://www.goodreports.org/reporting-checklists/spirit/info/#2b) | All items from the World Health Organization Trial Registration Data Set | Page 5, Lines 43-45 | **Heading:** Abstract **Subheading:** Trial registration |
| Protocol version | [#3](https://www.goodreports.org/reporting-checklists/spirit/info/#3) | Date and version identifier | Page 5, Lines 50-51 | **Heading:** Abstract **Subheading:** Protocol version |
| Funding | [#4](https://www.goodreports.org/reporting-checklists/spirit/info/#4) | Sources and types of financial, material, and other support | Page 39-40, Lines 686-690 | **Heading:** Declarations **Subheading:** Funding |
| Roles and responsibilities: contributorship | [#5a](https://www.goodreports.org/reporting-checklists/spirit/info/#5a) | Names, affiliations, and roles of protocol contributors | Pages 1-3, Lines 6-12 | **Heading:** Names protocol contributors  **Heading:** Author details (table) |
| Roles and responsibilities: sponsor contact information | [#5b](https://www.goodreports.org/reporting-checklists/spirit/info/#5b) | Name and contact information for the trial sponsor | Page 34, Lines 581-591 | **Heading:** Ethics and dissemination **Subheading**: Data monitoring |
| Roles and responsibilities: sponsor and funder | [#5c](https://www.goodreports.org/reporting-checklists/spirit/info/#5c) | Role of study sponsor and funders, if any, in study design; collection, management, analysis, and interpretation of data; writing of the report; and the decision to submit the report for publication, including whether they will have ultimate authority over any of these activities | Page 34, Lines 581-591 | **Heading:** Ethics and dissemination **Subheading**: Data monitoring |
| Roles and responsibilities: committees | [#5d](https://www.goodreports.org/reporting-checklists/spirit/info/#5d) | Composition, roles, and responsibilities of the coordinating centre, steering committee, endpoint adjudication committee, data management team, and other individuals or groups overseeing the trial, if applicable (see Item 21a for data monitoring committee) | Page 34, Lines 581-591 | **Heading:** Ethics and dissemination **Subheading:** Data monitoring |
| **Introduction** |  |  | Page 5-8, Lines 55-110 | **Heading**: Introduction |
| Background and rationale | [#6a](https://www.goodreports.org/reporting-checklists/spirit/info/#6a) | Description of research question and justification for undertaking the trial, including summary of relevant studies (published and unpublished) examining benefits and harms for each intervention | Page 5-6, Lines 56-78 | **Heading**: Introduction  **Subheading**: Background |
| Background and rationale: choice of comparators | [#6b](https://www.goodreports.org/reporting-checklists/spirit/info/#6b) | Explanation for choice of comparators | Page 6-8, Lines 79-107 | **Heading:** Phase 3: Implementation evaluation of HIRAID® Inpatient **Subheadings 2:** Study design |
| Objectives | [#7](https://www.goodreports.org/reporting-checklists/spirit/info/#7) | Specific objectives or hypotheses | Page 15, Lines 269-273 | **Heading:** Phase 3: Implementation evaluation of HIRAID® Inpatient **Subheading**: Hypotheses |
| Trial design | [#8](https://www.goodreports.org/reporting-checklists/spirit/info/#8) | Description of trial design including type of trial (eg, parallel group, crossover, factorial, single group), allocation ratio, and framework (eg, superiority, equivalence, non-inferiority, exploratory) | Page 14, Lines 248-268 | **Heading:** Phase 3: implementation evaluation of HIRAID® inpatient **Subheading:** Study design |
| **Methods: Participants, interventions, and outcomes** | | | | |
| Study setting | [#9](https://www.goodreports.org/reporting-checklists/spirit/info/#9) | Description of study settings (eg, community clinic, academic hospital) and list of countries where data will be collected. Reference to where list of study sites can be obtained | Pages 15-16, Lines 273-283 | **Heading:** Phase 3: implementation evaluation of HIRAID® inpatient **Subheading:** Study design  **Reference point:** Table 2, Information added |
| Eligibility criteria | [#10](https://www.goodreports.org/reporting-checklists/spirit/info/#10) | Inclusion and exclusion criteria for participants. If applicable, eligibility criteria for study centres and individuals who will perform the interventions (eg, surgeons, psychotherapists) | Page 16-17, Lines 283-309 | **Heading:** Phase 3: implementation evaluation of HIRAID® inpatient **Subheading:** Participants and eligibility criteria |
| Interventions: description | [#11a](https://www.goodreports.org/reporting-checklists/spirit/info/#11a) | Interventions for each group with sufficient detail to allow replication, including how and when they will be administered | Pages 17-20, Lines 310-365 | **Heading:** Phase 3: Implementation evaluation of HIRAID® Inpatient  **Subheadings**: Randomisation; Blinding; Study Plan |
| Interventions: modifications | [#11b](https://www.goodreports.org/reporting-checklists/spirit/info/#11b) | Criteria for discontinuing or modifying allocated interventions for a given trial participant (eg, drug dose change in response to harms, participant request, or improving / worsening disease) | Page 18-20, Lines 325-365  Pages 34-35, Lines 592-604 | **Heading:** Phase 3: Implementation evaluation of HIRAID® Inpatient **Subheading:** Study Plan  **Heading:** Ethics and dissemination **Subheading:** Harm |
| Interventions: adherance | [#11c](https://www.goodreports.org/reporting-checklists/spirit/info/#11c) | Strategies to improve adherence to intervention protocols, and any procedures for monitoring adherence (eg, drug tablet return; laboratory tests) | Pages 11-14, Lines 171-246  Page 21, Lines 383-392  Page 27, Lines 464-469 Pages 34-35, Lines 580-611 | **Heading:** Phase 2: Explanatory sequential mixed methods study to design HIRAID® Inpatient Implementation Strategy  **Heading:** Phase 3: implementation evaluation of HIRAID® inpatient  **Subheading**: Outcomes **Subheading:** Inpatients (or their carers) experience with nursing care  **Heading:** Ethics and Dissemination **Subheading:** Data monitoring  **Heading:** Phase 3: implementation evaluation of HIRAID® inpatient  **Subheading:** Data collection |
| Interventions: concomitant care | [#11d](https://www.goodreports.org/reporting-checklists/spirit/info/#11d) | Relevant concomitant care and interventions that are permitted or prohibited during the trial | Page 18, Lines 353 - 360 | **Heading:** Phase 3: implementation evaluation of HIRAID® inpatient  **Subheading:** Study design |
| Outcomes | [#12](https://www.goodreports.org/reporting-checklists/spirit/info/#12) | Primary, secondary, and other outcomes, including the specific measurement variable (eg, systolic blood pressure), analysis metric (eg, change from baseline, final value, time to event), method of aggregation (eg, median, proportion), and time point for each outcome. Explanation of the clinical relevance of chosen efficacy and harm outcomes is strongly recommended | Pages 20-26, Lines 366-433  Pages 26-30 Lines 436-539 | **Heading:** Phase 3: Implementation evaluation of HIRAID® Inpatient **Subheading:** Outcomes  **Heading:** Phase 3: Implementation evaluation of HIRAID® Inpatient **Subheading:** Data management, Figure 5 |
| Participant timeline | [#13](https://www.goodreports.org/reporting-checklists/spirit/info/#13) | Time schedule of enrolment, interventions (including any run-ins and washouts), assessments, and visits for participants. A schematic diagram is highly recommended (see Figure) | Pages 22-24, Lines 419-420  Pages 32-33, Lines 577-579 | **Heading:** Outcomes  **Heading:** Statistical methods **Subheading:** Data management |
| Sample size | [#14](https://www.goodreports.org/reporting-checklists/spirit/info/#14) | Estimated number of participants needed to achieve study objectives and how it was determined, including clinical and statistical assumptions supporting any sample size calculations | Pages 24-26, Lines 421-436 | **Heading:** Sample size |
| Recruitment | [#15](https://www.goodreports.org/reporting-checklists/spirit/info/#15) | Strategies for achieving adequate participant enrolment to reach target sample size | Pages 9-10, Lines 131-157  Page 12, Lines 188-207  Page 16-19, Lines 283- 362  Pages 26-27, Lines 436-457 | **Heading:** Phase 1: Delphi study: adaptation of HIRAID® Inpatient **Subheading 2:** Data collection  **Heading:** Phase 2: Explanatory sequential mixed methods study to design HIRAID® Inpatient Implementation Strategy **Subheading 2:** Participants and eligibility criteria  **Heading:** Phase 2: Explanatory sequential mixed methods study to design HIRAID® Inpatient Implementation Strategy **Subheading:** Data collection  **Heading:** Phase 3: Implementation evaluation of HIRAID® Inpatient **Subheading:** Data collection |
| **Methods: Assignment of interventions (for controlled trials)** | | | | |
| Allocation: sequence generation | [#16a](https://www.goodreports.org/reporting-checklists/spirit/info/#16a) | Method of generating the allocation sequence (eg, computer-generated random numbers), and list of any factors for stratification. To reduce predictability of a random sequence, details of any planned restriction (eg, blocking) should be provided in a separate document that is unavailable to those who enrol participants or assign interventions | Pages 17-18, Lines 310-324 | **Heading:** Randomisation  **Heading:** Blinding |
| Allocation concealment mechanism | [#16b](https://www.goodreports.org/reporting-checklists/spirit/info/#16b) | Mechanism of implementing the allocation sequence (eg, central telephone; sequentially numbered, opaque, sealed envelopes), describing any steps to conceal the sequence until interventions are assigned | Pages 17-18, Lines 310-324 | **Heading:** Randomisation  **Heading:** Blinding |
| Allocation: implementation | [#16c](https://www.goodreports.org/reporting-checklists/spirit/info/#16c) | Who will generate the allocation sequence, who will enrol participants, and who will assign participants to interventions | Pages 9-11 Lines 131-170  Pages 11-14, Lines 181- 246  Pages 16-18, Lines 283-324 | **Heading:** Phase 1: Delphi study: adaptation of HIRAID® Inpatient  **Subheading:** Data collection  **Heading:** Phase 2: Explanatory sequential mixed methods study to design HIRAID® Inpatient Implementation Strategy  **Subheading:** Participants and eligibility criteria  **Heading:** Phase 3: Implementation evaluation of HIRAID® Inpatient  **Subheading:** Participants and eligibility criteria |
| Blinding (masking) | [#17a](https://www.goodreports.org/reporting-checklists/spirit/info/#17a) | Who will be blinded after assignment to interventions (eg, trial participants, care providers, outcome assessors, data analysts), and how | Pages 17-18, Lines 310-324 | **Heading:** Randomisation  **Heading:** Blinding |
| Blinding (masking): emergency unblinding | [#17b](https://www.goodreports.org/reporting-checklists/spirit/info/#17b) | If blinded, circumstances under which unblinding is permissible, and procedure for revealing a participant’s allocated intervention during the trial | Pages 17-18, Lines 310-324 | **Heading:** Randomisation  **Heading:** Blinding |
| **Methods: Data collection, management, and analysis** | | | | |
| Data collection plan | [#18a](https://www.goodreports.org/reporting-checklists/spirit/info/#18a) | Plans for assessment and collection of outcome, baseline, and other trial data, including any related processes to promote data quality (eg, duplicate measurements, training of assessors) and a description of study instruments (eg, questionnaires, laboratory tests) along with their reliability and validity, if known. Reference to where data collection forms can be found, if not in the protocol | Pages 9-11 Lines 131-170  Pages 12-14, Lines 188- 246  Pages 16-18, Lines 283-324 | **Heading:** Phase 1: Delphi study: adaptation of HIRAID® Inpatient  **Subheading:** Data collection  **Heading:** Phase 2: Explanatory sequential mixed methods study to design HIRAID® Inpatient Implementation Strategy  **Subheading:** Data collection  **Heading:** Phase 3: Implementation evaluation of HIRAID® Inpatient  **Subheading:** Participants and eligibility criteria |
| Data collection plan: retention | [#18b](https://www.goodreports.org/reporting-checklists/spirit/info/#18b) | Plans to promote participant retention and complete follow-up, including list of any outcome data to be collected for participants who discontinue or deviate from intervention protocols | Pages 18-20, Lines 335-365  Pages 34-35, Lines 581-611 | **Heading:** Phase 3: Implementation evaluation of HIRAID® Inpatient **Subheading:** Study Plan  **Heading:** Phase 3: Implementation evaluation of HIRAID® Inpatient **Subheading:** Ethics and dissemination |
| Data management | [#19](https://www.goodreports.org/reporting-checklists/spirit/info/#19) | Plans for data entry, coding, security, and storage, including any related processes to promote data quality (eg, double data entry; range checks for data values). Reference to where details of data management procedures can be found, if not in the protocol | Pages 32-35, Lines 565-611  Pages 18-20, Lines 335-365 | **Heading:** Phase 3: Implementation evaluation of HIRAID® Inpatient **Subheadings:** Data management, Study Plan, Ethics and dissemination |
| Statistics: outcomes | [#20a](https://www.goodreports.org/reporting-checklists/spirit/info/#20a) | Statistical methods for analysing primary and secondary outcomes. Reference to where other details of the statistical analysis plan can be found, if not in the protocol | Page 31, Lines 540-564  Pages 20 - 24, Lines 366-420 | **Heading:** Phase 3: Implementation evaluation of HIRAID® Inpatient **Subheading:** Statistical methods  **Heading:** Phase 3: Implementation evaluation of HIRAID® Inpatient **Subheading:** Outcomes  Table 3: Summary of HIRAID® Inpatient outcome measure, data collection sources and measurement timepoints. |
| Statistics: additional analyses | [#20b](https://www.goodreports.org/reporting-checklists/spirit/info/#20b) | Methods for any additional analyses (eg, subgroup and adjusted analyses) | No applicable | No additional analyses |
| Statistics: analysis population and missing data | [#20c](https://www.goodreports.org/reporting-checklists/spirit/info/#20c) | Definition of analysis population relating to protocol non-adherence (eg, as randomised analysis), and any statistical methods to handle missing data (eg, multiple imputation) | Page 31, Lines 540-564 | **Heading:** Phase 3: Implementation evaluation of HIRAID® Inpatient **Subheading**: Statistical methods |
| **Methods: Monitoring** | | | | |
| Data monitoring: formal committee | [#21a](https://www.goodreports.org/reporting-checklists/spirit/info/#21a) | Composition of data monitoring committee (DMC); summary of its role and reporting structure; statement of whether it is independent from the sponsor and competing interests; and reference to where further details about its charter can be found, if not in the protocol. Alternatively, an explanation of why a DMC is not needed | Page 34-35, Lines 580- 611 | **Heading:** Ethics and dissemination |
| Data monitoring: interim analysis | [#21b](https://www.goodreports.org/reporting-checklists/spirit/info/#21b) | Description of any interim analyses and stopping guidelines, including who will have access to these interim results and make the final decision to terminate the trial | Page 34-35, Lines 580- 611 | **Heading:** Ethics and dissemination |
| Harms | [#22](https://www.goodreports.org/reporting-checklists/spirit/info/#22) | Plans for collecting, assessing, reporting, and managing solicited and spontaneously reported adverse events and other unintended effects of trial interventions or trial conduct | Page 34-35, Lines 580- 611 | **Heading:** Ethics and Dissemination |
| Auditing | [#23](https://www.goodreports.org/reporting-checklists/spirit/info/#23) | Frequency and procedures for auditing trial conduct, if any, and whether the process will be independent from investigators and the sponsor | Page 31, Lines 540-564 | **Heading:** Phase 3: Implementation evaluation of HIRAID® Inpatient **Subheading:** Implementation fidelity audits. |
| **Ethics and dissemination** | | | | |
| Research ethics approval | [#24](https://www.goodreports.org/reporting-checklists/spirit/info/#24) | Plans for seeking research ethics committee / institutional review board (REC / IRB) approval | Pages 5, Lines 46-49  Pages 34-35, Lines 580- 611 | **Heading:** Abstract  **Heading:** Ethics and Dissemination |
| Protocol amendments | [#25](https://www.goodreports.org/reporting-checklists/spirit/info/#25) | Plans for communicating important protocol modifications (eg, changes to eligibility criteria, outcomes, analyses) to relevant parties (eg, investigators, REC / IRBs, trial participants, trial registries, journals, regulators) | Pages 39-40, Lines 667-705  Pages 34-35, Lines 580-604 | **Heading:** Declarations **Subheading:** Ethics approval and consent  **Heading:** Ethics and dissemination **Subheading:** Data monitoring |
| Consent or assent | [#26a](https://www.goodreports.org/reporting-checklists/spirit/info/#26a) | Who will obtain informed consent or assent from potential trial participants or authorised surrogates, and how (see Item 32) | Pages 39-40, Lines 667-705  Page 5, Lines 46-49 | **Heading:** Abstract **Subheading:** Ethics and dissemination  **Heading:** Declarations **Subheading:** Ethics approval and consent |
| Consent or assent: ancillary studies | [#26b](https://www.goodreports.org/reporting-checklists/spirit/info/#26b) | Additional consent provisions for collection and use of participant data and biological specimens in ancillary studies, if applicable | Page 5, Lines 46-49  Pages 39-40, Lines 667-705 | **Heading:** Abstract **Subheading:** Ethics and dissemination  **Heading:** Declarations **Subheading:** Ethics approval and consent |
| Confidentiality | [#27](https://www.goodreports.org/reporting-checklists/spirit/info/#27) | How personal information about potential and enrolled participants will be collected, shared, and maintained in order to protect confidentiality before, during, and after the trial | Page 17, Lines 294-306  Pages 39-40, Lines 667-705  Pages 9-11 Lines 131-170  Pages 12-14, Lines 188- 246  Pages 16-18, Lines 283-324  Pages 21-22, Lines 407-418  Page 32, Lines 571- 577 | **Heading:** Participants and eligibility criteria **Subheading**: Inpatients (or their carers) of a study ward  **Heading:** Declarations **Subheading:** Ethics approval and consent    **Heading:** Phase 1: Delphi study: adaptation of HIRAID® Inpatient **Subheading**: Data collection  **Heading:** Phase 2: Explanatory sequential mixed methods study to design HIRAID® Inpatient Implementation Strategy **Subheading:** Data collection  **Heading:** Phase 3: Implementation evaluation of HIRAID® Inpatient **Subheading:** Participants and eligibility criteria  **Heading:** Outcomes **Subheading:** Implementation fidelity  **Heading**: Data management **Subheading:** Data de-identification and linkage |
| Declaration of interests | [#28](https://www.goodreports.org/reporting-checklists/spirit/info/#28) | Financial and other competing interests for principal investigators for the overall trial and each study site | Pages 39-40, Lines 667-705 | **Heading:** Declarations |
| Data access | [#29](https://www.goodreports.org/reporting-checklists/spirit/info/#29) | Statement of who will have access to the final trial dataset, and disclosure of contractual agreements that limit such access for investigators | Pages 34-35, Lines 580-604  Pages 39, Lines 682-683 | **Heading:** Ethics and dissemination  **Heading:** Availability of data and materials |
| Ancillary and post trial care | [#30](https://www.goodreports.org/reporting-checklists/spirit/info/#30) | Provisions, if any, for ancillary and post-trial care, and for compensation to those who suffer harm from trial participation | Pages 34-35, Lines 580-604 | **Heading:** Ethics and dissemination |
| Dissemination policy: trial results | [#31a](https://www.goodreports.org/reporting-checklists/spirit/info/#31a) | Plans for investigators and sponsor to communicate trial results to participants, healthcare professionals, the public, and other relevant groups (eg, via publication, reporting in results databases, or other data sharing arrangements), including any publication restrictions | Page 5, Lines 46-49  Pages 34-35, Lines 580-604 | **Heading:** Abstract  **Heading:** Ethics and dissemination |
| Dissemination policy: authorship | [#31b](https://www.goodreports.org/reporting-checklists/spirit/info/#31b) | Authorship eligibility guidelines and any intended use of professional writers | Pages 34-35, Lines 580-604 | **Heading:** Ethics and dissemination |
| Dissemination policy: reproducible research | [#31c](https://www.goodreports.org/reporting-checklists/spirit/info/#31c) | Plans, if any, for granting public access to the full protocol, participant-level dataset, and statistical code | Pages 34-35, Lines 580-604 | **Heading:** Ethics and dissemination |
| **Appendices** | | | | |
| Informed consent materials | [#32](https://www.goodreports.org/reporting-checklists/spirit/info/#32) | Model consent form and other related documentation given to participants and authorised surrogates | Pages 34-35, Lines 580-604  Page 39, Lines 679-681  Supplementary material Pages 28-48 | **Heading:** Ethics and dissemination  **Heading:** Declarations  Supplement 3 and 4 |
| Biological specimens | [#33](https://www.goodreports.org/reporting-checklists/spirit/info/#33) | Plans for collection, laboratory evaluation, and storage of biological specimens for genetic or molecular analysis in the current trial and for future use in ancillary studies, if applicable | Not applicable | No biological specimens collected, and no genetic or molecular analysis planned. |

It is strongly recommended that this checklist be read in conjunction with the SPIRIT 2013 Explanation & Elaboration for important clarification on the items. Amendments to the protocol should be tracked and dated. The SPIRIT checklist is copyrighted by the SPIRIT Group under the Creative Commons “[Attribution-NonCommercial-NoDerivs 3.0 Unported](http://www.creativecommons.org/licenses/by-nc-nd/3.0/)” license. This checklist can be completed online using https://www.goodreports.org/, a tool made by the EQUATOR Network in collaboration with Penelope.ai
